# Iron‐Catalyzed Reactions of 2‐Pyridone Derivatives: 1,6‐Addition and Formal Ring Opening/Cross Coupling

**DOI:** 10.1002/asia.201900865

**Published:** 2019-07-31

**Authors:** Lin Huang, Yiting Gu, Alois Fürstner

**Affiliations:** ^1^ Max-Planck-Institut für Kohlenforschung 45470 Mülheim/Ruhr Germany

**Keywords:** 1,6-addition, cross coupling, dienes, heterocycles, iron catalysis, ring cleavage

## Abstract

In the presence of simple iron salts, 2‐pyridone derivatives react with Grignard reagents under mild conditions to give the corresponding 1,6‐addition products; if the reaction medium is supplemented with an aprotic dipolar cosolvent after the actual addition step, the intermediates primarily formed succumb to ring opening, giving rise to non‐thermodynamic *Z,E*‐configured dienoic acid amide derivatives which are difficult to make otherwise. Control experiments as well as the isolation and crystallographic characterization of a (tricarbonyl)iron pyridone complex suggest that the active iron catalyst generated in situ exhibits high affinity to the polarized diene system embedded into the heterocyclic ring system of the substrates, which likely serves as the actual recognition element.

## Graphical abstract

**Keep it closed or lock it open**: The unique ability of simple iron salts to catalyze the 1,6‐addition of Grignard reagents to 2‐pyridones is likely due to the affinity of the active iron species generated in situ to the polarized diene subunit of the substrates. When the reaction medium is complemented with an aprotic dipolar cosolvent, C−C bond formation is followed by opening of the heterocyclic ring.

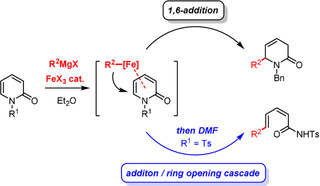

## Introduction

**Scheme 1:**
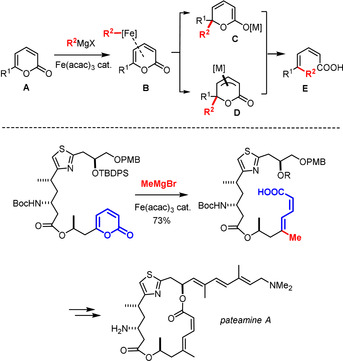
Iron‐catalyzed formal ring opening/cross coupling of pyrones.

During our investigations into cross coupling, cycloaddition and cycloisomerization reactions using cheap, practical, non‐toxic and benign iron catalysts,[^1^, ^2^, ^3^, ^4^] we discovered that 2‐pyrones **A** undergo an unusual transformation when treated with Grignard reagents in the presence of catalytic Fe(acac)_3_ at low temperature (Scheme 1). These substrates swiftly convert into dienoic acid derivatives **E** by what appears to be a ring opening/cross coupling reaction, in which the enol ester subunit of the heterocyclic ring formally gains the role of a leaving group that is replaced by the incoming nucleophile with retention of configuration.^[5]^ Uncatalyzed attack of the organomagnesium reagent on the lactone carbonyl group of **A** is too slow to be competitive^[6]^ and the non‐thermodynamic *Z,E*‐configured 1,3‐diene motif of the resulting product **E** usually persists under the mild conditions.^[7]^ Moreover, the low‐valent iron catalyst generated in situ proved compatible with numerous functional groups, including heteroatom donor sites that interfere with more traditional cross coupling. For its favorable profile and the ready availability of differently substituted pyrone derivatives,[^8^, ^9^] this unorthodox transformation laid the ground for concise approaches to the marine natural product pateamine A and its almost equipotent analog DMDA‐Pat A, in that it allowed the very sensitive but critically important dienoate subunit of these highly cytotoxic agents to be unveiled at a late stage from a robust heterocyclic precursor.[^10^, ^11^]

Indirect evidence, however, suggested that the net outcome of the reaction is probably not the result of a true cross coupling manifold; rather, the reaction is thought to proceed via an initial 1,6‐addition followed by ring opening of the resulting intermediate of type **C** or **D**.[^5^, ^10^, ^12^] This latter step could proceed in an electrocyclic or ionic fashion; in any case, it must be fast and facile even at low temperature, because the proposed intermediates defied all attempts at characterization. Provided that this mechanistic hypothesis is valid, is should be possible to engage other heterocyclic compounds containing a polarized diene entity into similar transformations.

## Results and Discussion

2‐Pyridones are obvious and relevant candidates:[^13^, ^14^, ^15^] the enamide embedded in their ring system is certainly not a privileged subunit for classical cross coupling via oxidative insertion/reductive elimination, whereas an iron catalyzed 1,6‐addition is feasible since acyclic α,β,γ,δ‐unsaturated amides have previously been shown to react with aryl‐Grignard reagents in the presence of catalytic amounts of FeCl_2_ with high selectivity.[^16^, ^17^] Indeed, the *N*‐benzylated substrate **1**, on treatment with PhMgBr and Fe(acac)_3_ (5 mol %), gave product **2 a** in which the heterocyclic ring is intact (Table 1); under optimized conditions (THF, −45 °C), the yield was almost quantitative (entry 6). Fe(acac)_3_, Fe(acac)_2_ and FeCl_3_ work almost equally well,^[18]^ whereas the reaction did not proceed with FeF_3_ or in the absence of an iron precatalyst.^[19]^

**Table 1:** Reaction optimization and gathering of mechanistically relevant information (entries 10–15, see below).^[a]^

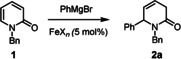				
Entry	FeX_*n*_	Solvent	*T* [°C]	2^[b]^
1	Fe(acac)_3_	Et_2_O	−78	19
2	Fe(acac)_3_	Et_2_O	−45	69
3	Fe(acac)_3_	Et_2_O	−20	46^[c]^
4	Fe(acac)_3_	toluene	−45	78
5	Fe(acac)_3_	THF	−45	94 (83)^[d]^
6	Fe(acac)_3_	THF	−45	96^[e,f]^
7	FeCl_3_	THF	−45	93
8	FeF_3_	THF	−45	n.r.
9	Fe(acac)_2_	THF	−45	89
10	[(Me_4_Fe)⋅(MeLi)][Li⋅(OEt_2_)]_2_	THF	−45→RT	78
11	Fe(CO)_5_	THF	−45→RT	78
12	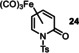	THF	−45→RT	65
13	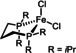	THF	−45→RT	99
14	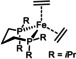	THF	−45→RT	45
15	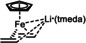	THF	−45→RT	75

[a] unless stated otherwise, all reactions were carried out using PhMgBr (3 equiv); [b] determined by NMR, unless stated otherwise; [c] small amounts of unidentified byproducts were detected in the crude mixture; [d] using PhMgCl; [e] isolated yield; [f] using only 1.5 equivalents of PhMgBr.

**Scheme 2:**
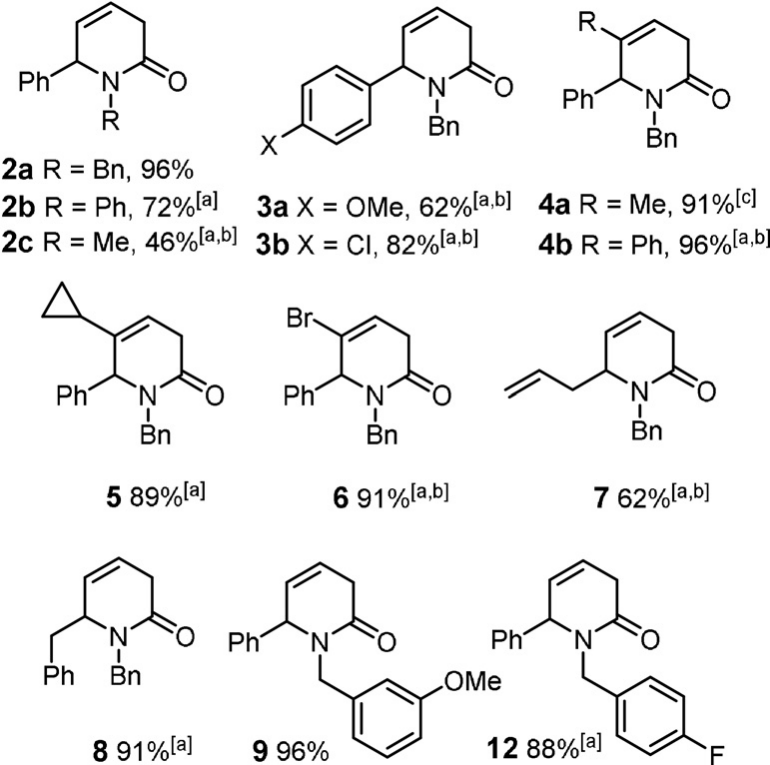
Scope of the iron‐catalyzed 1,6‐addition reaction to 2‐pyridones; unless stated otherwise, all reaction were performed with RMgBr (1.5 equiv), Fe(acac)_3_ (5 mol %) in THF at −45 °C; [a] with 3 equiv of RMgX; [b] at −20 °C; [c] at −45 °C to RT.

**Scheme 3:**
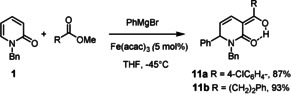
Iron‐catalyzed three component coupling reactions.

As evident from the examples shown in Scheme 2, the reaction is tolerant of different polar and apolar functionalities in the substrate as well as in the Grignard reagent. The arguably most notable case is product **6** derived from 1‐benzyl‐5‐bromopyridin‐2(1*H*)‐one: the fact that the bromide substituent persists under the chosen reaction conditions implies that the iron catalyzed addition reaction to the heterocyclic ring is even faster than a regular iron catalyzed cross coupling, although the latter process is known to proceed very rapidly at low temperatures;[^20^, ^21^, ^22^, ^23^] such stunning “inverse” chemoselectivity is exceedingly rare.^[24]^ Since spontaneous ring opening did not occur, it was also possible to intercept the enolate primarily formed by 1,6‐addition with an appropriate electrophilic partner (Scheme 3). The high yielding preparation of compounds **11 a**,**b** illustrates this point (for the structure of compound **11 a** in the solid state, see the SI). Once again it is remarkable that the iron catalyzed 1,6‐additon to the 2‐pyridone outcompetes the uncatalyzed reaction of the Grignard reagent with the (aromatic or aliphatic) ester present in the mixture; likewise, the reaction is faster than cross coupling with the chloride substituent of methyl chlorobenzoate used to make **11 a**, which is known to be an excellent substrate otherwise.[^25^, ^26^, ^27^]

**Scheme 4:**
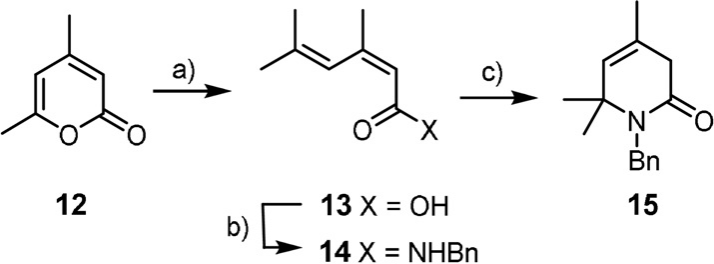
a) MeMgBr, Fe(acac)_3_ (5 mol %), Et_2_O, −30 °C, 93 %; b) BnNH_2_, HOBt, EDC⋅HCl, Et_3_N, CH_2_Cl_2_, DMF, 92 %; c) LiHMDS, DMF, 100 °C, 67 %; EDC=1‐ethyl‐3‐(3‐dimethylaminopropyl)carbodiimide; HOBt=1‐hydroxybenzotriazole; LiHMDS=lithium hexamethyldisilazide.

**Scheme 5:**
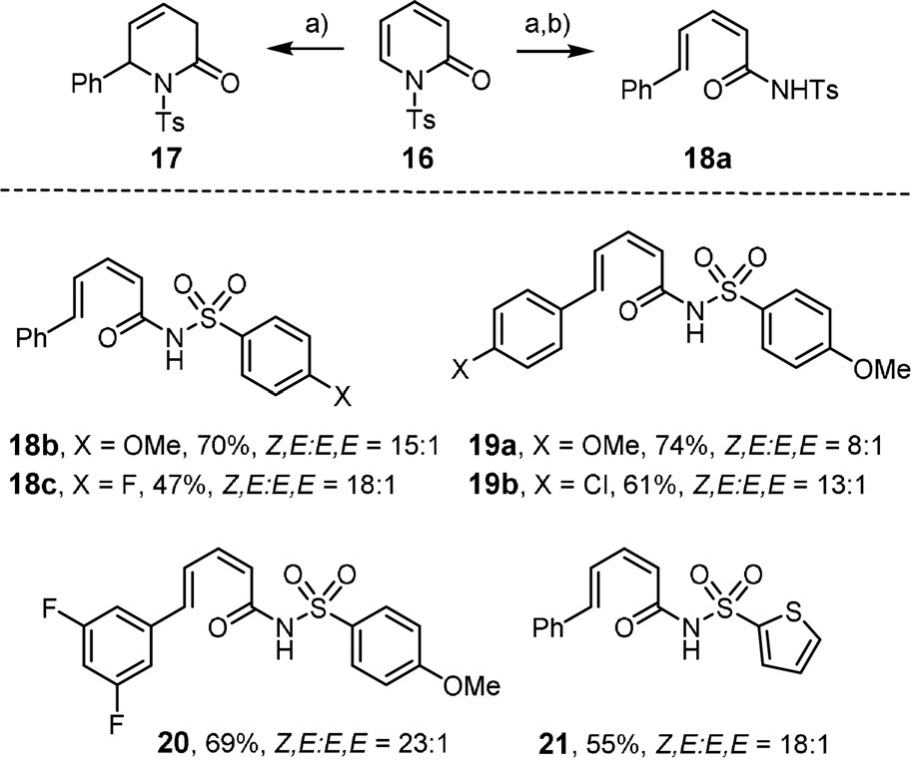
a) PhMgBr, Fe(acac)_3_ (5 mol %), Ph_3_P (10 mol %), Et_2_O, −30 °C, 62 %; b) DMF, −30 °C→−10 °C, 60 % (over both steps), *Z,E*:*E,E*≥15:1.

**Figure 1:**
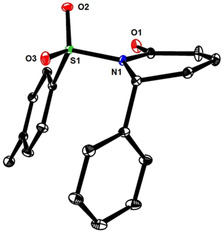
Structure of compound **17** in the solid state; H‐atoms are omitted for clarity.

**Figure 2:**
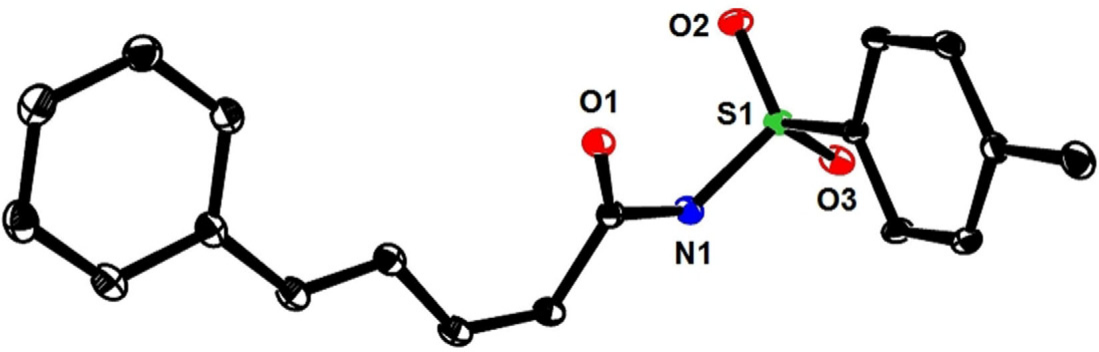
Structure of compound **18 a** in the solid state; H‐atoms are omitted for clarity.

These results are consistent with a 1,6‐addition reaction as the actual iron catalyzed event; it remained to be clarified, however, why this step is followed by instantaneous ring opening in the pyrone series but not with 2‐pyridones such as **1**. To this end, we transformed the pyrone‐derived dienoic acid **13** into the corresponding amide **14** (Scheme 4); on deprotonation with LiHMDS and warming of the resulting mixture, **14** cyclized to the corresponding dihydropyridin‐2‐one **15**. Since this control experiment suggested that the cyclic form is thermodynamically more stable in this case, attempts were made to change the tip in favor of the ring opened product by altering the *N*‐substituent. Based on literature precedent,^[28]^ the *N*‐tosyl derivative **16** was deemed adequate (the structure of **16** in the solid state is contained in the SI) (Scheme 5): initially, however, only the 1,6‐adduct **17** was attained on reaction with PhMgBr and catalytic Fe(acac)_3_. Under standard conditions, the conversion remained incomplete, but the outcome could be improved in the presence of PPh_3_ (10 mol %) in Et_2_O as the solvent. Although the dihydropyridone ring of **17** is hardly puckered, the phenyl substituent is axially oriented to avoid eclipsing with the adjacent *N*‐Ts group (Figure 1).^[29]^ Interestingly, addition of DMF to the crude mixture and raising the temperature from −30 °C to −10 °C entailed the expected ring opening with formation of amide **18 a** in respectable yield and excellent geometrical purity in favor of the non‐thermodynamic *Z,E*‐diene isomer;^[30]^ this stereochemical assignment was confirmed by single crystal X‐ray diffraction (Figure 2). The additional examples compiled in Scheme 5 show that this method has a reasonable scope and provides access to products that are difficult to make otherwise.

**Scheme 6:**
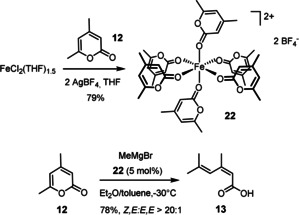
Preparation of the homoleptic iron pyrone complex **22** and examination of its catalytic competence; in the solid state, the unit cell of **22** contains an additional but unbound molecule of **12**.

**Figure 3:**
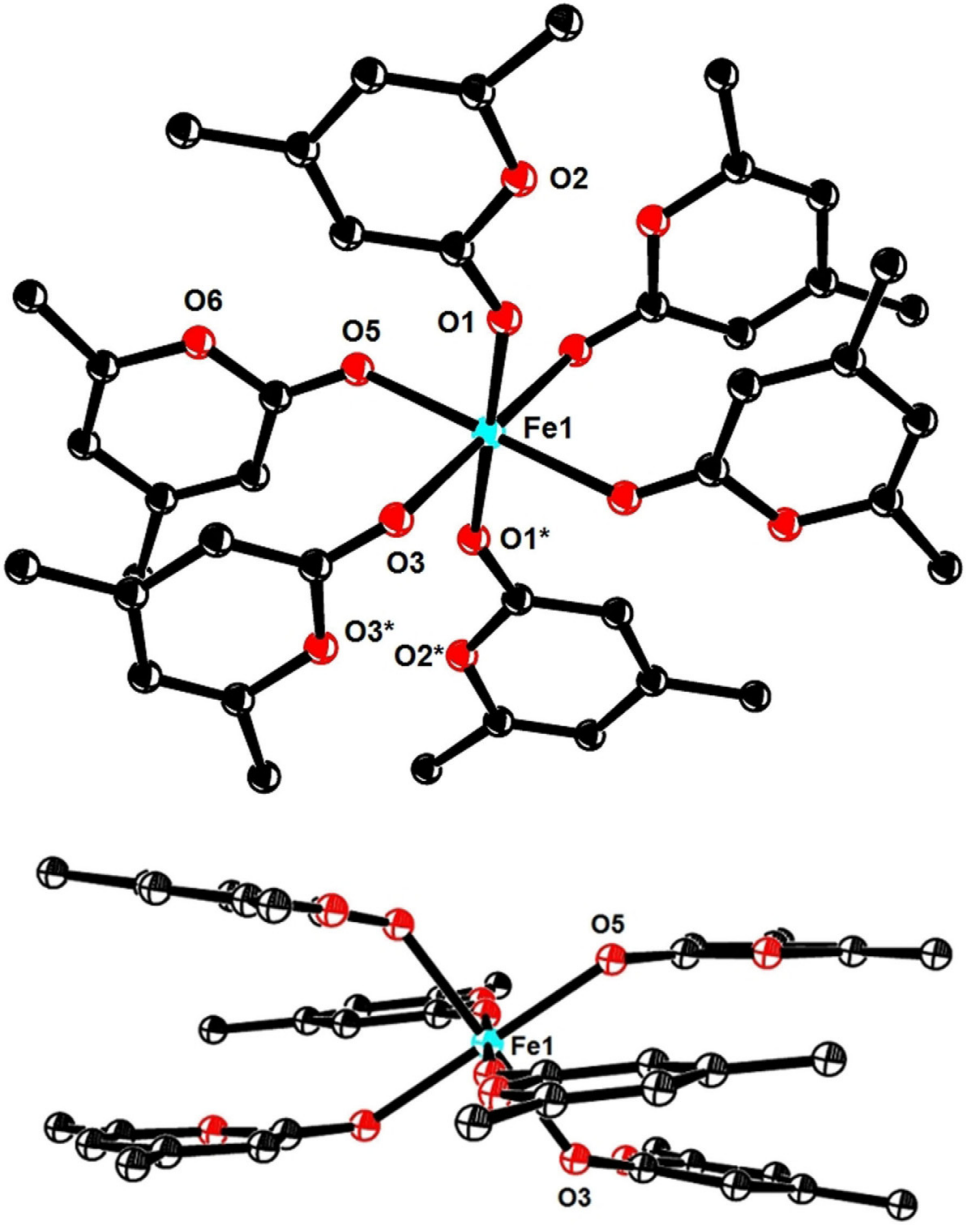
Structure of the Lewis acid/Lewis base adduct **22** in two different orientations; the BF_4_^−^ counterions as well as an additional co‐crystallized but unbound molecule of **12** are not shown for clarity; the full structure is contained in the SI.

The data outlined above leave little doubt that the observed ring opening/cross coupling of 2‐pyrone and 2‐pyridone derivatives is the net outcome of two consecutive steps: of them, only the initial 1,6‐additon is thought to proceed under the aegis of an iron catalyst, whereas the actual ring cleavage is most likely an uncatalyzed event, which is much more facile for 2‐pyrones than for *N*‐tosylated 2‐pyridones.^[30]^ To gain further insights, we studied the interaction of such substrates with various iron sources. The Fe(+2) or Fe(+3) precatalyst is supposed to act as a simple Lewis acid that binds to the more accessible lone pair of the carbonyl group: using an ionized Fe(+2) salt, we managed to obtain a homoleptic adduct that is stable enough to be isolated and catalytically competent (Scheme 6). The structure of complex **22** in the solid state (Figure 3) shows an octahedral environment comprised of six 2‐pyrone ligands about the iron center.^[31]^ The Newman‐type projection along the O1‐Fe1‐O1* axis reveals an interesting organization of the heterocyclic rings via π‐stacking (distance between the centroids of the pyrone rings occupying the equatorial positions, 3.53 Å). All lactone carbonyl groups (for example, O1‐C1 1.233(2) Å) are elongated by coordination to the Lewis‐acidic cation relative to that of the parent pyrone **12** (O1‐C1 1.212(1) Å; the structure of **12** in the solid state is contained in the SI).

**Scheme 7:**
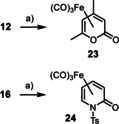
a) Fe(CO)_5_, Bu_2_O/THF (5:1), 65 °C, 19 % (**23**); 21 % (**24**).

**Figure 4:**
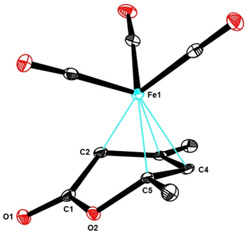
Structure of the pyrone tricarbonyliron complex **23** in the solid state.

**Figure 5:**
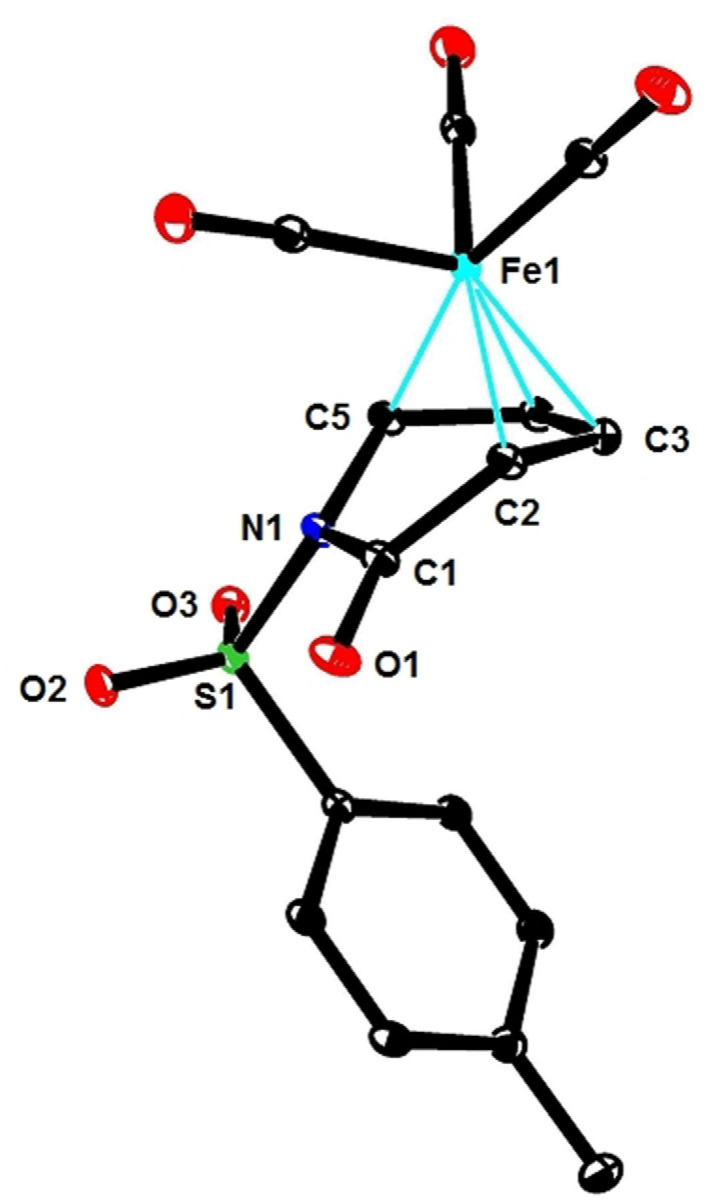
Structure of the pyridone tricarbonyliron complex **24** in the solid state.

Although such coordination to an Fe(+2) center certainly activates the carbonyl group and also enhances the vinylogous Michael acceptor properties of the pyrone, it is unlikely that this canonical Lewis acid/base interaction renders the observed 1,6‐additon so astoundingly facile. Extensive screening in this laboratory showed that iron is almost uniquely capable in catalyzing this transformation.^[32]^ Since iron salts react instantly with polar organometallic reagents to give ate‐complexes[^33^, ^34^, ^35^, ^36^] and/or low valent iron species,[^34^, ^37^, ^38^] the reasons for the unusually facile addition to pyrones and pyridones are likely rooted in a more specific affinity to substrates of this kind. It is, however, exceedingly difficult to establish the nature of the active species generated in situ, not least because low valent organometallic iron complexes are highly sensitive, often paramagnetic, and tend to “age” rapidly; moreover, the exact speciation is strongly dependent on the chosen conditions and the presence/absence of polar additives in the mixture.^[39]^ The mechanistic complexity of the current system is reflected in a number of control experiments, which showed that several well‐defined but structurally quite distinct iron species are competent (pre)catalysts (see Table 1): thus, the intricate Fe(+2)‐ate complex [(Me_4_Fe)⋅(MeLi)][Li⋅(OEt_2_)]_2_ (entry 10),^[33]^ two different Fe^0^ olefin complexes (entries 14, 15),[^37^, ^40^] and even catalytic amounts of Fe(CO)_5_ (entry 11) or the derived pyridone complex (entry 12) worked well. In contrast to the oxophilicity of Fe(+2) and Fe(+3) salts, many low‐valent iron species are inherently carbophilic; 1,3‐dienes are amongst the privileged ligands which get activated toward attack by certain nucleophiles, for example, upon coordination to the [Fe(CO)_3_] fragment.[^41^, ^42^, ^43^] Therefore it seemed likely that the polarized diene motif embedded into the pyrone‐ or pyridone rings constitutes the critical recognition element for the active catalyst. While attempted complexation of **12** or **16** with either [CpFe(C_2_H_4_)_2_][Li(tmeda)][^40^, ^43^] or [dippp)Fe(C_2_H_4_)_2_][^37^, ^43^] failed to afford single crystals suitable for X‐ray diffraction, Fe(CO)_5_ served this purpose well. In this context, it is important to note that (tricarbonyl)iron complexes of 2‐pyrones had previously been found susceptible to nucleophilic attack at the carbonyl group rather than at the enol ether site,[^44^, ^45^] whereas we consider them to be valid models for the reactive intermediate accountable for iron catalyzed 1,6‐addition.^[10]^ Under this premise, 2‐pyridones should be amenable to analogous π‐complexation in order to explain why they react analogously; this is indeed the case (Scheme 7). Figures 4 and 5 illustrate that adduct formation entails massive distortions in both series. Back‐bonding from the low‐valent metal center into the π* orbitals of the η^4^‐bound heterocyclic ligand is manifest in the massive elongation of the C4−C5 bond (1.418(5) Å) in pyrone complex **23** versus 1.340(1) Å in unbound pyrone **12**; the effect is even stronger in the pyridone complex **24** (C4−C5 1.436(2) Å in versus 1.343(2) Å in free **16**). As expected, the C3−C4 bond is somewhat contracted (1.403(5) Å in **23** versus 1.427(1) Å in the **12**; 1.397(2) Å in **24** versus 1.420(3) Å in **16**). Since neither 2‐pyrones nor 2‐pyridones show significant aromatic character, these heterocycles are torsionally flexible. In fact, the lactone (lactam) moiety is forced out of co‐planarity with the olefinic π‐system; as a result, orbital overlap is disrupted and the enol ether (enamide) character lost, at least in part. The incipient carbenoid nature of C5, which carries a carbon‐metal bond and a leaving group, is unmistakable in the structures of complexes **23**, **24** and related complexes[^10^, ^45^] in the solid state. Delivery of an R‐group from a putative loaded iron center to the C5 position becomes not only feasible but likely facile,^[46]^ very much in line with our experimental findings. The concomitant formation of an (iron) enolate enhances the regiochemical course of the reaction and provides an additional driving force; this enolate formation is also “precast” in the structures of **23** or **24**, because the C2−C3 bond of the pyrone (pyridone) ligand has already lost much of its former double bond character (1.435(6) Å in **23** versus 1.358(1) Å in **12**; 1.438(2) in **24** versus 1.351(3) Å in **16**) and a carbon‐metal bond is also looming. Hence, adducts of this type seem to be ideally set up for regioselective transfer of a hydrocarbyl residue from a loaded iron center to the heterocyclic ligand framework.

**Scheme 8:**
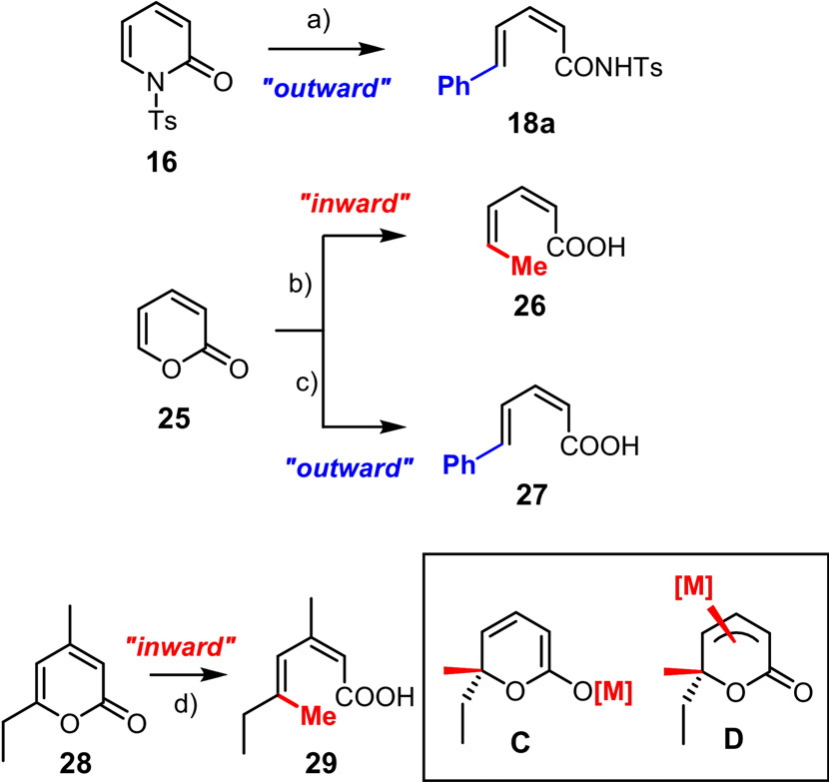
a) PhMgBr, Fe(acac)_3_ (5 mol %), Ph_3_P (10 mol %), Et_2_O, −30 °C, then DMF −30 °C→RT, 60 %; b) MeMgBr, Fe(acac)_3_ (5 mol %), Et_2_O, −30 °C, 86 %; c) PhMgBr, Fe(acac)_3_ (5 mol %), Et_2_O, −30 °C, 44 %; d) MeMgBr, Fe(acac)_3_ (5 mol %), Et_2_O/toluene (1:1), −30 °C, 93 %.

The available experimental evidence is less clear with regard to the subsequent ring opening step. Yet, two pieces of evidence seem to advocate for an ionic rather than electrocyclic mechanism: as outlined above, the addition of DMF is necessary to entail ring cleavage in the pyridone series; pericyclic processes in general, however, are not expected to show such a strong solvent dependence.[^47^, ^48^] A perhaps more stringent argument can be seen in the stereochemical course of the reactions (Scheme 8): the outward rotation of the phenyl groups during the opening of **16** and **25** to give **18 a** and **27**, respectively, would be in line with the torquoselective preference of this substituent, but the exclusive inward orientation of the methyl group in **26** violates the established selectivity rules of electrocyclic processes;^[49]^ the selective formation of **18 a** and **27** is hence more likely thermodynamic in origin (the structure of **27** in the solid state is contained in the SI). Product **29**, the double bond geometry of which mirrors the stereostructure of the substrate, is arguably an even more stringent case:^[5]^ an electrocyclic opening of an intermediate of type **C** would hardly entail formation of a single stereoisomer since the two relevant substituents (Me versus Et) have the same electronic character and are similar in size. Rather, this outcome suggests that some stereochemical communication between the breaking C−O bond and the metal center is operative in the stereodetermining step, as tentatively drawn in **D**. We appreciate, however, that further experimental and computational scrutiny is needed to either confirm or disprove this view.

## Conclusions

The present study extends the structural space covered by iron catalyzed C−C‐bond formation in general and the rather unconventional formal ring opening/cross coupling in particular. Although not all mechanistic questions could be answered in full detail, the available data suggest that the high affinity of low‐valent iron catalysts to (polarized) dienes constitutes a formidable driving force which can likely be exploited in different context too. Further efforts at leveraging this and other as yet untapped opportunities of homogeneous iron catalysis for organic synthesis are underway and will be reported in due course.^[50]^

## Conflict of interest

The authors declare no conflict of interest.

## Supporting information

As a service to our authors and readers, this journal provides supporting information supplied by the authors. Such materials are peer reviewed and may be re‐organized for online delivery, but are not copy‐edited or typeset. Technical support issues arising from supporting information (other than missing files) should be addressed to the authors.

Supplementary
