# Supplementary material for: Iron‐Catalyzed Reactions of 2‐Pyridone Derivatives: 1,6‐Addition and Formal Ring Opening/Cross Coupling
Source: Chem Asian J. 2019 Jul 31;14(22):4017–23. doi: 10.1002/asia.201900865 (PMC7687238; doi:10.1002/asia.201900865)
Supplement: Supplementary file 1 — Supplementary [file ASIA-14-4017-s001.pdf]

CHEMISTRY
AN ASIAN JOURNAL

Supporting Information

Iron-Catalyzed Reactions of 2-Pyridone Derivatives: 1,6-Addition and Formal Ring Opening/Cross Coupling

Lin Huang, Yiting Gu, and Alois Fürstner*[a]

asia_201900865_sm_miscellaneous_information.pdf

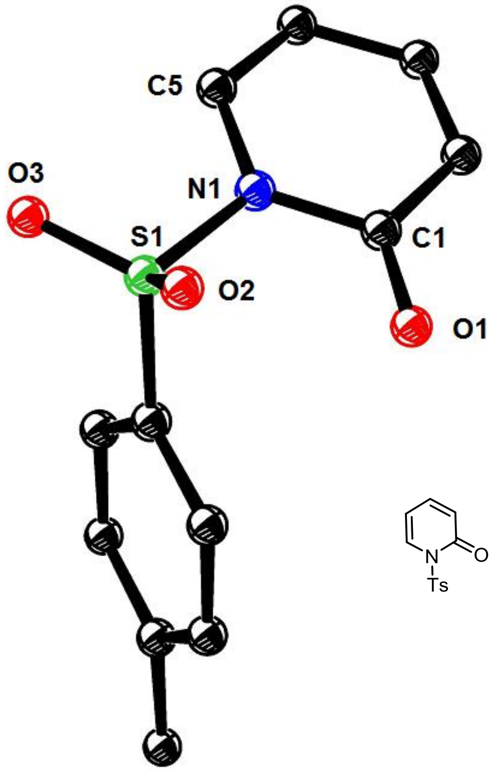

16

Sigure S-1. Structure of pyridone **16** in the solid state; hydrogen atoms are omitted for clarity

X-ray Crystal Structure Analysis of Compound 16: C_{12} H_{11} N O_3 S, *Mr* = 249.28 g · mol^{-1}, colorless prism, crystal size 0.08 x 0.08 x 0.06 mm^3, triclinic, space group $P\bar{1}$, *a* = 6.6271(10) Å, *b* = 7.6892(18) Å, *c* = 11.836(4) Å, α = 87.57(2)°, β = 82.410(17)°, γ = 67.975(17)°, *V* = 554.2(3) $Å^3$, *T* = 100(2) K, *Z* = 2, D_{calc} = 1.494 g · cm^3, λ = 0.71073 Å, μ(*Mo*-*K*α) = 0.287 mm^{-1}, Gaussian absorption correction (T_{min} = 0.98, T_{max} = 0.99), Bruker-AXS Kappa Mach3 APEX-II diffractometer, 3.333 < Θ < 33.047°, 18368 measured reflections, 4182 independent reflections, 2916 reflections with $I > 2\sigma(I)$, R_{int} = 0.0642.

The structure was solved by direct methods and refined by full-matrix least-squares against F^2 to R_1 = 0.053 [$I > 2\sigma(I)$], wR_2 = 0.117, 155 parameters. The H atoms were refined using a riding model, *S* = 1.037, residual electron density 0.6 (0.70 Å from C11)/ –0.4 (0.57 Å from S1) e · $Å^{-3}$. **CCDC- 1917191**.

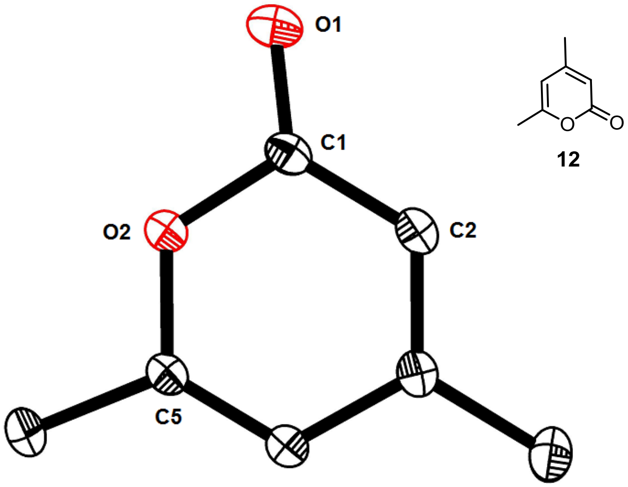

12

Figure S-2. Structure of pyrone **12** in the solid state; hydrogen atoms are omitted for clarity

X-ray Crystal Structure Analysis of Compound 12: $C_7H_8O_2$, *Mr* = 124.13 g · mol^{-1}, colorless block, crystal size 0.32 x 0.14 x 0.12 mm^3, monoclinic, space group $P2_1/c$, *a* = 7.3014(7) Å, *b* = 11.8309(10) Å, *c* = 7.4752(5) Å, β = 91.565(7)°, *V* = 645.48(9) Å^3, *T* = 160(2) K, *Z* = 4, D_{calc} = 1.277 g · cm^3, λ = 0.71073 Å, μ(*Mo-K*α) = 0.093 mm^{-1}, Gaussian absorption correction (T_{min} = 0.98, T_{max} = 0.99), Bruker-AXS Kappa Mach3 APEX-II diffractometer, 3.280 < Θ < 33.035°, 9708 measured reflections, 2417 independent reflections, 2037 reflections with $I > 2\sigma(I)$, R_{int} = 0.0319.

The structure was solved by direct methods and refined by full-matrix least-squares against F^2 to R_1 = 0.046 [$I > 2\sigma(I)$], wR_2 = 0.144, 84 parameters, absolute structure parameter = 0.44(6).

The H atoms were refined using a riding model, *S* = 1.128, residual electron density 0.4 (0.72 Å from C5)/ –0.3 (0.80 Å from C3) e · Å^{-3}. **CCDC- 1917198**.

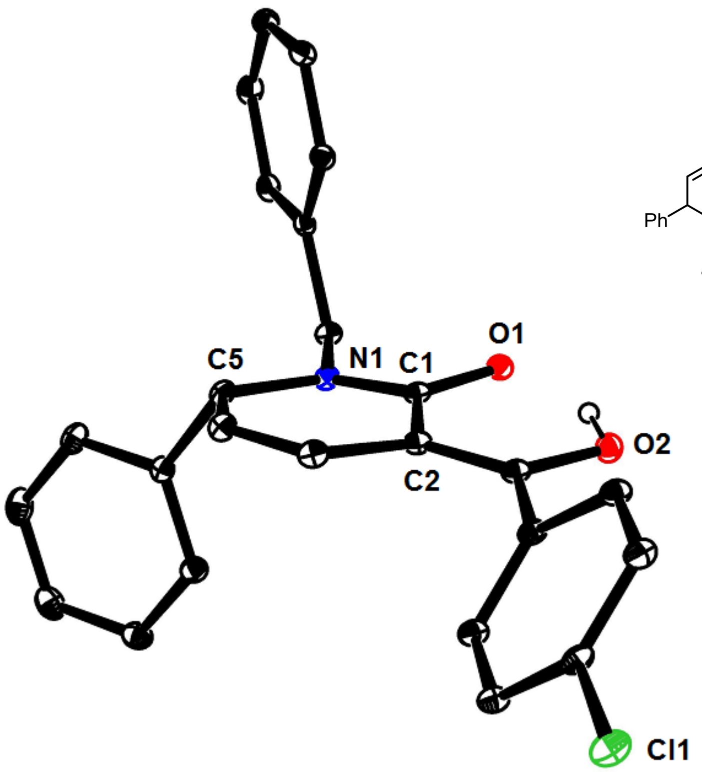

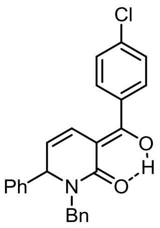

11a

Figure S-3. Structure of compound **11a** in the solid state; all hydrogen atoms except the –OH involved in hydrogen bonding as well as CH_2Cl_2 contained in the unit cell are omitted for clarity

X-ray Crystal Structure Analysis of Compound 11a: $C_{26}H_{22}Cl_3NO_2$, *Mr* = 486.79 g · mol^{-1}, colorless needle, crystal size 0.158 x 0.032 x 0.031 mm^3, monoclinic, space group $P2_1/c$, *a* = 6.8389(11) Å, *b* = 19.279(3) Å, *c* = 17.827(3) Å, β = 100.843(3)°, *V* = 2308.5(7) Å^3, *T* = 100(2) K, *Z* = 4, D_{calc} = 1.401 g · cm^3, λ = 0.71073 Å, μ(*Mo-K*α) = 0.421 mm^{-1}, Gaussian absorption correction (T_{min} = 0.95, T_{max} = 0.99), Bruker-AXS Kappa Mach3 APEX-II diffractometer, 2.326 < Θ < 33.984°, 71776 measured reflections, 9283 independent reflections, 6581 reflections with $I > 2\sigma(I)$, R_{int} = 0.0600.

The structure was solved by direct methods and refined by full-matrix least-squares against F^2 to R_1 = 0.047 [$I > 2\sigma(I)$], wR_2 = 0.129, 293 parameters, absolute structure parameter = 0.44(6).

The H atom at O2 was found and refined, all other H atoms were refined using a riding model, *S* = 1.035, residual electron density 0.7 (0.74 Å from Cl2)/ –0.9 (0.79 Å from Cl2) e · Å^{-3}. **CCDC- 1917196**.

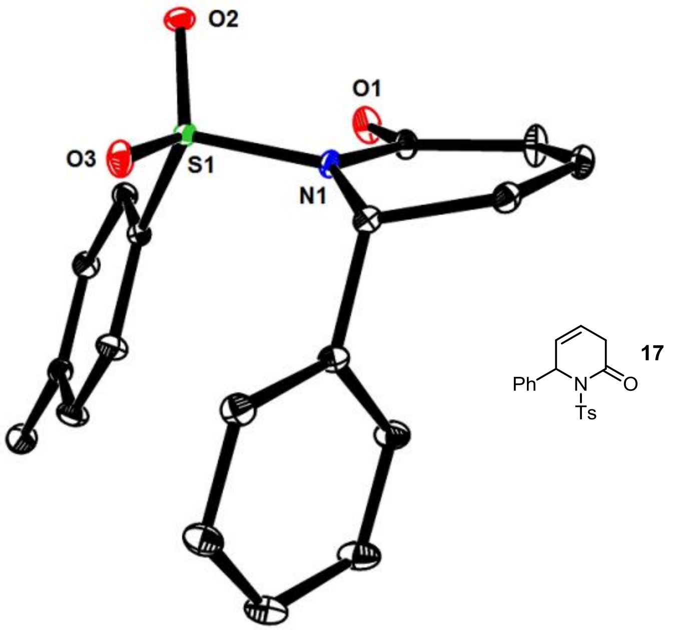

Figure S-4. Structure of compound **17** in the solid state; H-atoms are omitted for clarity

X-ray Crystal Structure Analysis of Compound 17: $C_{18}H_{17}NO_3S$, *Mr* = 327.38 g · mol^{-1}, colorless block, crystal size 0.35 x 0.28 x 0.15 mm^3, orthorhombic, space group $Pna2_1$, a = 11.818(3) Å, b = 16.341(4) Å, c = 8.1630(10) Å, V = 1576.4(5) Å^3, T = 100(2) K, Z = 4, D_{calc} = 1.379 g · cm^3, λ = 0.71073 Å, μ(*Mo-K*α) = 0.220 mm^{-1}, Gaussian absorption correction (T_{min} = 0.94, T_{max} = 0.97), Bruker-AXS Kappa Mach3 APEX-II diffractometer, 2.789 < Θ < 33.010°, 29492 measured reflections, 5883 independent reflections, 5698 reflections with $I > 2\sigma(I)$, R_{int} = 0.0283.

The structure was solved by direct methods and refined by full-matrix least-squares against F^2 to R_1 = 0.026 [$I > 2\sigma(I)$], wR_2 = 0.068, 209 parameters, absolute structure parameter = 0.045(11).

The H atoms were refined using a riding model, S = 1.077, residual electron density 0.3 (0.76 Å from O2)/ –0.2 (0.57 Å from S1) e · Å^{-3}. **CCDC- 1917193**.

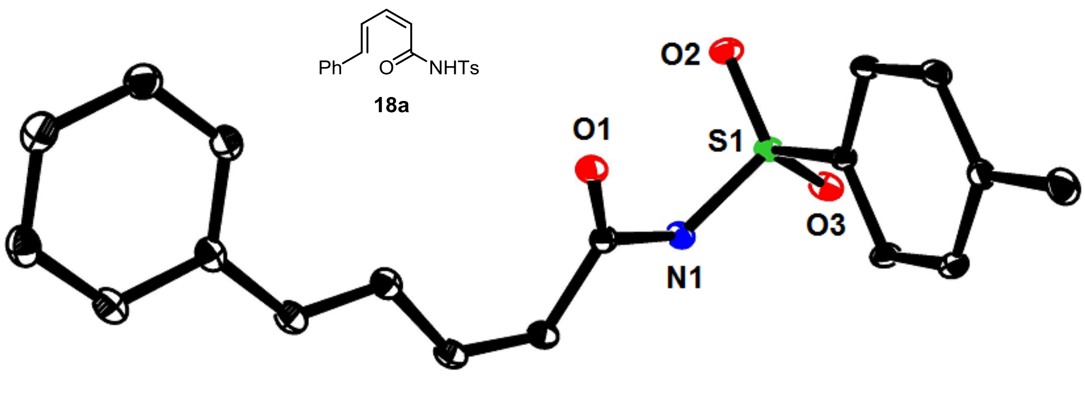

Figure S-5. Structure of compound **18a** in the solid state; H-atoms are omitted for clarity

X-ray Crystal Structure Analysis of Compound 18a: $C_{18}H_{17}NO_3S$, *Mr* = 327.38 g · mol^{-1}, colorless plate, crystal size 0.173 x 0.171 x 0.031 mm^3, monoclinic, space group $P2_1/n$, *a* = 5.9691(7) Å, *b* = 9.9398(11) Å, *c* = 27.354(3) Å, β = 91.067(2)°, *V* = 1622.7(3) Å^3, *T* = 100(2) K, *Z* = 4, D_{calc} = 1.340 g · cm^3, λ = 0.71073 Å, μ(*Mo-K*α) = 0.214 mm^{-1}, Gaussian absorption correction (T_{min} = 0.98, T_{max} = 0.99), Bruker-AXS Kappa Mach3 APEX-II diffractometer, 2.533 < Θ < 31.064°, 47162 measured reflections, 5194 independent reflections, 7804 reflections with $I > 2\sigma(I)$, R_{int} = 0.0310.

The structure was solved by direct methods and refined by full-matrix least-squares against F^2 to R_1 = 0.042 [$I > 2\sigma(I)$], wR_2 = 0.113, 213 parameters. The H atom at N1 was found and refined, all other H atoms were refined using a riding model, *S* = 1.077, residual electron density 0.5 (0.67 Å from C16)/ –0.3 (0.38 Å from S1) e · Å^{-3}. **CCDC- 1917194**.

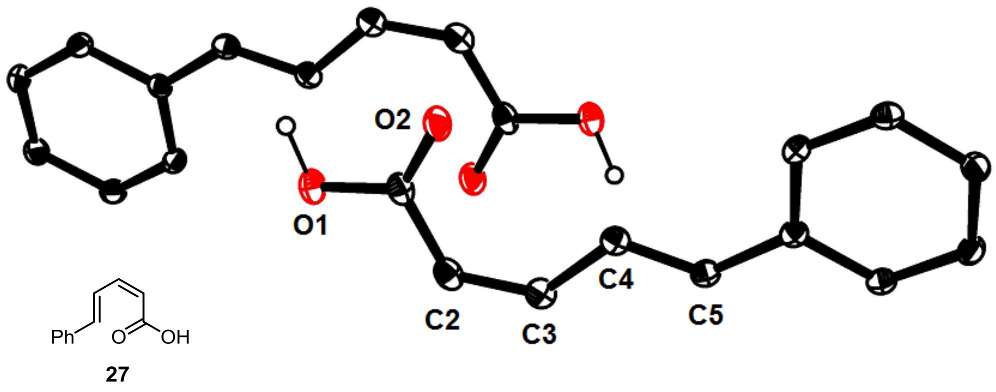

Figure S-6. Structure of dienoic acid **27** in the solid state showing two independent molecules in the unit cell; all but the acidic hydrogen atoms are omitted for clarity

X-ray Crystal Structure Analysis of Compound 27. $C_{11}H_{10}O_2$, *Mr* = 174.19 g · mol^{-1}, colorless needle, crystal size 0.35 x 0.28 x 0.15 mm^3, orthorhombic, space group $Pca2_1$, *a* = 22.5169(14) Å, *b* = 5.5867(4) Å, *c* = 14.4515(9) Å, *V* = 1817.9(2) Å^3, *T* = 100(2) K, *Z* = 8, D_{calc} = 1.273 g · cm^3, λ = 1.54178 Å, μ(*Cu-K*α) = 0.706 mm^{-1}, Gaussian absorption correction (T_{min} = 0.90, T_{max} = 0.97), Bruker-AXS Kappa Mach3 APEX-II diffractometer, 3.926 < Θ < 63.626°, 26900 measured reflections, 2894 independent reflections, 2807 reflections with $I > 2\sigma(I)$, R_{int} = 0.0355.

The structure was solved by direct methods and refined by full-matrix least-squares against F^2 to R_1 = 0.023 [$I > 2\sigma(I)$], wR_2 = 0.058, 244 parameters, absolute structure parameter = –0.10(6), extinction coefficient = 0.0008(2).

The H atoms at O1 and O11 were found and refined, all other H atoms were refined using a riding model, *S* = 1.057, residual electron density 0.1 (0.78 Å from C7)/ –0.1 (0.71 Å from H25) e · Å^{-3}. **CCDC- 1917195**.

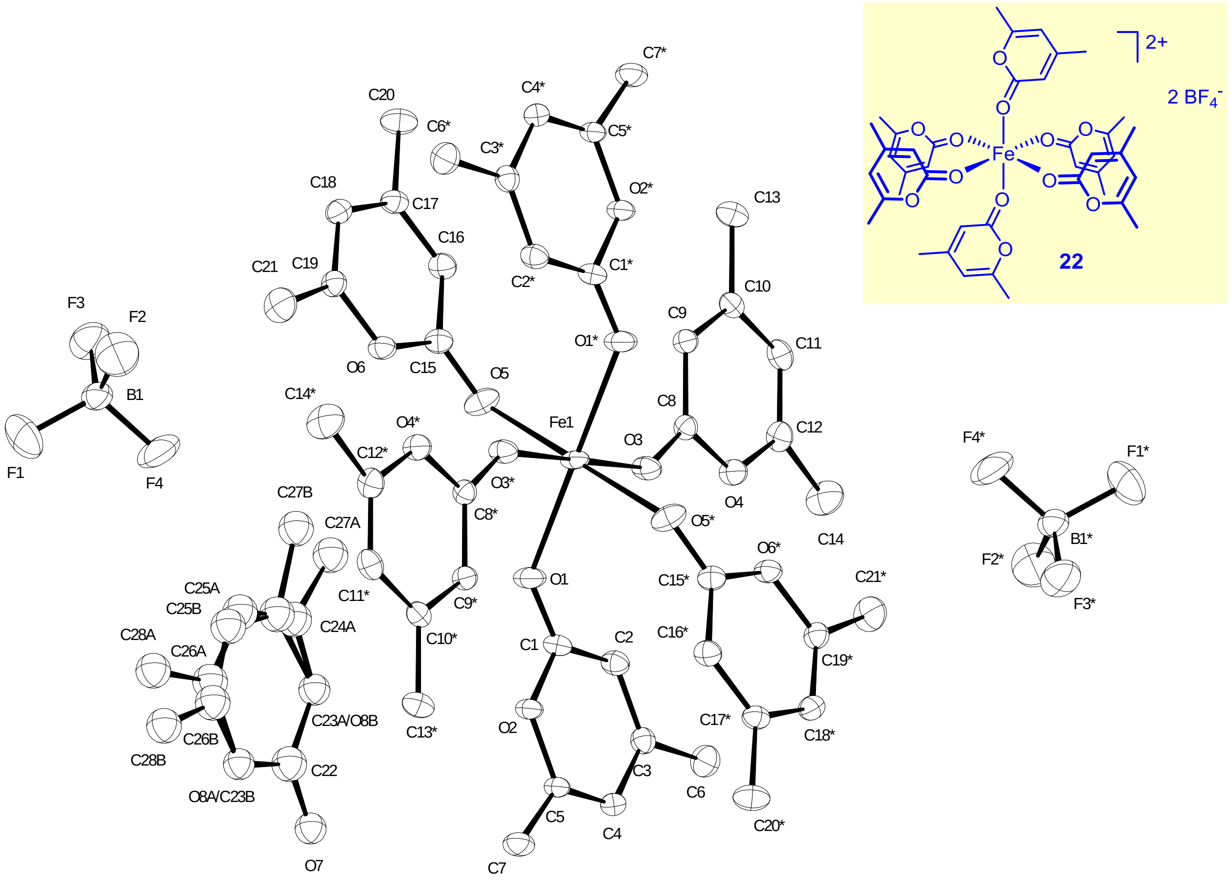

Figure S-7. Structure of the homoleptic iron complex **22** in the solid state; the additional molecule of pyrone 12 contained in the unit cell, which is not coordinated to the iron center, is disordered over two positions

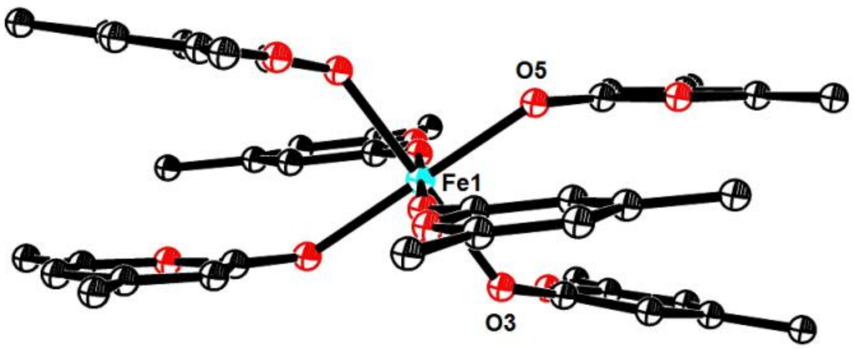

Figure S-8. Structure of the Lewis acid/Lewis base adduct **22** in an orientation that reveals the peculiar organization of the pyrone units; the BF_4^- counterions as well as an additional cocrystallized but unbound molecule of **12** are not shown for clarity

X-ray Crystal Structure Analysis of Complex 22: $C_{56}H_{64}B_2F_8FeO_{16}$, *Mr* = 1222.54 g · mol^{-1}, yellow prism, crystal size 0.165 x 0.083 x 0.050 mm^3, monoclinic, space group $P2_1/n$, *a* = 7.1049(10) Å, *b* = 28.244(4) Å, *c* = 14.292(2) Å, β = 96.654(3)°, *V* = 2848.6(7) Å^3, *T* = 100(2) K, *Z* = 2, D_{calc} = 1.425 g · cm^3, λ = 0.71073 Å, μ(*Mo-K*α) = 0.359 mm^{-1}, Gaussian absorption correction (T_{min} = 0.88, T_{max} = 0.95), Bruker-AXS Kappa Mach3 APEX-II diffractometer, 2.034 < Θ < 33.142°, 82422 measured reflections, 10847 independent reflections, 7804 reflections with $I > 2\sigma(I)$, R_{int} = 0.0847.

The structure was solved by direct methods and refined by full-matrix least-squares against F^2 to R_1 = 0.051 [$I > 2\sigma(I)$], wR_2 = 0.152, 431 parameters. The H atoms were refined using a riding model, *S* = 0.959, residual electron density 0.8 (0.89 Å from Fe1)/ –0.9 (0.55 Å from Fe1) e · Å^{-3}. **CCDC- 1917197**.

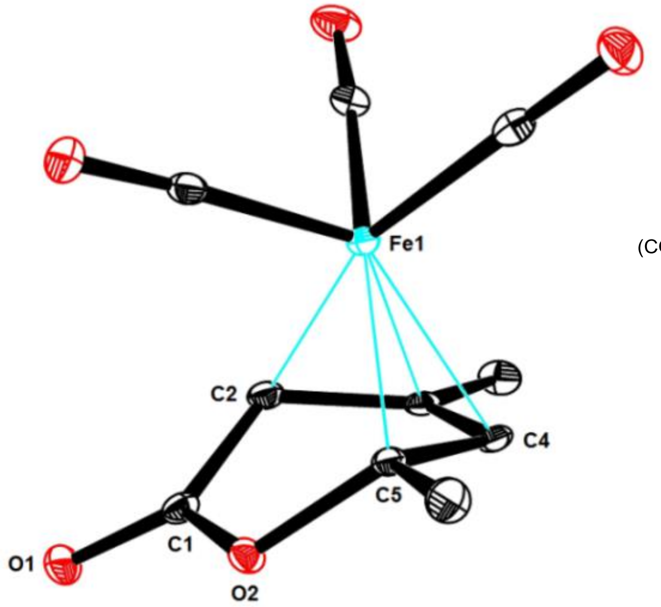

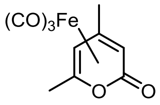

23

Figure S-9. Structure of the pyrone tricarbonyliron complex **23** in the solid state; hydrogen atoms are omitted for clarity

X-ray Crystal Structure Analysis of Complex 23: $C_{10}H_8FeO_5$, *Mr* = 519.9(2) g · mol^{-1}, yellow plate, crystal size 0.07 x 0.05 x 0.05 mm^3, monoclinic, space group $P2_1$, *a* = 6.9641(14) Å, *b* = 10.957(2) Å, *c* = 7.5654(15) Å, β = 115.76(3)°, *V* = 1622.7(3) Å^3, *T* = 100(2) K, *Z* = 2, D_{calc} = 1.686 g · cm^3, λ = 0.71073 Å, μ(*Mo-K*α) = 1.451 mm^{-1}, Gaussian absorption correction (T_{min} = 0.98, T_{max} = 0.99), Bruker-AXS Kappa Mach3 APEX-II diffractometer, 2.990 < Θ < 30.079°, 1586 measured reflections, 1586 independent reflections, 1389 reflections with $I > 2\sigma(I)$, R_{int} = 0.

The structure was solved by direct methods and refined by full-matrix least-squares against F^2 to R_1 = 0.040 [$I > 2\sigma(I)$], wR_2 = 0.068, 148 parameters, absolute structure parameter = 0.44(6).

The H atoms were refined using a riding model, *S* = 1.143, residual electron density 0.4 (0.82 Å from C9)/ –0.6 (0.78 Å from Fe1) e · Å^{-3}. **CCDC- 1917190**.

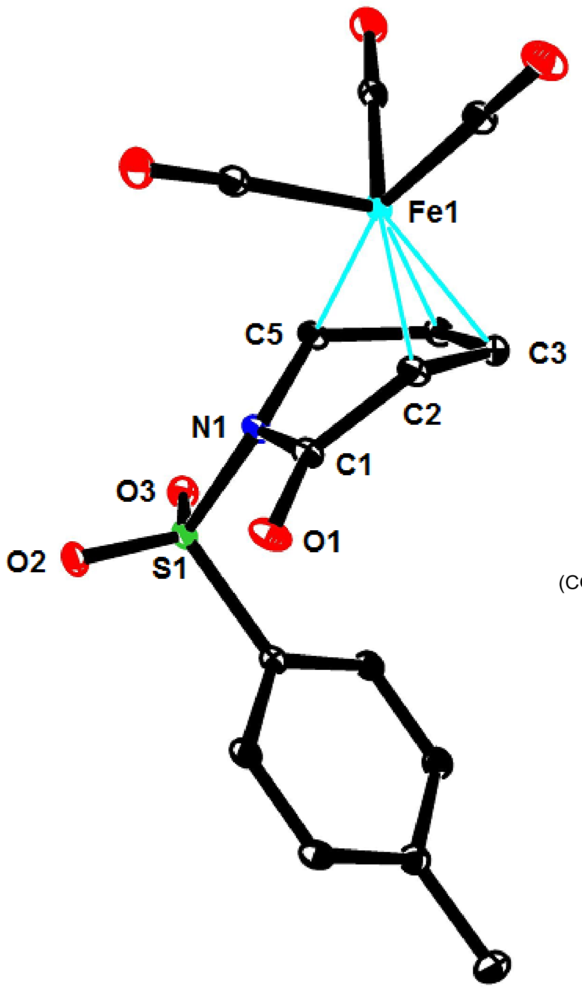

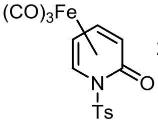

24

Figure S-10. Structure of the pyridone tricarbonyliron complex **24** in the solid state; hydrogen atoms are omitted for clarity

X-ray Crystal Structure Analysis of Complex 24: $C_{15}H_{11}FeNO_6S$, *Mr* = 389.16 g · mol^{-1}, yellow needle, crystal size 0.40 x 0.36 x 0.19 mm^3, triclinic, space group *P*1, *a* = 6.3777(5) Å, *b* = 6.9416(3) Å, *c* = 18.0314(19) Å, α = 96.474(7)°, β = 93.427(8)°, γ = 107.045(5)°, *V* = 754.72(11) Å^3, *T* = 100(2) K, *Z* = 2, D_{calc} = 1.712 g · cm^3, λ = 0.71073 Å, μ(*Mo-K*α) = 1.170 mm^{-1}, Gaussian absorption correction (T_{min} = 0.86, T_{max} = 0.97), Bruker-AXS Kappa Mach3 APEX-II diffractometer, 3.098 < Θ < 33.036°, 14110 measured reflections, 5672 independent reflections, 4717 reflections with $I > 2\sigma(I)$, R_{int} = 0.0387.

The structure was solved by direct methods and refined by full-matrix least-squares against F^2 to R_1 = 0.040 [$I > 2\sigma(I)$], wR_2 = 0.099, 218 parameters. The H atoms were refined using a riding model, *S* = 1.058, residual electron density 0.6 (0.80 Å from Fe1)/ –1.0 (0.72 Å from Fe1) e · Å^{-3}. **CCDC- 1917192**.

General. Unless stated otherwise, all reactions were carried out in flame-dried glassware using anhydrous solvents under argon. The solvents were purified by distillation over the following drying agents and were transferred under argon: THF, Et_2O, *n*-Bu_2O, DME (Mg/anthracene), CH_2Cl_2, toluene (Na/K); DMF, Et_3N and pyridine were dried by an adsorption solvent purification system based on molecular sieves. Thin layer chromatography (TLC): Macherey-Nagel precoated plates (POLYGRAM®SIL/UV254). Flash chromatography: Merck silica gel 60 (40–63 µm) or Macherey-Nagel fine silica gel 60 (15-40 µm) with predistilled or HPLC grade solvents; NMR: Spectra were recorded on Bruker DPX 300 or AV 400 spectrometers in the solvents indicated; chemical shifts (δ) are given in ppm relative to TMS, coupling constants (*J*) in Hz. The solvent signals were used as references and the chemical shifts converted to the TMS scale ($CDCl_3$: δ_C = 77.16 ppm; residual $CHCl_3$ in $CDCl_3$: δ_H = 7.26 ppm). IR: Spectrum One (Perkin-Elmer) spectrometer, wavenumbers ($\tilde{\nu}$) in cm^{-1}. MS (EI): Finnigan MAT 8200 (70 eV), ESI-MS: ESQ3000 (Bruker), accurate mass determinations: Bruker APEX III FTMS (7 T magnet) or Mat 95 (Finnigan).

Unless stated otherwise, all commercially available compounds (ABCR, Acros, Alfa Aesar, Aldrich, TCI, Strem Chemicals) were used as received.

Substrates

Representative Procedure for the Synthesis of 1-Substituted Pyridin-2(1*H*)-ones. 1-Benzylpyridin-2(1*H*)-one (**1**).

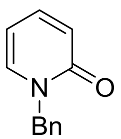

A mixture of 2-pyridin-2(1*H*)-one (2.00 g, 21 mmol), benzyl bromide (5.14 g, 30 mmol) and K_2CO_3 (5.80 g mg, 47 mmol) in DME (40 mL) was stirred for 16 h at 70 °C. After reaching ambient temperature, the reaction was quenched with water and the aqueous layer was extracted with EtOAc (3 x 20 mL). The combined organic layers were washed with brine, dried over $MgSO_4$, filtered and evaporated. The residue was purified by flash chromatography (pentane/EtOAc, 1/1 to 1/7) to afford the title compound as a white solid (3.14 g, 96%). m.p. = 74.0-74.4 °C. ^{1}H NMR (400 MHz, $CDCl_3$) δ 7.43 – 7.20 (m, 7H), 6.61 (d, *J* = 9.2 Hz, 1H), 6.13 (td, *J* = 6.8, 1.4 Hz, 1H), 5.15 (s, 2H). ^{13}C NMR (101 MHz, $CDCl_3$) δ 162.8, 139.5, 137.3, 136.5, 129.0, 128.3, 128.1, 121.4, 106.3, 52.0. IR (film, cm^{-1}): 3061, 3033, 2928, 1655, 1575, 1422, 1168, 1022, 949, 867, 727, 560. HRMS (EI): *m/z*: calcd for $C_{12}H_{11}NO$ [M^+]: 185.08351, found: 185.08364.

The following compounds were prepared analogously:

Compound S1. Using MeI instead of BnBr as the alkylating agent; colorless oil (1.27 g, 86%). ^{1}H NMR (400 MHz, $CDCl_3$) δ 7.32 (ddd, *J* = 9.0, 6.6, 2.1 Hz, 1H), 7.29 – 7.26 (m, 1H), 6.57 (d, *J* = 9.0 Hz, 1H), 6.14 (td, *J* = 6.6, 1.4 Hz, 1H), 3.54 (s, 3H). ^{13}C NMR (101 MHz, $CDCl_3$) δ 163.3, 139.6, 138.4, 120.9, 106.0, 37.8. IR (film, cm^{-1}): 3080, 3031, 2916, 1650, 1575, 1537, 1412, 1317, 1153, 1051, 875, 843, 761, 728, 528, 459. HRMS (EI): *m/z*: calcd for C_6H_7NO [M^+]: 109.05221, found: 109.05228.

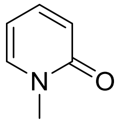

Compound S2. White solid (1.00 g, 98%); m.p. = 85.5-86.4 °C. ^{1}H NMR (400 MHz, $CDCl_3$) δ 7.35 – 7.23 (m, 4H), 7.06 – 6.98 (m, 2H), 6.61 (d, *J* = 8.8 Hz, 1H), 6.15 (td, *J* = 6.7, 1.3 Hz, 1H), 5.10 (s, 2H). ^{13}C NMR (101 MHz, $CDCl_3$) δ 162.9, 162.6 (d, *J* = 246.7 Hz), 139.6, 137.2, 132.4 (d, *J* = 3.4 Hz), 130.1 (d, *J* = 8.4 Hz), 121.6, 115.9 (d, *J* = 21.6 Hz), 106.5, 51.5. ^{19}F NMR (282 MHz, $CDCl_3$) δ –114.1. IR (film, cm^{-1}): 3086, 3066, 3029, 2997, 2957, 1652, 1579, 1505, 1434, 1351, 1221, 1147, 1086, 940, 831, 757, 573, 472. HRMS (EI): *m*/*z*: calcd for $C_{12}H_{10}NOF$ [M^+]: 203.07409, found: 203.07448.

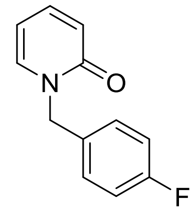

Compound S3. White solid (1.04 g, 97%); m.p. = 61.4-62.2 °C. ^{1}H NMR (400 MHz, $CDCl_3$) δ 7.31 (ddd, *J* = 9.0, 6.6, 2.0 Hz, 1H), 7.28 – 7.23 (m, 2H), 6.92 – 6.80 (m, 3H), 6.61 (ddd, *J* = 9.0, 1.4, 0.8 Hz, 1H), 6.14 (td, *J* = 6.7, 1.4 Hz, 1H), 5.12 (s, 2H), 3.78 (s, 3H). ^{13}C NMR (101 MHz, $CDCl_3$) δ 162.8, 160.1, 139.5, 138.0, 137.3, 130.1, 121.4, 120.5, 113.9, 113.6, 106.3, 55.4, 51.9. IR (film, cm^{-1}): 3070, 3054, 3029, 3001, 2970, 2929, 2843, 1652, 1584, 1538, 1490, 1439, 1422, 1341, 1287, 1261, 1136, 1048, 871, 845, 765, 691, 571, 522. HRMS (EI): *m*/*z*: calcd for $C_{13}H_{13}NO_2$ [M^+]: 215.09408, found: 215.09427.

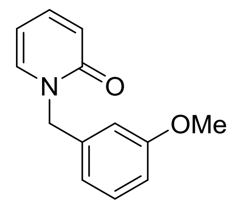

Compound S4. White solid (7.21 g, 95%); m.p. = 90.2-90.8 °C. ^{1}H NMR (400 MHz, $CDCl_3$) δ 7.41 – 7.27 (m, 7H), 6.53 (dd, *J* = 9.7, 0.6 Hz, 1H), 5.10 (s, 2H). ^{13}C NMR (101 MHz, $CDCl_3$) δ 161.2, 142.6, 137.1, 135.8, 129.2, 128.5, 128.4, 122.6, 98.2, 52.2. IR (film, cm^{-1}): 3064, 3043, 2995, 1654, 1579, 1525, 1492, 1433, 1349, 1241, 1150, 1067, 826, 742, 692, 642, 573, 514. HRMS (EI): *m*/*z*: calcd for $C_{12}H_{10}NOBr$ [M^+]: 262.99404, found: 262.99423.

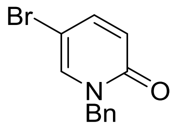

1-Benzyl-5-methylpyridin-2(1*H*)-one (S5).[1] Under Ar atmosphere, a mixture of 1-benzyl-5-bromopyridin-2(1*H*)-one **S4** (792 mg, 3.0 mmol), potassium methyltrifluoroborate (476 mg, 3.9 mmol), $Pd(OAc)_2$ (33.7 mg, 0.15 mmol), RuPhos (140 mg, 0.30 mmol, 10 mol%) and K_2CO_3 (1.24 g, 9 mmol) in toluene/H_2O (*v/v* = 4/1, 5 mL) was stirred for 5 h at 90 °C (bath temperature) until the reaction was completed. The mixture was cooled to ambient temperature before the reaction was quenched with water and the aqueous phase was extracted with EtOAc (3 x 20 mL). The combined organic layers were washed with brine, dried over $MgSO_4$, filtered and evaporated. The residue was purified by flash chromatography (pentane/EtOAc, 2/1 to 1/2) to afford the title compound as a white solid (420 mg, 70%). m.p. = 76.2-78.1 °C. ^{1}H NMR (400 MHz, $CDCl_3$) δ 7.37 – 7.26 (m, 5H), 7.17 (dd, *J* = 9.3, 2.5 Hz, 1H), 7.02 (s, 1H), 6.56 (d, *J* = 9.3 Hz, 1H), 5.11 (s, 2H), 2.02 (s, 3H). ^{13}C NMR (101 MHz, $CDCl_3$) δ 162.1, 142.2, 136.8, 134.7, 129.0, 128.2, 128.0, 121.0, 115.4, 51.8, 17.3. IR (film, cm^{-1}): 3035, 2952, 2925, 1664, 1584, 1537, 1495, 1430, 1265, 1144, 1073, 916, 828, 715, 697, 522, 481. HRMS (EI): *m*/*z*: calcd for $C_{13}H_{13}NO$ [M^+]: 199.09916, found: 199.09931.

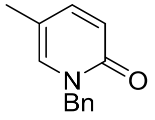

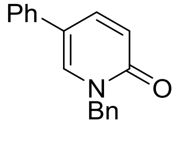

Compound S6. Prepared analogously using potassium phenyltrifluoroborate as the reagent; white solid (684 mg, 87%); m.p. = 73.6-75.2 °C. ^{1}H NMR (400 MHz, $CDCl_3$) δ 7.64 (dd, *J* = 8.8, 2.8 Hz, 1H), 7.51 (d, *J* = 2.3 Hz, 1H), 7.41 – 7.29 (m, 10H), 6.73 (d, *J* = 9.2 Hz,

1H), 5.23 (s, 2H). ^{13}C NMR (101 MHz, $CDCl_3$) δ 162.4, 139.4, 136.5, 136.4, 134.7, 134.1, 129.2, 129.1, 128.3, 127.5, 125.9, 121.3, 120.7, 52.4. IR (film, cm^{-1}): 3054, 3029, 2947, 1656, 1584, 1532, 1495, 1423, 1365, 1299, 1151, 1076, 893, 833, 764, 694, 597, 490. HRMS (EI): *m/z*: calcd for $C_{18}H_{15}NO$ [M^+]: 261.11481, found: 261.11516.

Compound S7. Prepared analogously using potassium cyclopropyltrifluoroborate as the reagent; pale

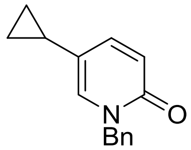

yellow oil (651 mg, 97%); ^{1}H NMR (400 MHz, $CDCl_3$) δ 7.37 – 7.26 (m, 5H), 7.09 (dd, *J* = 9.3, 2.6 Hz, 1H), 7.05 (d, *J* = 2.6 Hz, 1H), 6.56 (d, *J* = 9.3 Hz, 1H), 5.12 (s, 2H), 1.61 (tt, *J* = 8.4, 5.1 Hz, 1H), 0.80 (ddd, *J* = 8.4, 6.2, 4.8 Hz, 2H), 0.47 (dt, *J* = 6.2, 4.8 Hz, 2H). ^{13}C NMR (101 MHz, $CDCl_3$) δ 162.2, 139.3, 136.7, 134.1, 129.0, 128.2, 128.0, 121.4, 121.1, 52.0, 12.0, 6.7. IR (film, cm^{-1}): 3080, 3065, 3031, 3004, 2930, 2851, 1662, 1592, 1535, 1454, 1368, 1260, 1155, 878, 830, 731, 699, 559. HRMS (EI): *m/z*: calcd for $C_{15}H_{15}NO$ [M^+]: 225.11481, found: 225.11500.

1-Phenyl-pyridin-2(1*H*)-one (S8).[2] A mixture of 2-pyridone (1.00 g, 10.5 mmol), CuI (0.2 g, 1.06 mmol, 10

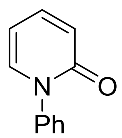

mol%), iodobenzene (2.35 mL, 21 mmol) and K_2CO_3 (1.47 g, 10.5 mmol) in DMF (2 mL) was stirred for 12 h at 150 °C under Ar. The mixture was allowed to cool before the reaction was quenched with water and the aqueous phase extracted with EtOAc (3 x 20 mL). The combined organic layers were washed with brine, dried over $MgSO_4$, filtered and evaporated, and the residue was purified by flash chromatography to afford the title compound as a white solid (1.52 g, 85%). m.p. = 128.0-129.3 °C. ^{1}H NMR (400 MHz, $CDCl_3$) δ 7.52 – 7.46 (m, 2H), 7.45 – 7.36 (m, 4H), 7.33 (ddd, *J* = 6.9, 2.1, 0.6 Hz, 1H), 6.66 (d, *J* = 9.3 Hz, 1H), 6.23 (td, *J* = 6.7, 1.3 Hz, 1H). ^{13}C NMR (101 MHz, $CDCl_3$) δ 162.5, 141.1, 139.9, 138.1, 129.5, 128.6, 126.7, 122.1, 106.0. IR (film, cm^{-1}): 3052, 3041, 3014, 2913, 1656, 1578, 1526, 1488, 1272, 1252, 1126, 983, 839, 756, 692, 582, 453. HRMS (EI): *m/z*: calcd for $C_{11}H_9NO$ [M^+]: 171.06786, found: 171.06795.

1-Tosyl-pyridin-2(1*H*)-one (16). A mixture of 2-pyridone (2.00 g, 21 mmol), tosyl chloride (4.41 g, 23

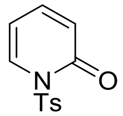

mmol) and Et_3N (4.26 g, 42 mmol) in THF (20 mL) was stirred at ambient temperature for 12 h. The reaction was quenched with water and the aqueous phase extracted with EtOAc (3 x 20 mL). The combined organic layers were washed with brine, dried over $MgSO_4$, filtered and evaporated. The residue was purified by flash chromatography (pentane/EtOAc, 4/1 to 1/1) to afford the title compound as a white solid (1.15 g, 21%). m.p. = 141.3-142.0 °C. ^{1}H NMR (400 MHz, $CDCl_3$) δ 8.09 (ddd, *J* = 7.6, 2.0, 0.8 Hz, 1H), 8.02 – 7.96 (m, 2H), 7.38 – 7.27 (m, 3H), 6.41 (dt, *J* = 9.3, 1.2 Hz, 1H), 6.24 (ddd, *J* = 7.6, 6.4, 1.2 Hz, 1H), 2.43 (s, 3H). ^{13}C NMR (101 MHz, $CDCl_3$) δ 160.3, 146.4, 141.3, 133.4, 131.8, 130.1, 129.6, 123.6, 106.4, 22.0. IR (film, cm^{-1}): 3354, 3260, 3121, 3106, 1673, 1601, 1530, 1359, 1245, 1163, 1123, 1086, 1020, 819, 770, 686, 646, 544, 512. HRMS (ESI): *m/z*: calcd for $C_{12}H_{11}NO_3SNa$ [$M+Na^+$]: 272.03519, found: 272.03505.

Compound S9. Prepared analogously using potassium *p*-methoxyphenylsulfonyl chloride as the reagent;

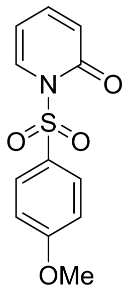

white solid (1.05 g, 19%); m.p. = 120.4-122.3 °C. ^{1}H NMR (400 MHz, $CDCl_3$) δ 8.14 – 8.01 (m, 3H), 7.30 (ddd, *J* = 9.3, 6.4, 2.0 Hz, 1H), 7.00 (dt, *J* = 9.2, 2.0 Hz, 2H), 6.41 (dt, *J* = 9.4, 1.0 Hz, 1H), 6.23 (ddd, *J* = 7.6, 6.4, 1.3 Hz, 1H), 3.88 (s, 3H). ^{13}C NMR (101 MHz, $CDCl_3$) δ 164.8, 160.3, 141.2, 132.6, 131.8, 127.4, 123.6, 114.2, 106.2, 55.9. IR (film, cm^{-1}): 3118, 3103, 3023, 2988, 2954, 2851, 1673, 1592, 1536, 1497, 1359, 1271, 1251, 1167, 1127, 1089, 1016, 829, 801, 765, 684, 569, 550. HRMS (ESI): *m/z*: calcd for $C_{12}H_{12}NO_4S$ [$M+H^+$]: 266.04816, found:266.04823.

Compound S10. Prepared analogously using potassium *p*-fluorophenylsulfonyl chloride as the reagent;

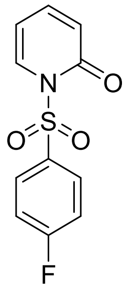

white solid (327 mg, 45%); m.p. = 172.0-173.1 °C. ^{1}H NMR (400 MHz, $CDCl_3$) δ 8.22 – 8.11 (m, 2H), 8.07 (dd, *J* = 7.6, 2.0 Hz, 1H), 7.32 (ddd, *J* = 9.4, 6.4, 2.0 Hz, 1H), 7.26 – 7.18 (m, 2H), 6.43 (dt, *J* = 9.4, 1.0 Hz, 1H), 6.26 (ddd, *J* = 7.6, 6.4, 1.3 Hz, 1H). ^{13}C NMR (101 MHz, $CDCl_3$) δ 166.5 (d, *J* = 258.6 Hz), 160.2, 141.5, 133.2 (d, *J* = 10.1 Hz), 132.3 (d, *J* = 3.1 Hz), 131.6, 123.6, 116.4 (d, *J* = 23.1 Hz), 106.6. ^{19}F NMR (282 MHz, $CDCl_3$) δ –100.9. IR (film, cm^{-1}): 3116, 3097, 3065, 3042, 1675, 1583, 1534, 1488, 1362, 1233, 1181, 1127, 1084, 1009, 819, 759, 692, 543, 503. HRMS (ESI): *m/z*: calcd for $C_{11}H_8FNO_3SNa$ [$M+Na^+$]: 276.01011, found: 26.01000.

Compound S11. Prepared analogously using potassium thiophene-*2*-sulfonyl chloride as the reagent;

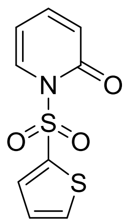

white solid (2.49 g, 62%); m.p. = 134.1-134.9 °C. ^{1}H NMR (400 MHz, $CDCl_3$) δ 8.07 (dd, *J* = 3.9, 1.5 Hz, 1H), 8.02 (ddd, *J* = 7.6, 2.0, 0.8 Hz, 1H), 7.78 (dd, *J* = 5.0, 1.4 Hz, 1H), 7.32 (ddd, *J* = 9.4, 6.4, 1.9 Hz, 1H), 7.14 (dd, *J* = 5.0, 3.9 Hz, 1H), 6.48 (dt, *J* = 9.4, 1.0 Hz, 1H), 6.24 (ddd, *J* = 7.6, 6.4, 1.3 Hz, 1H). ^{13}C NMR (101 MHz, $CDCl_3$) δ 160.3, 141.3, 138.0, 136.5, 135.7, 131.5, 127.5, 123.5, 106.6. IR (film, cm^{-1}): 3110, 3097, 1679, 1600, 1530, 1376, 1340, 1246, 1227, 1170, 1128, 1011, 824, 747, 682, 562, 511. HRMS (ESI): *m/z*: calcd for $C_9H_7NO_3SNa$ [$M+Na^+$]: 263.97596, found: 263.97607.

Iron Catalyzed Reactions

Representative Procedure for the Iron Catalyzed Pyridone 1,6-Addition Reaction. 1-Benzyl-6-phenyl-3,6-dihydropyridin-2(1*H*)-one (2a).

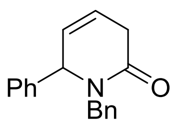

A solution of PhMgBr (3 M in THF, 0.15 mL, 0.45 mmol) was added drowpise to a rapidly stirred solution of compound **1** (55.6 mg, 0.3 mmol) and $Fe(acac)_3$ (5.3 mg, 0.015 mmol) in THF (3 mL) at –45 °C. The mixture was stirred for 50 min at this temperature before the reaction was quenched with sat. aq. NH_4Cl. The aqueous phase was extracted with EtOAc (3 x 10 mL), the combined organic layers were washed with brine, dried over $MgSO_4$, filtered and evaporated, and the residue was purified by flash chromatography (pentane/EtOAc = 10/1, 6/1, 4/1, 2/1) to afford the title compound as a pale yellow solid (76.1 mg, 96%). m.p. = 117.2-118.4 °C. ^{1}H NMR (400 MHz, $CDCl_3$) δ 7.41 – 7.26 (m, 6H), 7.22 – 7.15 (m, 4H), 5.81 – 5.72 (m, 1H), 5.72 – 5.65 (m, 1H), 5.61 (d, *J* = 14.9 Hz, 1H), 4.81 (qd, *J* = 3.9, 1.2 Hz, 1H),

3.42 (d, *J* = 14.9 Hz, 1H), 3.33 – 3.14 (m, 2H). ^{13}C NMR (101 MHz, $CDCl_3$) δ 167.7, 140.2, 136.9, 129.2, 128.8, 128.4, 128.3, 127.6, 127.2, 126.5, 120.6, 61.8, 46.4, 32.3. IR (film, cm^{-1}): 3086, 3057, 3022, 3006, 2925, 2891, 1638, 1449, 1402, 1316, 1262, 1147, 1027, 940, 841, 767, 700, 481. HRMS (EI): *m/z*: calcd for $C_{18}H_{17}NO$ [M^+]: 263.13046, found: 263.13065.

The following compounds were prepared analogously on 0.2 mmol scale:

Compound 2b. The reaction was performed using 3 equiv of ArMgBr; pale yellow solid (36.8 mg, 72%).

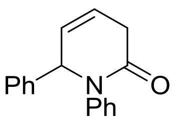

m.p. = 95.4-96.9 °C. ^1H NMR (400 MHz, $CDCl_3$) δ 7.28 – 7.15 (m, 6H), 7.10 – 7.06 (m, 2H), 6.97 – 6.91 (m, 2H), 5.95 – 5.85 (m, 2H), 5.26 (q, *J* = 3.3 Hz, 1H), 3.42 – 3.32 (m, 1H), 3.30 – 3.21 (m, 1H). ^{13}C NMR (101 MHz, $CDCl_3$) δ 167.4, 141.1, 140.0, 129.1, 128.8, 128.2, 128.1, 127.6, 127.4, 126.4, 121.2, 67.2, 32.8. IR (film, cm^{-1}): 3083, 3059, 3030, 3006, 2931, 2880, 1643, 1431, 1403, 1288, 1141, 1074, 759, 693, 559. HRMS (EI): *m/z*: calcd for $C_{17}H_{15}NO$ [M^+]: 249.11481, found: 249.11503.

Compound 2c. The reaction was performed using 3 equiv of ArMgBr at –20 °C; white solid (17.9 mg, 46%).

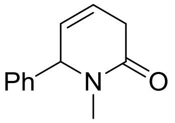

m.p. = 63.3-64.6 °C. ^1H NMR (400 MHz, $CDCl_3$) δ 7.40 – 7.27 (m, 3H), 7.23 – 7.16 (m, 2H), 5.80 – 5.67 (m, 2H), 4.87 (dd, *J* = 3.3 Hz, 1H), 3.21 – 3.03 (m, 2H), 2.82 (s, 3H). ^{13}C NMR (101 MHz, $CDCl_3$) δ 167.7, 140.3, 129.2, 128.3, 126.9, 126.2, 120.9, 65.7, 32.8, 32.0. IR (film, cm^{-1}): 3082, 3055, 3026, 2907, 2797, 1630, 1487, 1448, 1398, 1311, 1242, 1060, 836, 759, 695, 437. HRMS (EI): *m/z*: calcd for $C_{12}H_{13}NO$ [M^+]: 187.09916, found: 187.09931.

Compound 3a. The reaction was performed using 3 equiv of ArMgBr at -20 °C; pale yellow oil (37.6 mg, 62%).

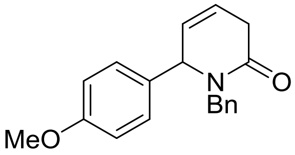

^1H NMR (400 MHz, $CDCl_3$) δ 7.35 – 7.26 (m, 3H), 7.22 – 7.17 (m, 2H), 7.12 – 7.07 (m, 2H), 6.93 – 6.87 (m, 2H), 5.78 – 5.63 (m, 2H), 5.59 (d, *J* = 15.0 Hz, 1H), 4.75 (ddd, *J* = 8.4, 2.8, 1.0 Hz, 1H), 3.81 (s, 3H), 3.43 (d, *J* = 15.0 Hz, 1H), 3.31 – 3.13 (m, 2H). ^{13}C NMR (101 MHz, $CDCl_3$) δ 167.6, 159.6, 136.9, 132.0, 128.7, 128.4, 128.3, 127.5, 126.7, 120.3, 114.5, 61.1, 55.4, 46.2, 32.2. IR (film, cm^{-1}): 3031, 3001, 2932, 2836, 1639, 1509, 1450, 1407, 1241, 1173, 1030, 909, 829, 725, 684, 496. HRMS (EI): *m/z*: calcd for $C_{19}H_{19}NO$ [M^+]: 293.14103, found: 293.14099.

Compound 3b. The reaction was performed using 3 equiv of ArMgBr at –20 °C; pale yellow oil (48.5 mg, 82%).

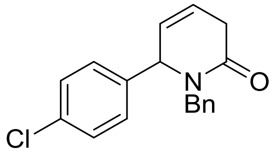

^1H NMR (400 MHz, $CDCl_3$) δ 7.36 – 7.27 (m, 5H), 7.19 – 7.15 (m, 2H), 7.14 – 7.09 (m, 2H), 5.81 – 5.57 (m, 3H), 4.79 (ddd, *J* = 3.9, 1.4 Hz, 1H), 3.39 (d, *J* = 14.9 Hz, 1H), 3.32 – 3.13 (m, 2H). ^{13}C NMR (101 MHz, $CDCl_3$) δ 167.5, 138.7, 136.6, 134.1, 129.4, 128.8, 128.5, 128.3, 127.6, 126.0, 121.1, 61.1, 46.4, 32.2. IR (film, cm^{-1}): 3087, 3062, 3029, 2927, 2852, 1642, 1450, 1404, 1246, 1089, 1014, 821, 729, 698, 495. HRMS (EI): *m/z*: calcd for $C_{18}H_{16}NOCl$ [M^+]: 297.09149, found: 297.09163.

Compound 4a. The reaction was performed using 3 equiv of ArMgBr at –45 °C to RT; pale yellow oil (50.6 mg, 91%).

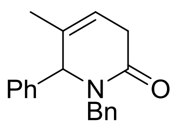

^{1}H NMR (400 MHz, $CDCl_3$) δ 7.32 – 7.17 (m, 6H), 7.15 – 7.08 (m, 4H), 5.47 (d, *J* = 15.0 Hz, 1H), 5.41 (ddp, *J* = 4.0, 2.6, 1.3 Hz, 1H), 4.40 (t, *J* = 3.2 Hz, 1H), 3.29 (d, *J* = 15.0 Hz, 1H), 3.26 – 3.04 (m, 2H), 1.43 – 1.36 (m, 3H). ^{13}C NMR (101 MHz, $CDCl_3$) δ 167.7, 140.0, 136.9, 132.5, 129.0, 128.7, 128.4, 128.3, 127.7, 127.5, 117.0, 65.6, 46.4, 32.5, 20.1. IR (film, cm^{-1}): 3085, 3062, 3028, 2917, 2851, 1643, 1449, 1247, 1065, 846, 806, 698, 498. HRMS (EI): *m/z*: calcd for $C_{19}H_{19}NO$ [M^+]: 277.14611, found: 277.14637.

Compound 4b. The reaction was performed using 3 equiv of ArMgBr at –20 °C; pale yellow oil (70.0 mg, 96%).

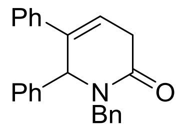

^{1}H NMR (400 MHz, $CDCl_3$) δ 7.31 – 7.12 (m, 8H), 7.11 – 7.06 (m, 3H), 7.02 (dd, *J* = 7.6, 1.8 Hz, 2H), 6.94 (dd, *J* = 6.6, 2.8 Hz, 2H), 5.90 (dd, *J* = 4.7, 2.8 Hz, 1H), 5.57 (d, *J* = 15.2 Hz, 1H), 5.14 (t, *J* = 2.6 Hz, 1H), 3.48 (d, *J* = 15.2 Hz, 1H), 3.43 – 3.26 (m, 2H). ^{13}C NMR (101 MHz, $CDCl_3$) δ 167.9, 139.3, 138.3, 138.2, 136.8, 128.92, 128.88, 128.5, 128.2, 128.0, 127.8, 127.6, 127.5, 126.7, 119.8, 63.5, 46.7, 33.1. IR (film, cm^{-1}): 3085, 3061, 3028, 2923, 1641, 1451, 1253, 1072, 908, 727, 695. HRMS (ESI): *m/z*: calcd for $C_{24}H_{21}NONa$ [$M+Na^+$]: 362.15153, found: 362.15143.

Compound 5. The reaction was performed using 3 equiv of ArMgBr; pale yellow oil (52.1 mg, 89%).

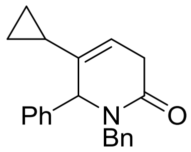

^{1}H NMR (400 MHz, $CDCl_3$) δ 7.31 – 7.18 (m, 6H), 7.17 – 7.09 (m, 4H), 5.46 (d, *J* = 15.1 Hz, 1H), 5.32 (s, 1H), 4.60 (t, *J* = 3.0 Hz, 1H), 3.35 (d, *J* = 15.1 Hz, 1H), 3.27 – 3.02 (m, 2H), 0.91 – 0.77 (m, 1H), 0.42 – 0.34 (m, 1H), 0.34 – 0.26 (m, 1H), 0.16 (dtd, *J* = 9.3, 5.5, 3.9 Hz, 1H), 0.04 (dtd, *J* = 9.5, 5.5, 4.1 Hz, 1H). ^{13}C NMR (101 MHz, $CDCl_3$) δ 167.8, 140.4, 138.1, 136.9, 128.9, 128.7, 128.3, 128.1, 127.8, 127.4, 114.2, 65.5, 46.4, 32.3, 13.9, 6.1, 5.1. IR (film, cm^{-1}): 3084, 3063, 3028, 3005, 2920, 2874, 1643, 1450, 1409, 1248, 1077, 1021, 848, 729, 699, 493. HRMS (EI): *m/z*: calcd for $C_{21}H_{21}NO$ [M^+]: 303.16176, found: 303.16186.

Compound 6. The reaction was performed using 3 equiv of ArMgBr at –20 °C; pale yellow oil (60.6 mg, 91%).

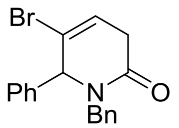

^{1}H NMR (400 MHz, $CDCl_3$) δ 7.44 – 7.29 (m, 6H), 7.26 – 7.23 (m, 2H), 7.22 – 7.17 (m, 2H), 6.13 (dd, *J* = 4.2, 3.0 Hz, 1H), 5.53 (d, *J* = 15.0 Hz, 1H), 4.76 (t, *J* = 3.4 Hz, 1H), 3.42 – 3.21 (m, 3H). ^{13}C NMR (101 MHz, $CDCl_3$) δ 165.8, 138.0, 136.2, 129.14, 129.10, 128.9, 128.4, 128.1, 127.9, 124.2, 118.7, 66.7, 46.8, 34.3. IR (film, cm^{-1}): 3085, 3062, 3029, 2920, 2850, 1645, 1449, 1405, 1243, 1072, 840, 729, 696, 496. HRMS (EI): *m/z*: calcd for $C_{18}H_{16}NOBr$ [M^+]: 341.04099, found: 341.04096.

Compound 7. The reaction was performed using 3 equiv of allylmagnesium bromide at –20 °C to RT; pale yellow oil (27.7 mg, 62%).

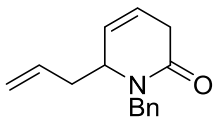

^{1}H NMR (400 MHz, $CDCl_3$) δ 7.28 – 7.13 (m, 5H), 5.75 (dtd, *J* = 10.0, 3.4, 0.9 Hz, 1H), 5.68 – 5.55 (m, 2H), 5.45 (d, *J* = 15.2 Hz, 1H), 5.06 – 4.97 (m, 2H), 3.91 (d, *J* = 15.2 Hz, 1H), 3.86 – 3.80 (m, 1H), 2.98 – 2.91 (m, 2H), 2.42 – 2.33 (m, 1H), 2.25 (dddd, *J* = 12.8, 6.5, 3.1, 1.5 Hz, 1H). ^{13}C NMR (101 MHz, $CDCl_3$) δ 168.6, 137.0, 132.1, 128.7, 128.0, 127.5, 125.8, 123.1, 119.4, 56.5, 46.5, 37.5, 32.7. IR (film, cm^{-1}): 3074, 3030,

2978, 2925, 1637, 1451, 1408, 1322, 1253, 1149, 1071, 996, 917, 703, 497. HRMS (ESI): *m/z*: calcd for $C_{15}H_{17}NONa$ [*M+Na*$^{+}$]: 250.12023, found: 250.12026.

Compound 8. The reaction was performed using 3 equiv of benyzlmagnesium bromide; white solid (50.1 mg, 91%). m.p. = 99.2-100.9 °C. ^{1}H NMR (400 MHz, $CDCl_3$) δ 7.28 – 7.22 (m, 2H), 7.21 – 7.11 (m, 6H), 7.02 – 6.95 (m, 2H), 5.63 (ddd, *J* = 10.0, 4.8, 2.0 Hz, 1H), 5.60 – 5.52 (m, 2H), 4.02 – 3.95 (m, 1H), 3.92 (d, *J* = 15.1 Hz, 1H), 2.87 (dd, *J* = 13.4, 6.8 Hz, 1H), 2.80 – 2.65 (m, 2H), 2.17 – 2.06 (m, 1H). ^{13}C NMR (101 MHz, $CDCl_3$) δ 168.9, 137.1, 135.9, 130.1, 128.8, 128.3, 127.9, 127.5, 126.8, 125.5, 124.0, 57.7, 46.8, 39.3, 32.4. IR (film, cm^{-1}): 3051, 3028, 2955, 2925, 2895, 1626, 1454, 1405, 1261, 1163, 1081, 1029, 967, 724, 693, 494. HRMS (ESI): *m/z*: calcd for $C_{19}H_{19}NONa$ [*M+Na*$^{+}$]: 300.13588, found: 300.13582.

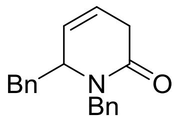

Compound 9. Yellow oil (54.8 mg, 96%). ^{1}H NMR (400 MHz, $CDCl_3$) δ 7.41 – 7.28 (m, 3H), 7.27 – 7.15 (m, 3H), 6.86 – 6.71 (m, 3H), 5.79 – 5.65 (m, 2H), 5.59 (d, *J* = 14.9 Hz, 1H), 4.83 (dd, *J* = 3.6 Hz, 1H), 3.78 (s, 3H), 3.39 (d, *J* = 14.9 Hz, 1H), 3.32 – 3.12 (m, 2H). ^{13}C NMR (101 MHz, $CDCl_3$) δ 167.6, 159.9, 140.1, 138.4, 129.7, 129.2, 128.3, 127.1, 126.5, 120.6, 120.6, 113.9, 112.9, 61.8, 55.3, 46.3, 32.2. IR (film, cm^{-1}): 3047, 3029, 3000, 2935, 2835, 1643, 1449, 1260, 1145, 1044, 838, 695, 485. HRMS (EI): *m/z*: calcd for $C_{19}H_{19}NO_2$ [*M*$^{+}$]: 293.14103, found: 293.14129.

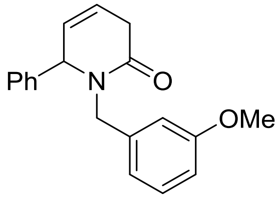

Compound 12. The reaction was performed using 3 equiv of ArMgBr; pale yellow solid (48.8 mg, 88%). m.p. = 130.6-131.1°C. ^{1}H NMR (400 MHz, $CDCl_3$) δ 7.41 – 7.29 (m, 3H), 7.21 – 7.12 (m, 4H), 6.98 (t, *J* = 8.7 Hz, 2H), 5.78 – 5.64 (m, 2H), 5.50 (d, *J* = 14.9 Hz, 1H), 4.79 (dq, *J* = 3.9, 1.2 Hz, 1H), 3.44 (d, *J* = 14.9 Hz, 1H), 3.26 (ddt, *J* = 22.3, 4.5, 2.1 Hz, 1H), 3.17 (dtd, *J* = 22.3, 3.7, 1.2 Hz, 1H). ^{13}C NMR (101 MHz, $CDCl_3$) δ 167.7, 162.3 (d, *J* = 245.5 Hz), 140.0, 132.7 (d, *J* = 3.3 Hz), 130.1 (d, *J* = 8.0 Hz), 129.2, 128.4, 127.1, 126.4, 120.6, 115.5 (d, *J* = 21.4 Hz), 61.9, 45.8, 32.2. ^{19}F NMR (282 MHz, $CDCl_3$) δ –115.1. IR (film, cm^{-1}): 3070, 3053, 3029, 3004, 2931, 2891, 1638, 1508, 1450, 1402, 1264, 1215, 1156, 1027, 920, 842, 806, 768, 699, 461. HRMS (EI): *m/z*: calcd for $C_{18}H_{16}NOF$ [*M*$^{+}$]: 281.12104, found: 281.12128.

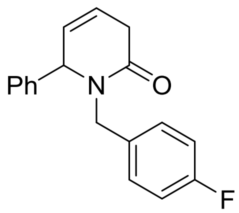

Compound 17. The reaction was performed in the presence of PPh_3 (10 mol%) to give the product as a white solid (20.4 mg, 62%). m.p. = 192.4-193.2°C. ^{1}H NMR (400 MHz, $CDCl_3$) δ 7.37 – 7.30 (m, 5H), 7.25 – 7.21 (m, 2H), 7.07 (d, *J* = 8.6 Hz, 2H), 6.03 – 5.93 (m, 2H), 5.73 (dt, *J* = 9.5, 3.6 Hz, 1H), 3.20 (dt, *J* = 3.8, 2.2 Hz, 2H), 2.34 (s, 3H). ^{13}C NMR (101 MHz, $CDCl_3$) δ 167.8, 144.6, 139.8, 135.6, 129.3, 128.9, 128.7, 128.3, 127.4, 127.1, 119.5, 62.0, 34.2, 21.6. IR (film, cm^{-1}): 3086, 3063, 3027, 2962, 2921, 1687, 1595, 1451, 1353, 1243, 1162, 1085, 811, 701, 684, 537. HRMS (ESI): *m/z*: calcd for $C_{18}H_{17}NO_3Na$ [*M+Na*$^{+}$]: 350.08214, found: 350.08245. Single crystals

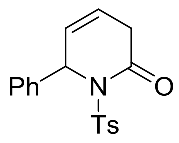

suitable for X-ray diffraction were grown by slow evaporation of a solution of this product in CH_2Cl_2/pentane.

Representative Procedure for the Iron Catalyzed Three Component Reaction. (*Z*)-1-Benzyl-3-((4-chlorophenyl)(hydroxy)methylene)-6-phenyl-3,6-dihydropyridin-2(1*H*)-one (11a). A solution of PhMgBr

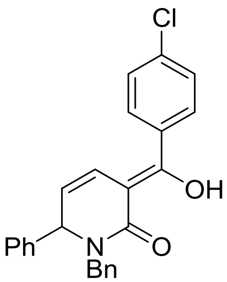

(1.18 M in THF, 0.51 mL, 0.60 mmol) was slowly added to a rapidly stirred solution of compound **1** (37.0 mg, 0.2 mmol), methyl 4-chlorobenzoate (68.2 mg, 0.4 mmol) and $Fe(acac)_3$ (3.5 mg, 0.01 mmol, 5 mol%) in THF (2 mL) at –45 °C. The mixture was stirred for 30 min at this temperature before the reaction was quenched with sat. aq. NH_4Cl. The aqueous phase was extracted with EtOAc (3 x 10 mL) and the combined organic layers were washed with brine, dried over $MgSO_4$, filtered and evaporated. The residue was purified by flash chromatography (hexane/EtOAc = 50/1, 20/1, 10/1, 6/1) to afford the title compound as a pale yellow solid (39.7 mg, 87%). m.p. = 91.7-92.9 °C. ^{1}H NMR (400 MHz, $CDCl_3$) δ 15.18 (s, 1H), 7.56 – 7.50 (m, 2H), 7.43 – 7.23 (m, 12H), 6.29 (dd, *J* = 10.1, 1.3 Hz, 1H), 5.57 (d, *J* = 15.0 Hz, 1H), 5.30 (dd, *J* = 10.1, 4.5 Hz, 1H), 5.01 (dd, *J* = 4.5, 1.1 Hz, 1H), 3.54 (d, *J* = 15.0 Hz, 1H). ^{13}C NMR (101 MHz, $CDCl_3$) δ 169.6, 167.4, 140.5, 136.4, 136.1, 133.2, 130.4, 129.3, 128.9, 128.7, 128.5, 128.3, 127.8, 127.1, 121.0, 119.5, 98.8, 62.7, 46.4. IR (film, cm^{-1}): 3085, 3059, 3028, 2977, 2950, 2925, 1724, 1654, 1594, 1469, 1450, 1341, 1264, 1089, 1014, 906, 836, 728, 523. HRMS (ESI): *m/z*: calcd for $C_{25}H_{21}ClNO_2$ [$M+H^+$]: 402.12553, found: 402.12548. Single crystals suitable for X-ray diffraction were grown by slow evaporation of a solution of this product in CH_2Cl_2/pentane.

Compound 11b. Prepared analogously using methyl 3-phenylpropanoate as the electrophilic coupling

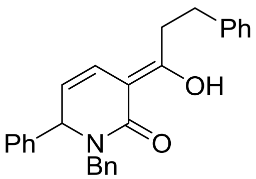

partner; pale yellow oil (76.6 mg, 93%). ^{1}H NMR (400 MHz, $CDCl_3$) δ 14.94 (s, 1H), 7.31 – 7.07 (m, 15H), 6.09 (dd, *J* = 10.1, 1.3 Hz, 1H), 5.42 (d, *J* = 15.1 Hz, 1H), 5.10 (dd, *J* = 10.1, 4.3 Hz, 1H), 4.85 (d, *J* = 4.2 Hz, 1H), 3.37 (d, *J* = 15.1 Hz, 1H), 2.91 (t, *J* = 7.9 Hz, 2H), 2.70 – 2.52 (m, 2H). ^{13}C NMR (101 MHz, $CDCl_3$) δ 171.7, 169.3, 141.0, 140.8, 136.6, 133.2, 129.2, 128.8, 128.6, 128.4, 128.2, 127.6, 127.2, 126.3, 119.8, 118.1, 98.1, 62.6, 46.1, 33.4, 33.1. IR (film, cm^{-1}): 3085, 3061, 3027, 2930, 2863, 1736, 1654, 1596, 1469, 1451, 1358, 1256, 1211, 1028, 912, 747, 723, 696, 510. HRMS (ESI): *m/z*: calcd for $C_{27}H_{26}NO_2$ [$M+H^+$]: 396.19580, found: 396.19585.

Representative Procedure for the Iron Catalyzed Formal Ring-opening/Cross Coupling of 2-Pyridone

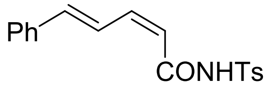

Deravitives. (2*Z*,4*E*)-5-Phenyl-N-tosylpenta-2,4-dienamide (18a). A solution of PhMgBr (1 M in THF, 0.6 mL, 0.6 mmol) was added dropwise to a rapidly stirred solution of compound **16** (49.9 mg, 0.2 mmol), $Fe(acac)_3$ (3.5 mg, 0.01 mmol, 5 mol%) and PPh_3 (5.2 mg, 0.02 mmol, 10 mol%) in Et_2O (2 mL) at –30 °C. The mixture was stirred for 30 min at this temperature before DMF (4 mL) was introduced and the temperature raised to –10 °C

over the course of 2 h. The reaction was quenched with sat. aq. NH_4Cl and the aqueous phase was extracted with EtOAc (3 x 10 mL). The combined organic layers were washed with brine, dried over $MgSO_4$, filtered and evaporated. The residue was purified by flash chromatography (pentane/ethyl acetate = 4/1, 2/1, 1/1) to afford the title compound as a pale yellow solid (38.3 mg, 60%, 2*Z*/2*E* = 15/1). m.p. = 139.1-141.2 °C. ^{1}H NMR (400 MHz, $CDCl_3$) δ 8,71 (s, 1H), 8.10 (ddd, *J* = 15.7, 11.5, 0.8 Hz, 1H), 8.00 (d, *J* = 8.4 Hz, 2H), 7.52 – 7.45 (m, 2H), 7.38 – 7.29 (m, 5H), 6.80 (d, *J* = 15.7 Hz, 1H), 6.71 (t, *J* = 11.5 Hz, 1H), 5.62 (d, *J* = 11.1 Hz, 1H), 2.43 (s, 3H). ^{13}C NMR (101 MHz, $CDCl_3$) δ 163.0, 147.5, 145.2, 143.8, 136.03, 136.00, 129.8, 129.6, 128.9, 128.5, 128.0, 124.8, 115.8, 21.9. IR (film, cm^{-1}): 3253, 3074, 3057, 3025, 2922, 2873, 1697, 1615, 1592, 1435, 1329, 1235, 1122, 1083, 984, 878, 657, 542. HRMS (ESI): *m/z*: calcd for $C_{18}H_{17}NO_3SNa$ [$M+Na^+$]: 350.08214, found: 350.08242. Single crystals suitable for X-ray diffraction were grown by slow evaporation of a solution of this product in CH_2Cl_2/pentane.

The following compounds were prepared analogously:

Compound 18b. Pale yellow oil (47.5 mg, 70%, 2*Z*/2*E* = 15/1). ^{1}H NMR (400 MHz, $CDCl_3$) δ 9.10 (s, 1H), 8.16 – 8.03 (m, 3H), 7.47 (dd, *J* = 7.8, 1.8 Hz, 2H), 7.35 – 7.27 (m, 3H), 7.03 – 6.95 (m, 2H), 6.79 (d, *J* = 15.7 Hz, 1H), 6.70 (t, *J* = 11.2 Hz, 1H), 5.66 (d, *J* = 11.2 Hz, 1H), 3.84 (s, 3H). ^{13}C NMR (101 MHz, $CDCl_3$) δ 164.0, 163.4, 147.2, 143.5, 136.0, 130.7, 130.2, 129.5, 128.8, 127.9, 124.8, 116.2, 114.4, 55.8. IR (film, cm^{-1}): 3239, 3077, 3064, 2929, 2841, 1688, 1590, 1497, 1433, 1335, 1261, 1163, 1124, 1080, 1023, 864, 832, 803, 729, 665, 576, 553. HRMS (ESI): *m/z*: calcd for $C_{18}H_{17}NO_4SNa$ [$M+Na^+$]: 366.07705, found: 366.07737.

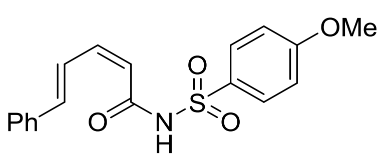

Compound 18c. Pale yellow solid (32.4 mg, 47%, 2*Z*/2*E* = 18/1). m.p. = 69.0-71.4 °C. ^{1}H NMR (400 MHz, $CDCl_3$) δ 9.01 (s, 1H), 8.20 – 8.13 (m, 2H), 8.09 (ddd, *J* = 15.7, 11.5, 1.0 Hz, 1H), 7.47 (dd, *J* = 7.6, 1.8 Hz, 2H), 7.35 – 7.28 (m, 3H), 7.22 (t, *J* = 8.6 Hz, 2H), 6.81 (d, *J* = 15.7 Hz, 1H), 6.73 (t, *J* = 11.6 Hz, 1H), 5.65 (d, *J* = 11.1 Hz, 1H). ^{13}C NMR (101 MHz, $CDCl_3$) δ 166.0 (d, *J* = 256.8 Hz), 163.2, 147.8, 144.1, 135.9, 134.8, 131.5 (d, *J* = 9.7 Hz), 129.7, 128.9, 127.9, 124.7, 116.5 (d, *J* = 22.8 Hz), 115.7. ^{19}F NMR (282 MHz, $CDCl_3$) δ –102.9. IR (film, cm^{-1}): 3245, 3106, 3074, 2859, 1692, 1612, 1586, 1431, 1338, 1122, 1079, 868, 838, 754, 665, 576, 544. HRMS (ESI): *m/z*: calcd for $C_{17}H_{13}FNO_3S$ [M^-]: 330.06057, found: 330.06064.

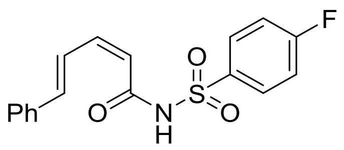

Compound 19a. Pale yellow solid (55.0 mg, 74%, 2*Z*/2*E* = 8/1). m.p. = 162.8-163.5 °C. ^{1}H NMR (400 MHz, $CDCl_3$) δ 8.57 (s, 1H), 8.09 – 7.94 (m, 3H), 7.44 (d, *J* = 8.8 Hz, 2H), 7.00 (d, *J* = 9.0 Hz, 2H), 6.85 (d, *J* = 8.8 Hz, 2H), 6.76 (d, *J* = 15.5 Hz, 1H), 6.69 (t, *J* = 11.2 Hz, 1H), 5.54 (d, *J* = 11.2 Hz, 1H), 3.86 (s, 3H), 3.81 (s, 3H). ^{13}C NMR (101 MHz, $CDCl_3$) δ 164.0, 163.3, 160.9, 147.8, 143.6, 130.8, 130.5, 129.5, 129.1,

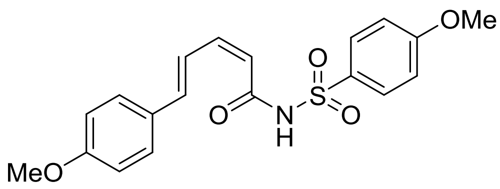

128.9, 122.9, 114.7, 114.4, 55.8, 55.5. IR (film, cm^{-1}): 3270, 3064, 2984, 2933, 2910, 2872, 1687, 1589, 1500, 1417, 1252, 1162, 1114, 1080, 1018, 993, 834, 799, 668, 556. HRMS (ESI): *m/z*: calcd for $C_{19}H_{18}NO_5S$ [M^-]: 372.09112, found: 372.09120.

Compound 19b. Pale yellow solid (45.1 mg, 61%, 2*Z*/2*E* = 13/1). m.p. = 160.3-161.5 °C. ^{1}H NMR (400 MHz, $CDCl_3$) δ 8.61 (s, 1H), 8.13 – 7.99 (m, 3H), 7.41 (d, *J* = 8.5 Hz, 2H), 7.29 (d, *J* = 8.5 Hz, 2H), 7.02 (d, *J* = 9.0 Hz, 2H), 6.79 – 6.64 (m, 2H), 5.62 (d, *J* = 11.1 Hz, 1H), 3.87 (s, 3H). ^{13}C NMR (101 MHz, $CDCl_3$) δ 164.1, 163.0, 146.9, 142.0, 135.3, 134.6, 130.8, 130.2, 129.1, 129.0, 125.3, 116.5, 114.4, 55.9. IR (film, cm^{-1}): 3272, 3064, 3039, 2978, 2946, 2841, 1691, 1591, 1498, 1409, 1264, 1161, 1079, 994, 830, 797, 666, 556. HRMS (ESI): *m/z*: calcd for $C_{18}H_{15}ClNO_4S$ [*M-H*]: 376.04158, found: 376.04179.

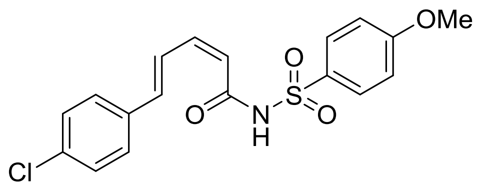

Compound 20. Pale yellow solid (52.4 mg, 69%, 2*Z*/2*E* = 23/1). m.p. = 215.4-217.2 °C. ^{1}H NMR (400 MHz, $CDCl_3$) δ 9.14 (s, 1H), 8.14 – 8.00 (m, 3H), 7.06 – 6.93 (m, 4H), 6.76 – 6.61 (m, 3H), 5.72 (d, *J* = 11.2 Hz, 1H), 3.87 (s, 3H). ^{13}C NMR (101 MHz, $CDCl_3$) δ 164.1, 163.2 (dd, *J* = 248.6, 12.9 Hz), 163.1, 145.8, 140.4 (t, *J* = 3.1 Hz), 139.4 (t, *J* = 9.5 Hz), 130.8, 130.0, 127.1, 118.1, 114.4, 110.3 (dd, *J* = 25.7, 6.9 Hz), 104.5 (t, *J* = 25.6 Hz). ^{19}F NMR (282 MHz, $CDCl_3$) δ –109.7. IR (film, cm^{-1}): 3228, 3086, 2983, 2871, 2845, 1733, 1706, 1593, 1431, 1334, 1259, 1165, 1115, 1079, 980, 851, 830, 663, 553. HRMS (ESI): *m/z*: calcd for $C_{18}H_{14}F_2NO_4S$ [*M-H*]: 378.06171, found: 378.06186.

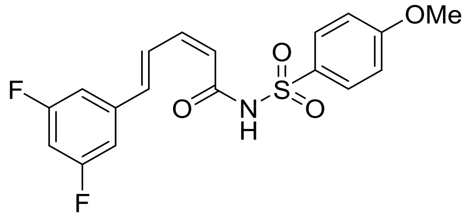

Compound 21. Pale yellow solid (34.1 mg, 55%, 2*Z*/2*E* = 18/1). m.p. = 134.1-134.9 °C. ^{1}H NMR (400 MHz, $CDCl_3$) δ 8.08 (ddd, *J* = 15.7, 11.5, 0.9 Hz, 1H), 7.88 (dd, *J* = 3.8, 1.2 Hz, 1H), 7.62 (dd, *J* = 5.0, 1.2 Hz, 1H), 7.47 – 7.41 (m, 2H), 7.31 – 7.23 (m, 3H), 7.06 (dd, *J* = 4.9, 3.9 Hz, 1H), 6.78 (d, *J* = 15.7 Hz, 1H), 6.70 (t, *J* = 11.5 Hz, 1H), 5.55 (dt, *J* = 11.0, 0.9 Hz, 1H). ^{13}C NMR (101 MHz, $CDCl_3$) δ 163.0, 147.8, 144.1, 139.3, 136.0, 135.2, 134.0, 129.7, 128.9, 128.0, 127.6, 124.8, 115.7. IR (film, cm^{-1}): 3245, 3098, 3026, 2873, 1685, 1612, 1585, 1433, 1340, 1178, 1123, 1090, 1019, 876, 809, 726, 591, 567. HRMS (ESI): *m/z*: calcd for $C_{15}H_{13}NO_3S_2Na$ [$M+Na^+$]: 342.02291, found: 342.02329.

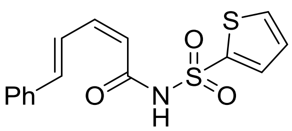

Compound 27. A solution of PhMgBr (3 M in Et_2O, 0.1 mL, 0.3 mmol) was slowly added to a rapidly stirred solution of 2-pyrone (19.2 mg, 0.2 mmol) and $Fe(acac)_3$ (3.5 mg, 0.01 mmol, 5 mol%) in Et_2O (2 mL) at –30 °C. After stirring for 20 min at this temperature, the reaction was quenched with sat. aq. NH_4Cl and the pH of the aqueous layer adjusted to ≈ 2-3 upon addition of HCl (1 M). The aqueous phase was extracted with EtOAc (5 x 20 mL), the combined organic layers were washed with brine, dried over $MgSO_4$, filtered and evaporated. The residue was purified by flash chromatography (pentane/EtOAc = 10/1, 4/1, 2/1) to afford the title

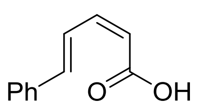

compound as a pale yellow solid material (15.5 mg, 44%). m.p. = 130.2-130.9 °C. ^{1}H NMR (400 MHz, $CDCl_3$) δ 11.20 (s, 1H), 8.11 (ddd, *J* = 15.3, 11.6, 1.1 Hz, 1H), 7.55 (dd, *J* = 8.2, 1.2 Hz, 2H), 7.42 – 7.29 (m, 3H), 6.93 – 6.82 (m, 2H), 5.77 (d, *J* = 11.0 Hz, 1H). ^{13}C NMR (101 MHz, $CDCl_3$) δ 171.4, 147.2, 142.6, 136.3, 129.4, 128.9, 127.8, 125.0, 116.5. IR (film, cm^{-1}): 3033, 2953, 2922, 2825, 2745, 2565, 1685, 1608, 1585, 1434, 1246, 1227, 958, 821, 744, 698. HRMS (ESI): *m/z*: calcd for $C_{11}H_9NO_2$ [*M-H*]: 173.06081, found: 173.06071. Single crystals suitable for X-ray diffraction were grown by slow evaporation of a solution of this product in CH_2Cl_2/pentane.

Control Experiments

Compound 14. Triethylamine (0.6 mL, 4.3 mmol, 1.5 equiv), HOBt (0.43 g, 3.15 mmol) and EDC·HCl (0.83 g, 4.3 mmol) were added to a solution of benzylamine (0.34 mL, 3.15 mmol) and acid **13** (0.4 g, 2.9 mmol)[3] in DMF (1.5 mL) and CH_2Cl_2 (20 mL). The mixture was stirred for 4 h before the solvent was evaporated. The residue was suspended in water (30 mL) and extracted from the aqueous phase with EtOAc (3 x 20 mL). The combined organic layers were washed with aq. citric acid solution (0.5 M), sat. aq. $NaHCO_3$ and brine, dried over $MgSO_4$, and evaporated. The residue was purified by flash chromatography (hexane/EtOAc = 6/1, 4/1, 2/1, 1/1) to give the title compound as a pale orange oil (0.61 g, 92%). ^{1}H NMR (400 MHz, $CDCl_3$) δ 7.36 – 7.30 (m, 2H), 7.29 – 7.24 (m, 3H), 6.27 (s, 1H), 5.96 (s, 1H), 5.73 (td, *J* = 1.4, 1.3 Hz, 1H), 4.45 (d, *J* = 5.5 Hz, 2H), 1.89 (dd, *J* = 1.4, 0.7 Hz, 3H), 1.68 (d, *J* = 1.4 Hz, 3H), 1.60 (d, *J* = 1.3 Hz, 3H). ^{13}C NMR (101 MHz, $CDCl_3$) δ 166.6, 146.1, 138.7, 138.6, 128.8, 128.0, 127.6, 123.7, 122.3, 43.8, 26.0, 25.5, 19.7. IR (film, cm^{-1}): 3306, 3064, 3031, 2975, 2929, 2874, 1724, 1646, 1525, 1454, 1376, 1265, 1075, 1029, 733, 697. HRMS (EI): *m/z*: calcd for $C_{15}H_{19}NO$ [M^+]: 229.14611, found: 229.14612.

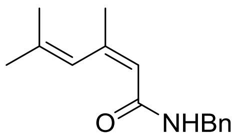

Compound 15. LiHMDS (40.7 mg, 0.24 mmol, 2.4 equiv) was added to a stirred solution of amide **14** (47.2 mg, 0.2 mmol, 1 equiv) in DMF (2 mL) and the resulting mixture was stirred at 100 °C for 3 h. After reaching ambient temperature, the reaction was quenched with sat. aq. NH_4Cl and the aqueous phase was extracted with EtOAc (3 x 10 mL). The combined organic layers were washed with brine, dried over $MgSO_4$, filtered and concentrated, and the residue was purified by flash chromatography (pentane/EtOAc, 5:1 to 1/1) to afford the title compound as a white solid material (31.8 mg, 67%). m.p. = 77.7-78.5 °C. ^{1}H NMR (400 MHz, $CDCl_3$) δ 7.31 – 7.15 (m, 5H), 5.86 (t, *J* = 1.5 Hz, 1H), 4.66 (s, 2H), 2.24 (t, *J* = 1.1 Hz, 2H), 1.89 (s, 3H), 1.20 (s, 6H). ^{13}C NMR (101 MHz, $CDCl_3$) δ 165.9, 148.4, 140.3, 128.4, 127.1, 126.6, 120.1, 56.6, 44.6, 44.1, 26.8, 23.0. IR (film, cm^{-1}): 3061, 3031, 2980, 2930, 1668, 1605, 1433, 1409, 1350, 1192, 1029, 885, 847, 718, 693. HRMS (ESI): *m/z*: calcd for $C_{15}H_{19}NONa$ [$M+Na^+$]: 252.13588, found: 252.13574.

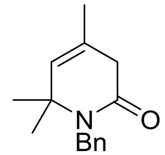

Iron Complexes

Preparation of Adduct [22·(4,6-dimethyl-2*H*-pyran-2.one]. $AgBF_4$ (227 mg, 1.2 mmol) was added to a solution of $FeCl_2(THF)_{1.5}$ (143 mg, 0.61 mmol) in THF (3 mL), causing the formation of a white precipitate. The suspension was stirred for 2 h before the mixture was filtered through a pad of Celite under Ar, which was thoroughly washed with THF (8 mL). 4,6-Dimethyl-2-pyrone **12** (455 mg, 3.7 mmol) was added to the combined filtrates, resulting in the appearance of a yellow color. The mixture was stirred for 5 h before all volatile materials were evaporated. The remaining yellow solid was rinsed with pentane and Et_2O and then dried in vacuo to give the title complex as a brown crystalline material (528 mg, 79%) which is an adduct of complex 22 with unbound 4,6-dimethyl-2*H*-pyran-2.one, see Figure S-7. Single crystals suitable for X-ray diffraction were obtained by slow evaporation of a solution of this complex in CH_2Cl_2.

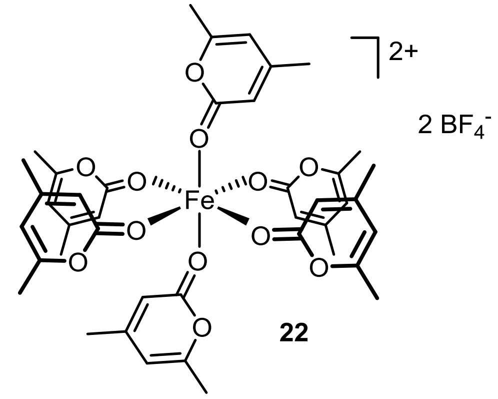

22

(Tricarbonyl)Iron Pyrone Complex 23. A Schlenk flask was charged with $Fe_2(CO)_9$ (293 mg, 0.81 mmol) and 2-pyrone **12** (200 mg, 1.61 mmol) under Ar. Degassed anhydrous *n*-Bu_2O/THF (*v/v* 5/1, 24 mL) was added and the solution was stirred at 65 °C for 0.5 h while argon was slowly bubbled through the mixture. Two further portions of $Fe_2(CO)_9$ (291 mg, 0.81 mmol each) were added at 0.5 h intervals. After stirring for another 2 h, the mixture was allowed to reach ambient temperature before the solvent was evaporated. Purification of the residue by flash chromatography (hexane/EtOAc = 10/1 to 4/1) yielded the title compound as a yellow solid material (92.6 mg, 19%). m. p. (decomp.) = 100.4-101.2 °C. ^{1}H NMR (400 MHz, $CDCl_3$) δ 5.43 (s, 1H), 2.99 (d, *J* = 1.6 Hz, 1H), 2.41 (s, 3H), 1.86 (s, 3H).^{13}C NMR (101 MHz, $CDCl_3$) δ 208.0, 170.7, 105.8, 100.9, 78.0, 53.1, 22.6, 20.4. IR (film, cm^{-1}): 3065, 2992, 2961, 2927, 2895, 2055, 1973, 1715, 1439, 1356, 1264, 1167, 1052, 1001, 860, 754, 606, 568. HRMS (ESI): *m/z*: calcd for $C_{10}H_8FeO_5Na$ [*M+Na*$^+$]: 286.96133, found: 286.96122. Single crystals suitable for X-ray diffraction were grown by slowly lowering the temperature of a saturated solution of this product in EtOAc/pentane from +20 °C to –30 °C.

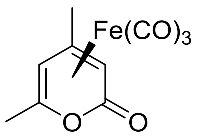

(Tricarbonyl)iron Pyridone Complex 24. Prepared analogously as a yellow solid material (177 mg, 21%). m. p. (decomp.) = 183.4-184.7 °C. ^{1}H NMR (400 MHz, CD_2Cl_2) δ 7.75 (d, *J* = 8.0 Hz, 2H), 7.34 (d, *J* = 8.0 Hz, 2H), 6.20 – 6.10 (m, 1H), 5.76 – 5.68 (m, 1H), 5.22 (dt, *J* = 5.4, 2.6 Hz, 1H), 2.89 (dt, *J* = 6.9, 2.3 Hz, 1H), 2.43 (s, 3H). ^{13}C NMR (101 MHz, CD_2Cl_2) δ 207.7, 170.3, 145.9, 135.5, 130.1, 128.6, 90.3, 77.1, 66.3, 53.8, 22.0. IR (film, cm^{-1}): 3079, 2922, 2064,

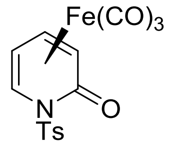

1992, 1698, 1594, 1347, 1158, 1084, 608, 546. HRMS (Exactive): *m*/*z*: calcd for $C_{15}H_{11}FeNO_6SNa$ [$M+Na^+$]: 411.95487, found: 411.95461. Single crystals suitable for X-ray diffraction were grown by slowly lowering the temperature of a saturated solution of this product in EtOAc/pentane from 20 °C to –30 °C.

References

(1) Falb, E.; Ulanenko, K.; Tor, A.; Gottesfeld, R.; Weitman, M.; Afri, M.; Gottlieb, H.; Hassner, A. *Green Chem*. **2017**, *19*, 5046.

(2) Sugahara, M.; Ukita, T. *Chem. Pharm. Bull.* **1997**, *45*, 719.

(3) Sun, C.-L.; Fürstner, A. *Angew. Chem. Int. Ed*. **2013**, *52*, 13071.

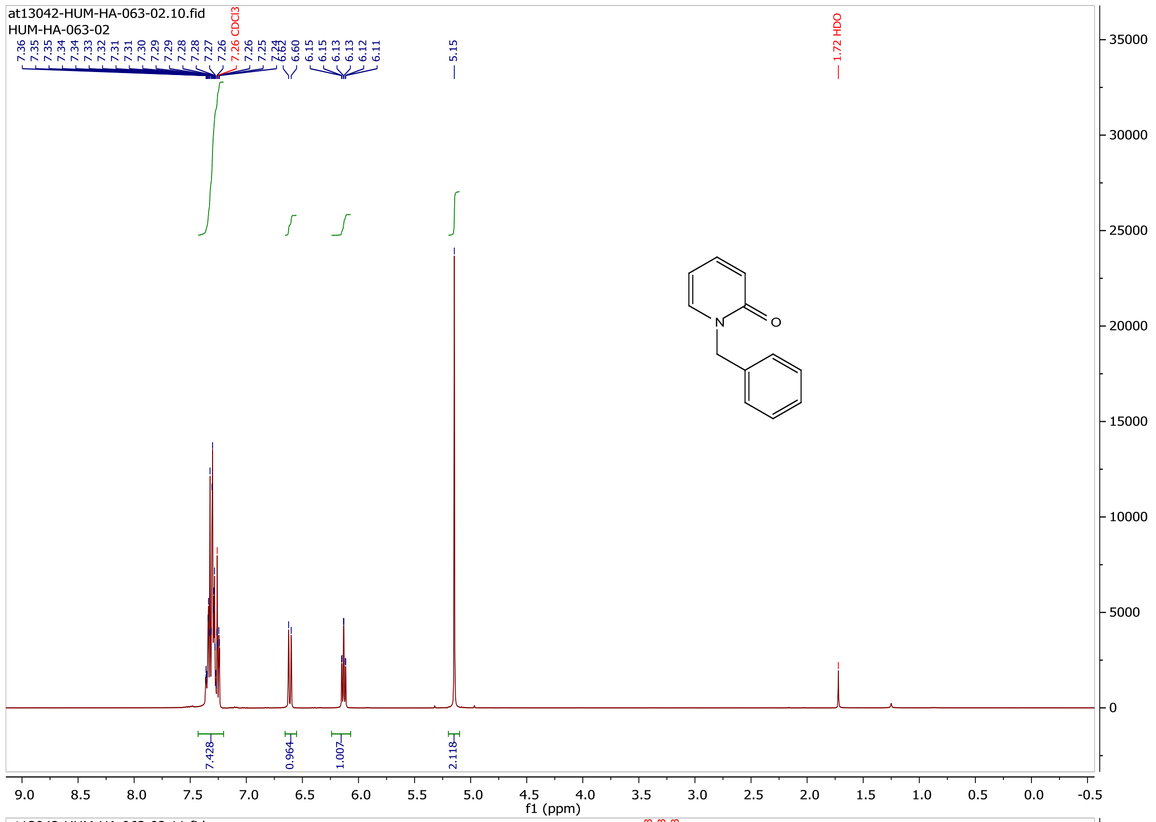
at13042-HUM-HA-063-02.10.fid
HUM-HA-063-02
7.36 7.35 7.35 7.34 7.34 7.33 7.32 7.31 7.31 7.30 7.29 7.29 7.28 7.28 7.27 7.26 7.26 CDCl3 7.26 7.25 7.24 6.62 6.60 6.15 6.15 6.13 6.13 6.12 6.11
5.15
1.72 HDO
N
O
7.428
0.964
1.007
2.118
f1 (ppm)

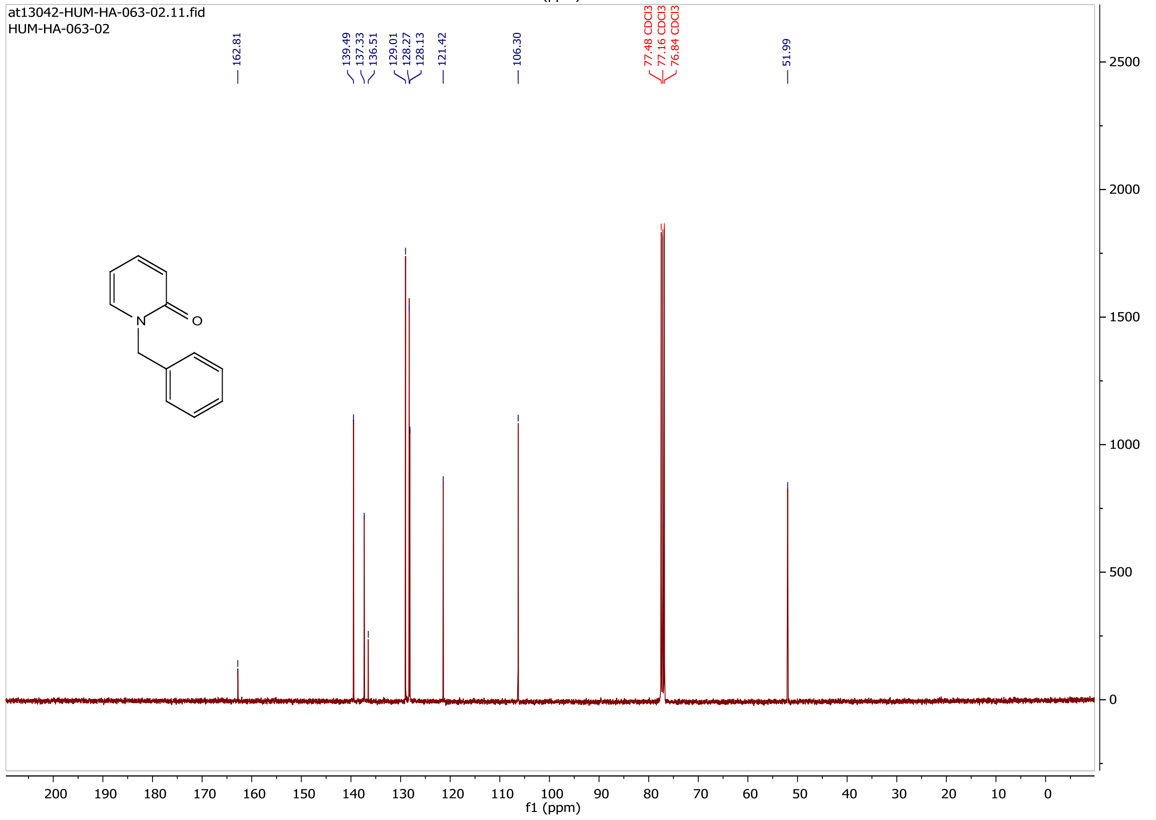
at13042-HUM-HA-063-02.11.fid
HUM-HA-063-02
162.81
139.49 137.33 136.51
129.01 128.27 128.13
121.42
106.30
77.48 CDCl3 77.16 CDCl3 76.84 CDCl3
51.99
N
O
f1 (ppm)

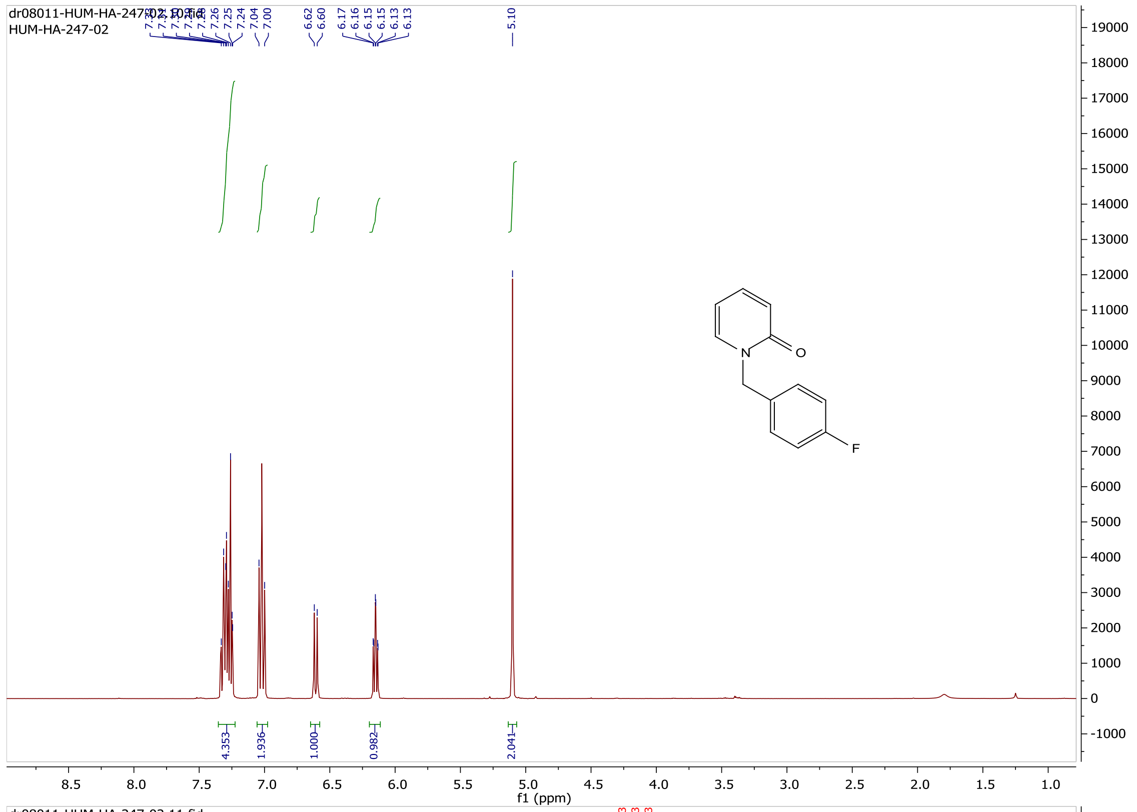
dr08011-HUM-HA-247-02.10.fid
HUM-HA-247-02
7.26
7.25
7.24
7.04
7.00
6.62
6.60
6.17
6.16
6.15
6.15
6.13
6.13
5.10
4.353
1.936
1.000
0.982
2.041
N
O
F
f1 (ppm)

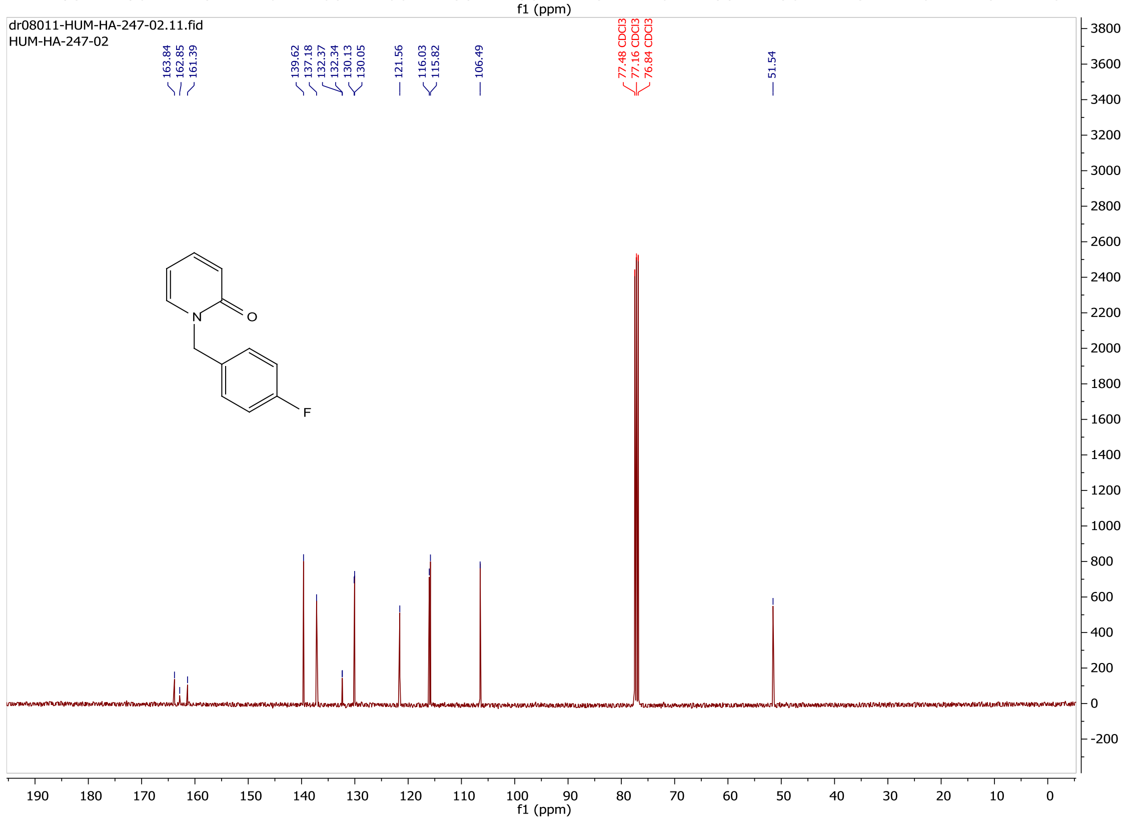
dr08011-HUM-HA-247-02.11.fid
HUM-HA-247-02
163.84
162.85
161.39
139.62
137.18
132.37
132.34
130.13
130.05
121.56
116.03
115.82
106.49
77.48 CDCl3
77.16 CDCl3
76.84 CDCl3
51.54
N
O
F
f1 (ppm)

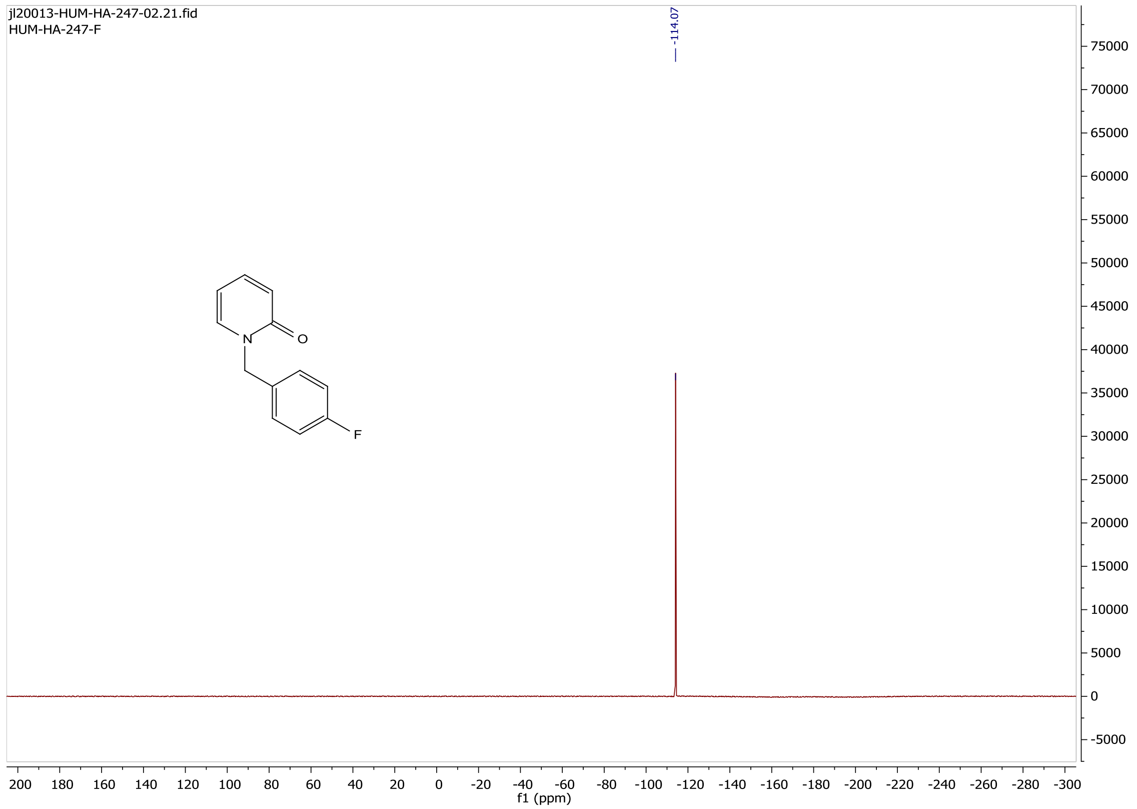
jl20013-HUM-HA-247-02.21.fid
HUM-HA-247-F
-114.07
N
O
F
75000
70000
65000
60000
55000
50000
45000
40000
35000
30000
25000
20000
15000
10000
5000
0
-5000
200 180 160 140 120 100 80 60 40 20 0 -20 -40 -60 -80 -100 -120 -140 -160 -180 -200 -220 -240 -260 -280 -300
f1 (ppm)

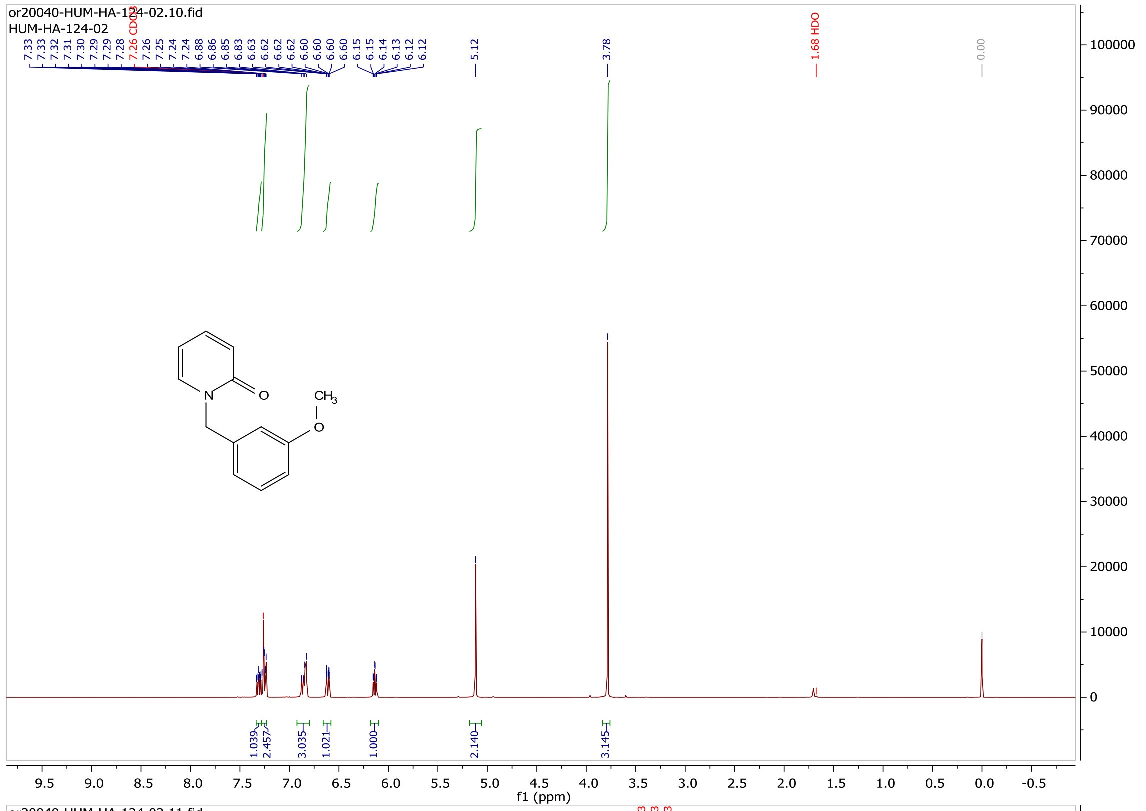

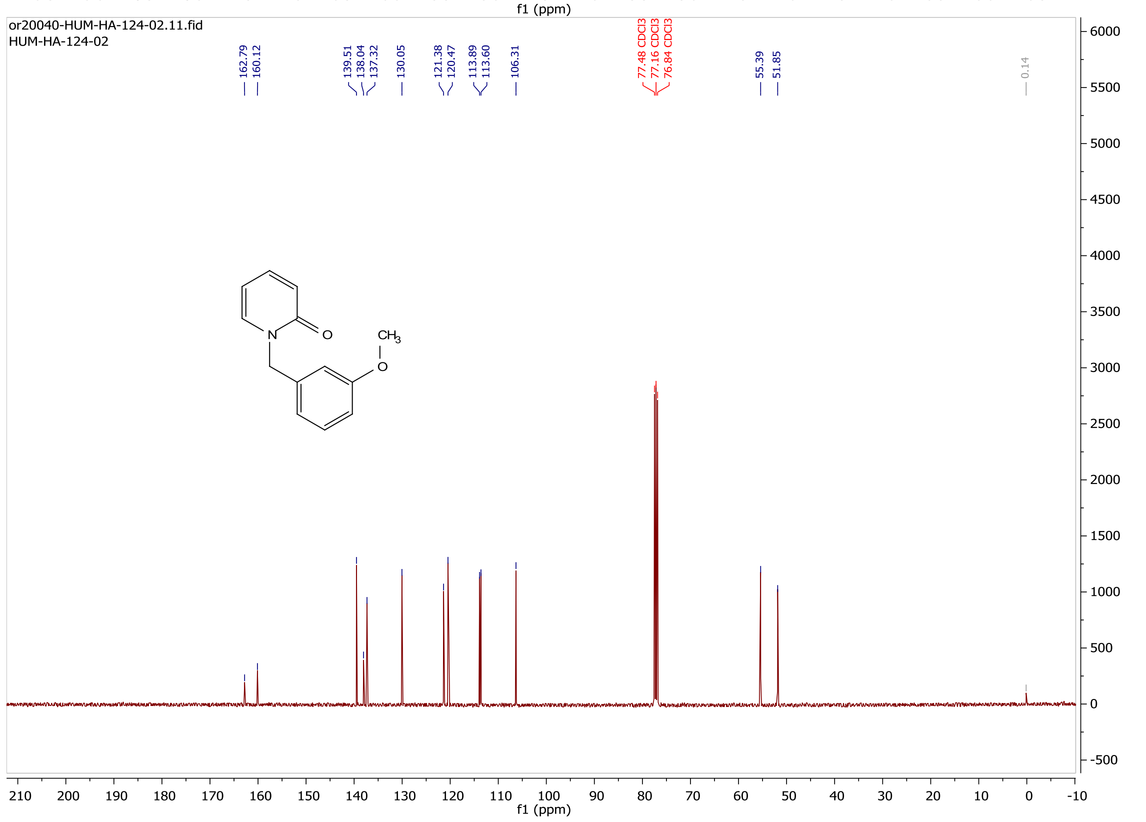

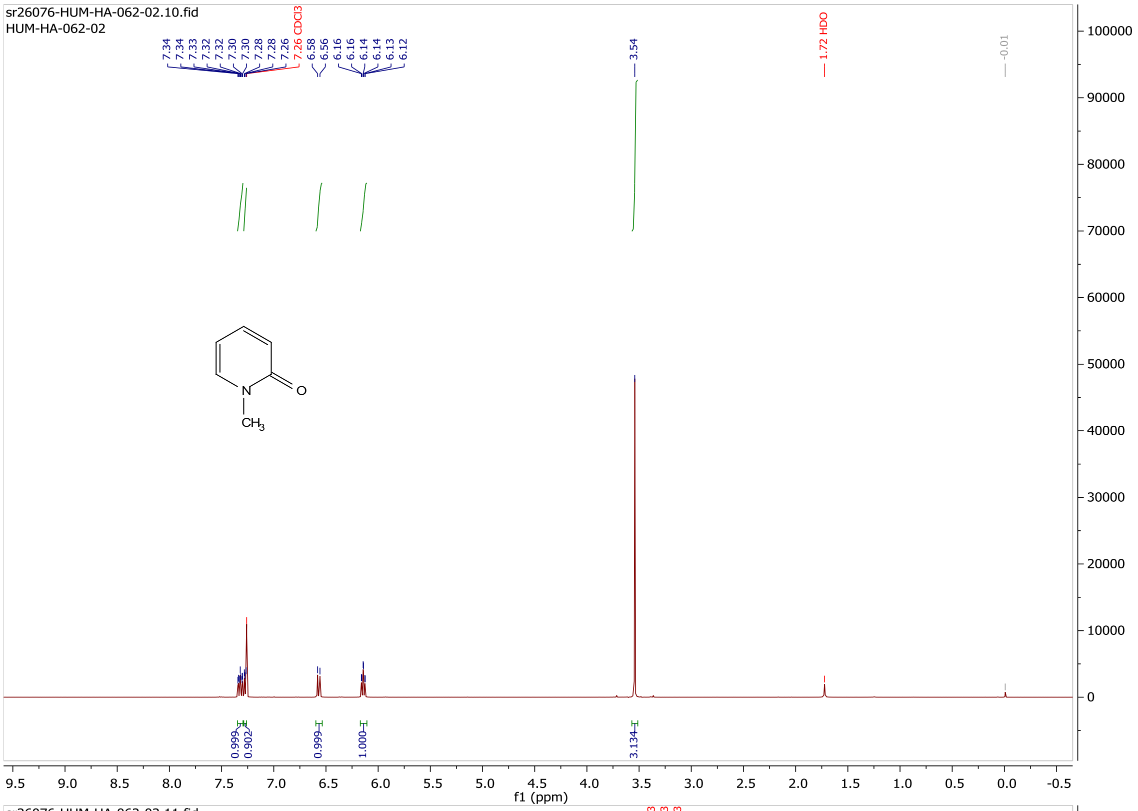
sr26076-HUM-HA-062-02.10.fid
HUM-HA-062-02
7.34 7.34 7.33 7.32 7.32 7.30 7.30 7.28 7.28 7.26 7.26 CDCl3 6.58 6.56 6.16 6.16 6.14 6.14 6.13 6.12
3.54
1.72 HDO
-0.01
N
O
CH3
0.999 0.902 0.999 1.000 3.134
f1 (ppm)

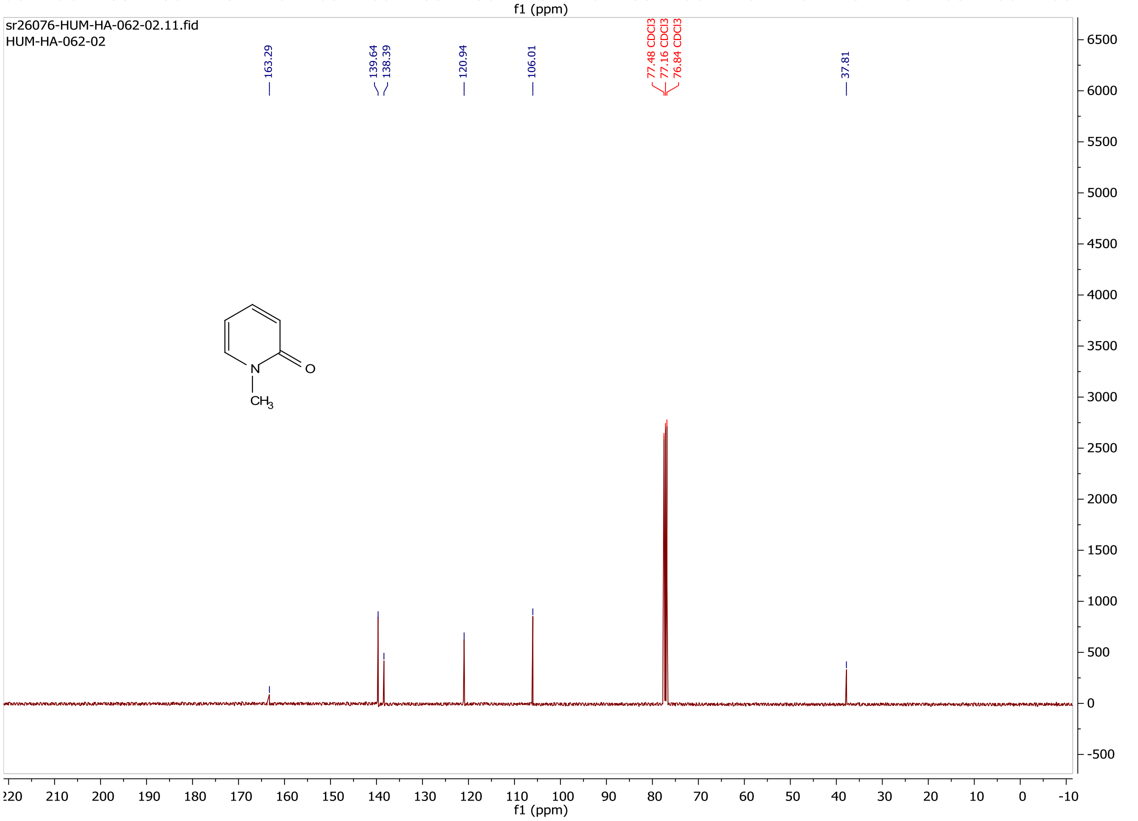
sr26076-HUM-HA-062-02.11.fid
HUM-HA-062-02
163.29
139.64 138.39
120.94
106.01
77.48 CDCl3 77.16 CDCl3 76.84 CDCl3
37.81
N
O
CH3
f1 (ppm)

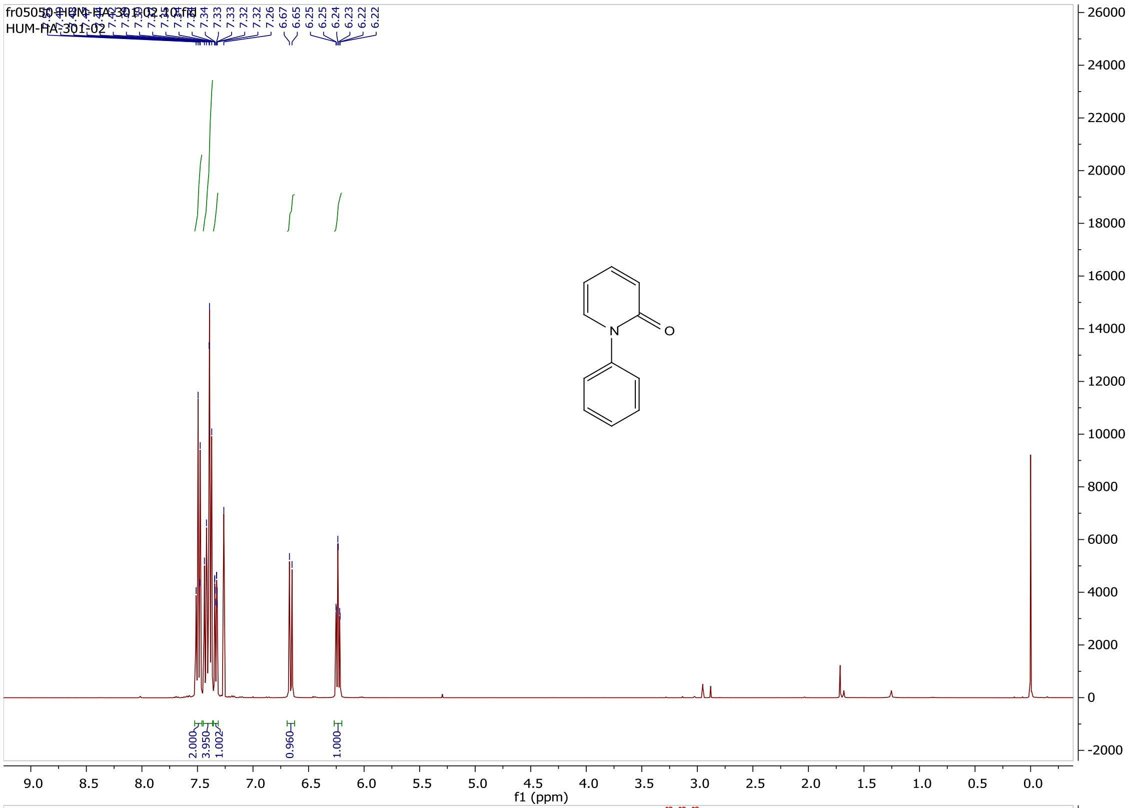
HUM-HA-301-02
7.34
7.33
7.33
7.32
7.32
7.26
6.67
6.65
6.25
6.25
6.24
6.23
6.22
6.22
2.000
3.950
1.002
0.960
1.000
N
O
f1 (ppm)

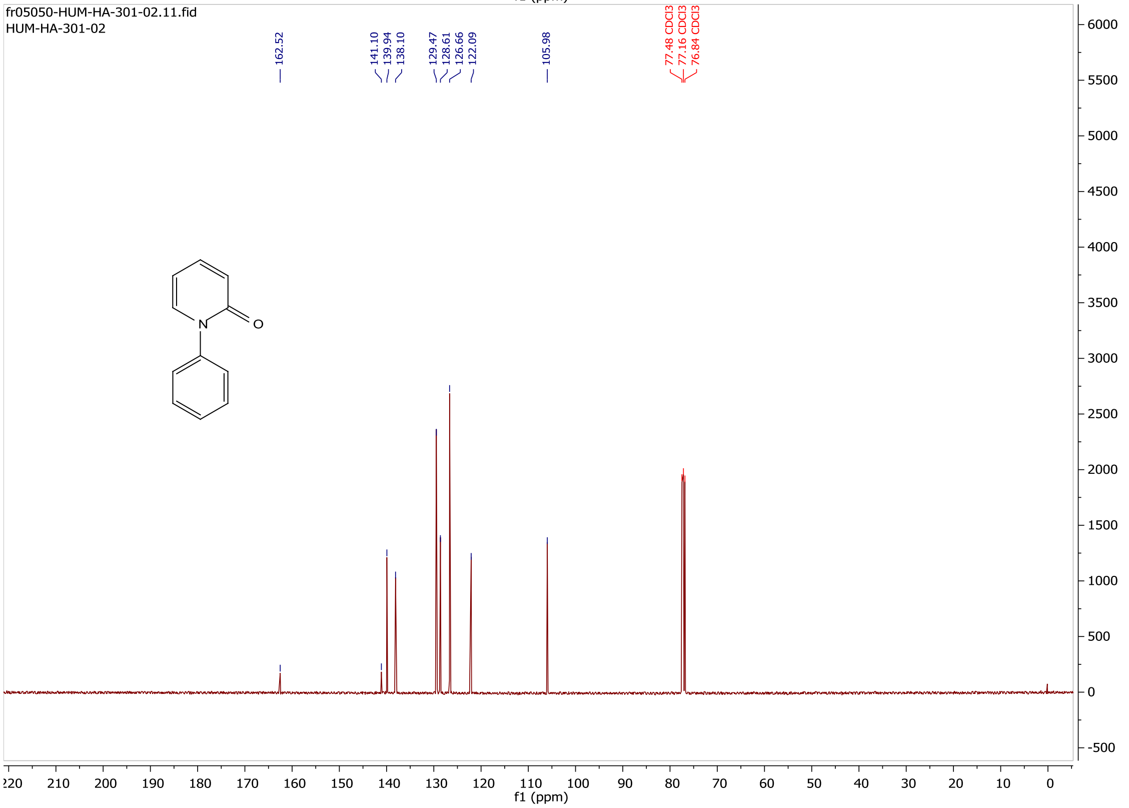
fr05050-HUM-HA-301-02.11.fid
HUM-HA-301-02
162.52
141.10
139.94
138.10
129.47
128.61
126.66
122.09
105.98
77.48 CDCl3
77.16 CDCl3
76.84 CDCl3
N
O
f1 (ppm)

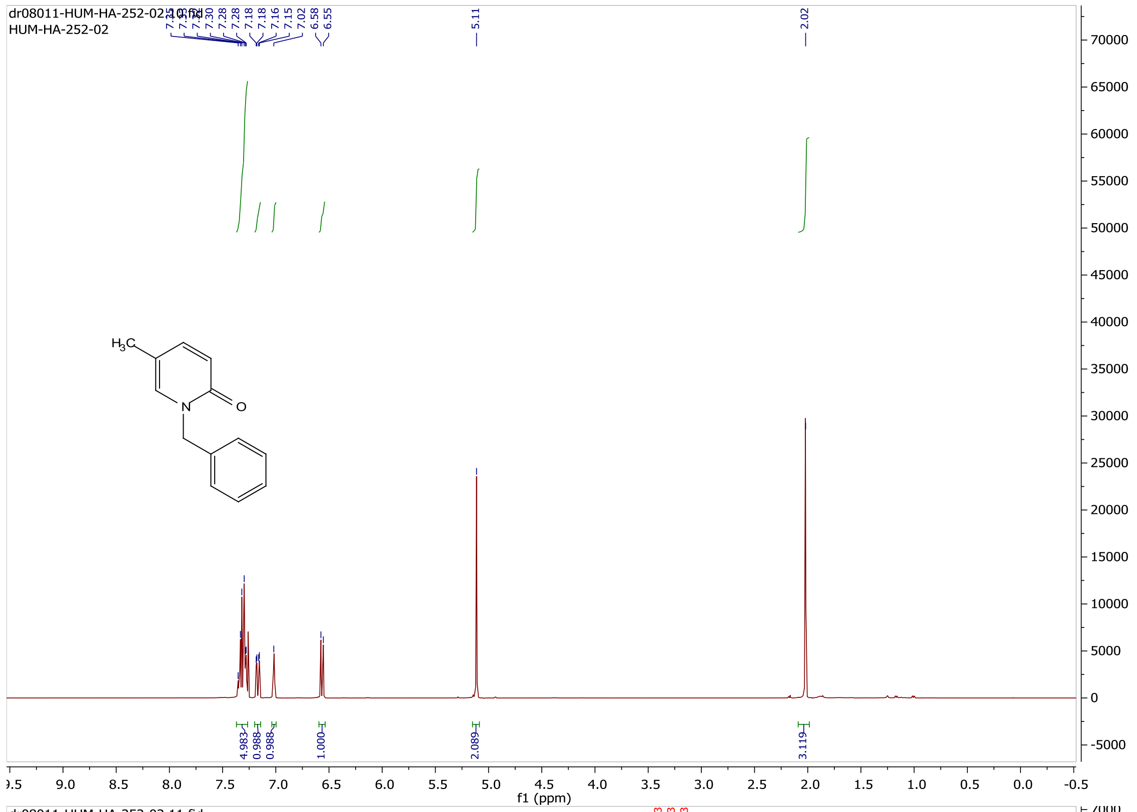

dr08011-HUM-HA-252-02.10.fid
HUM-HA-252-02
7.30
7.28
7.28
7.18
7.18
7.16
7.15
7.02
6.58
6.55
5.11
2.02
H_3C
N
O
4.983
0.988
0.988
1.000
2.089
3.119
f1 (ppm)

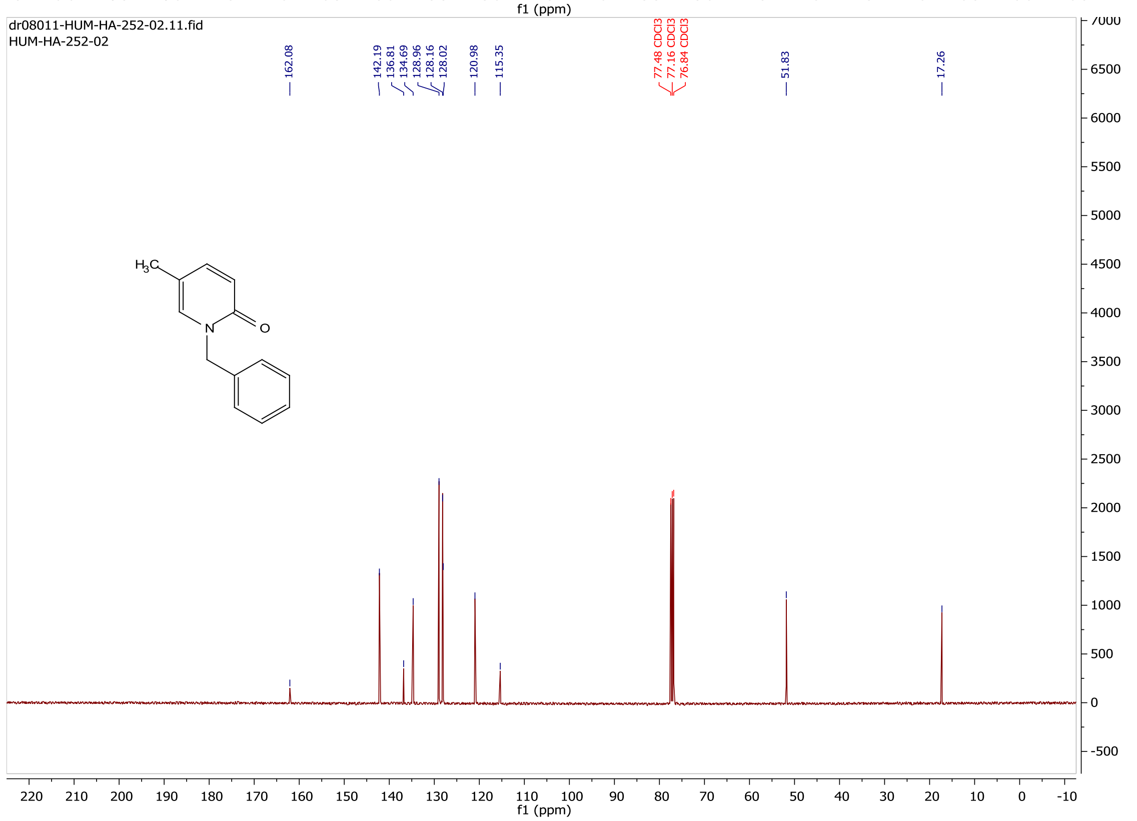

dr08011-HUM-HA-252-02.11.fid
HUM-HA-252-02
162.08
142.19
136.81
134.69
128.96
128.16
128.02
120.98
115.35
77.48 CDCl3
77.16 CDCl3
76.84 CDCl3
51.83
17.26
H_3C
N
O
f1 (ppm)

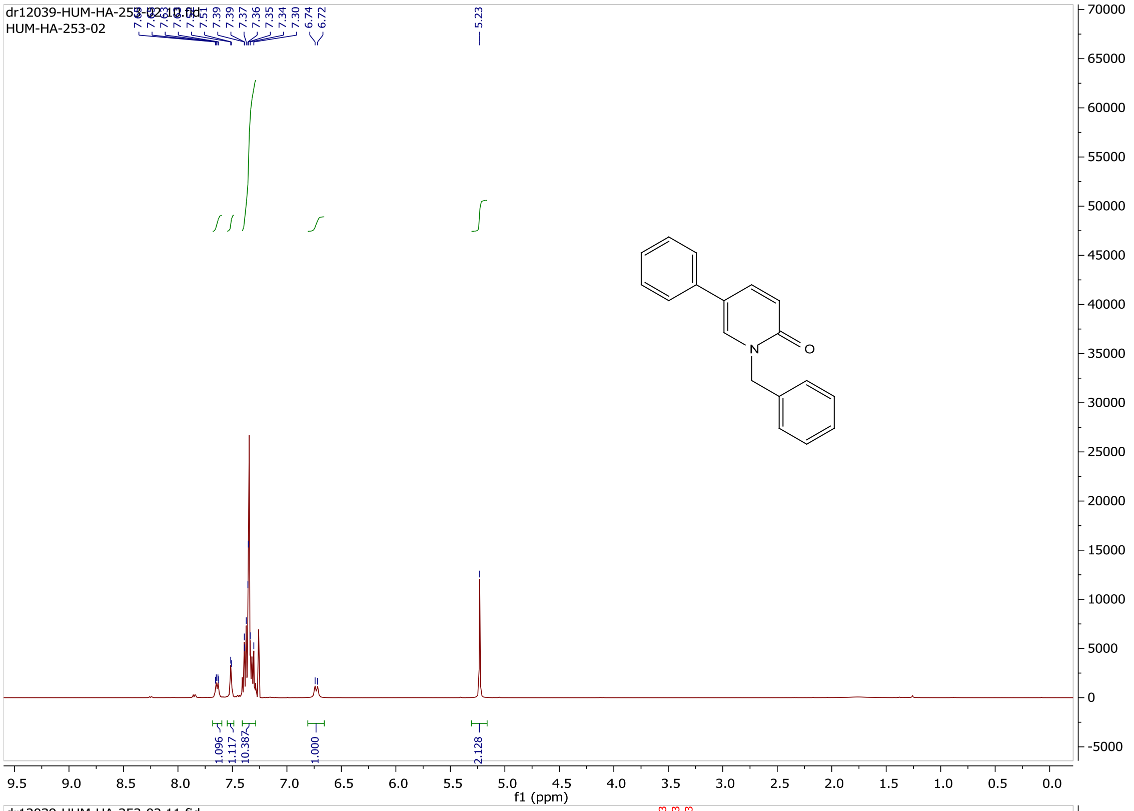
dr12039-HUM-HA-253-02.10.fid
HUM-HA-253-02
7.51
7.39
7.39
7.37
7.36
7.35
7.34
7.30
6.74
6.72
5.23
N
O
1.096
1.117
10.387
1.000
2.128
70000
65000
60000
55000
50000
45000
40000
35000
30000
25000
20000
15000
10000
5000
0
-5000
9.5 9.0 8.5 8.0 7.5 7.0 6.5 6.0 5.5 5.0 4.5 4.0 3.5 3.0 2.5 2.0 1.5 1.0 0.5 0.0
f1 (ppm)

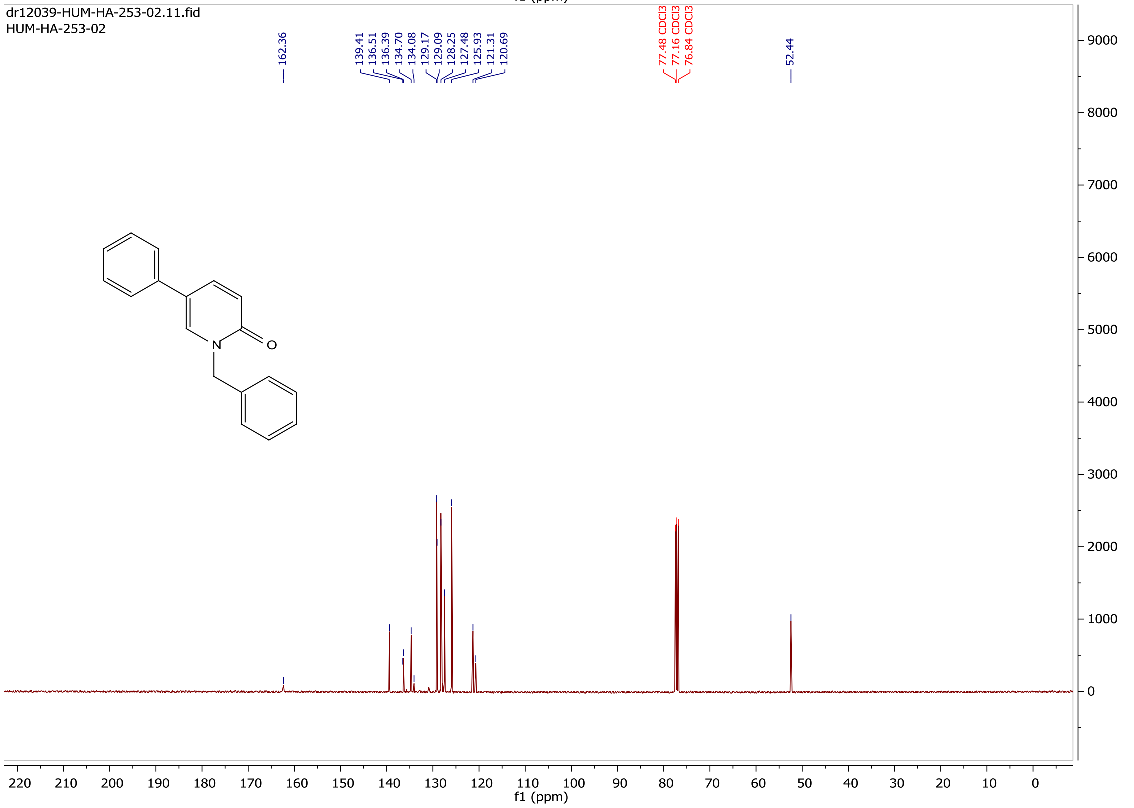
dr12039-HUM-HA-253-02.11.fid
HUM-HA-253-02
162.36
139.41
136.51
136.39
134.70
134.08
129.17
129.09
128.25
127.48
125.93
121.31
120.69
77.48 CDCl3
77.16 CDCl3
76.84 CDCl3
52.44
N
O
9000
8000
7000
6000
5000
4000
3000
2000
1000
0
220 210 200 190 180 170 160 150 140 130 120 110 100 90 80 70 60 50 40 30 20 10 0
f1 (ppm)

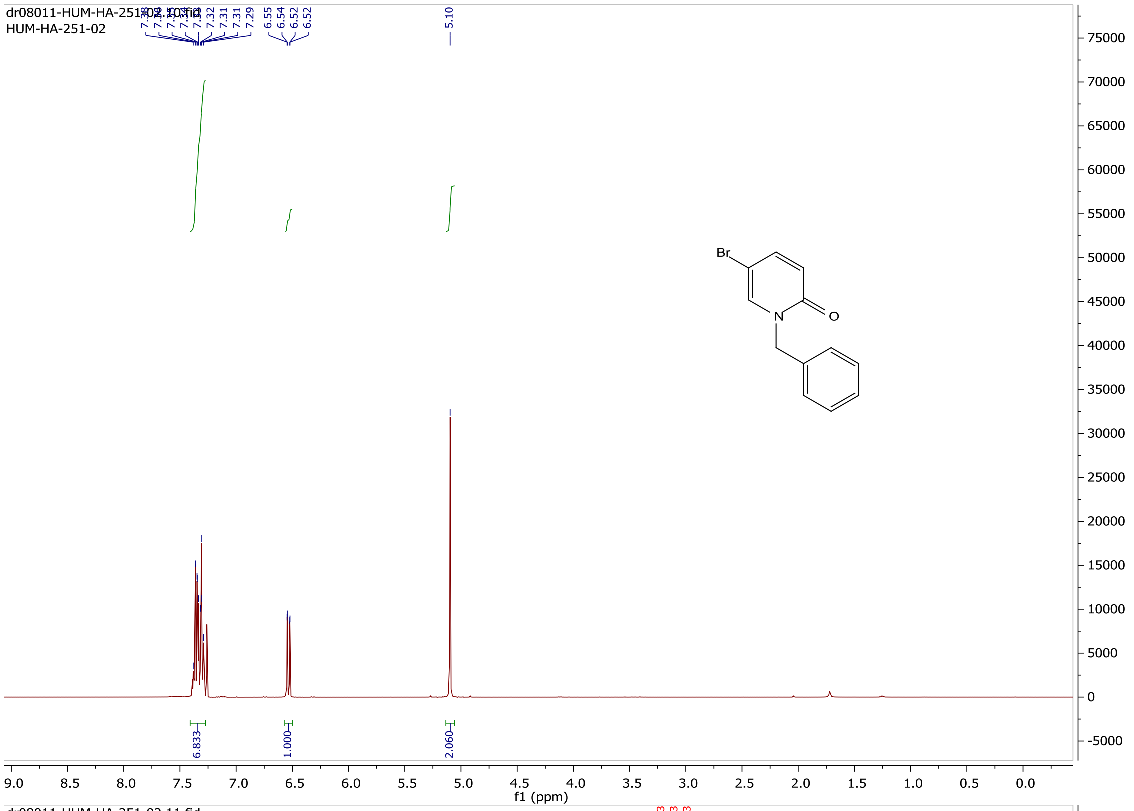
dr08011-HUM-HA-251-02.10.fid
HUM-HA-251-02
6.55
6.54
6.52
6.52
5.10
6.833
1.000
2.060
f1 (ppm)

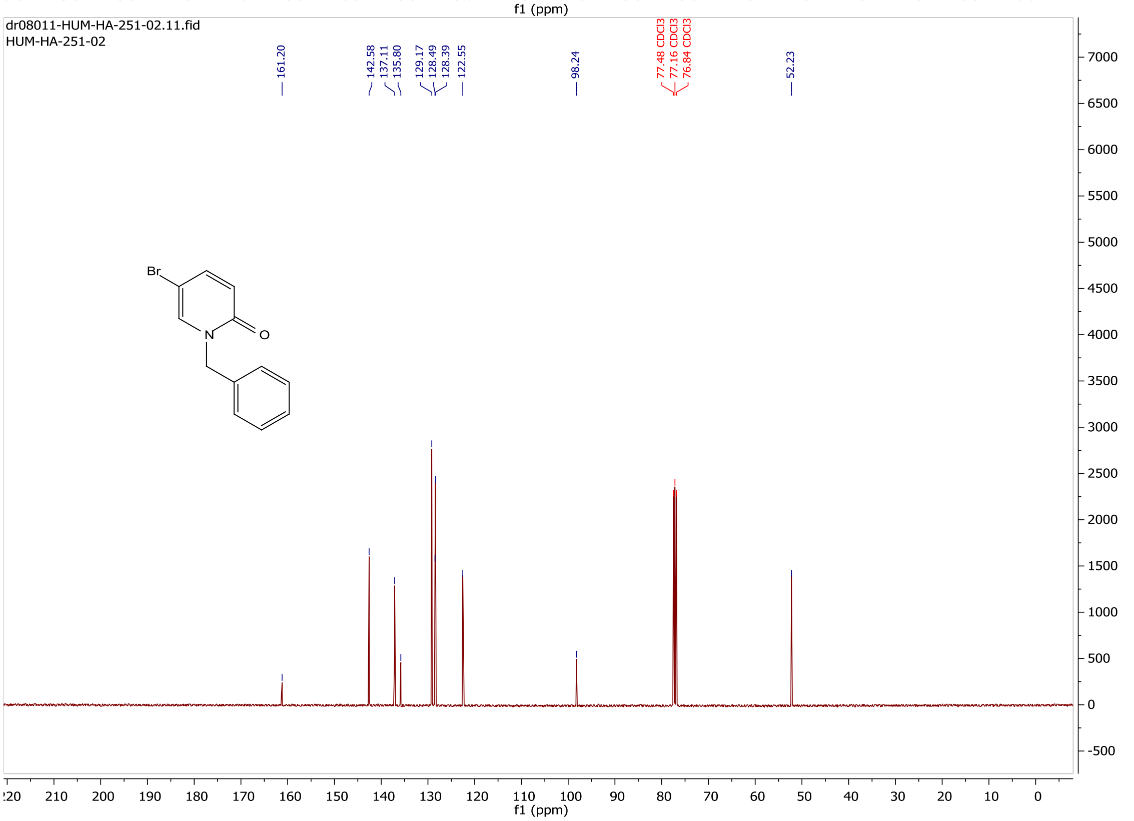
dr08011-HUM-HA-251-02.11.fid
HUM-HA-251-02
161.20
142.58
137.11
135.80
129.17
128.49
128.39
122.55
98.24
77.48 CDCl3
77.16 CDCl3
76.84 CDCl3
52.23
f1 (ppm)

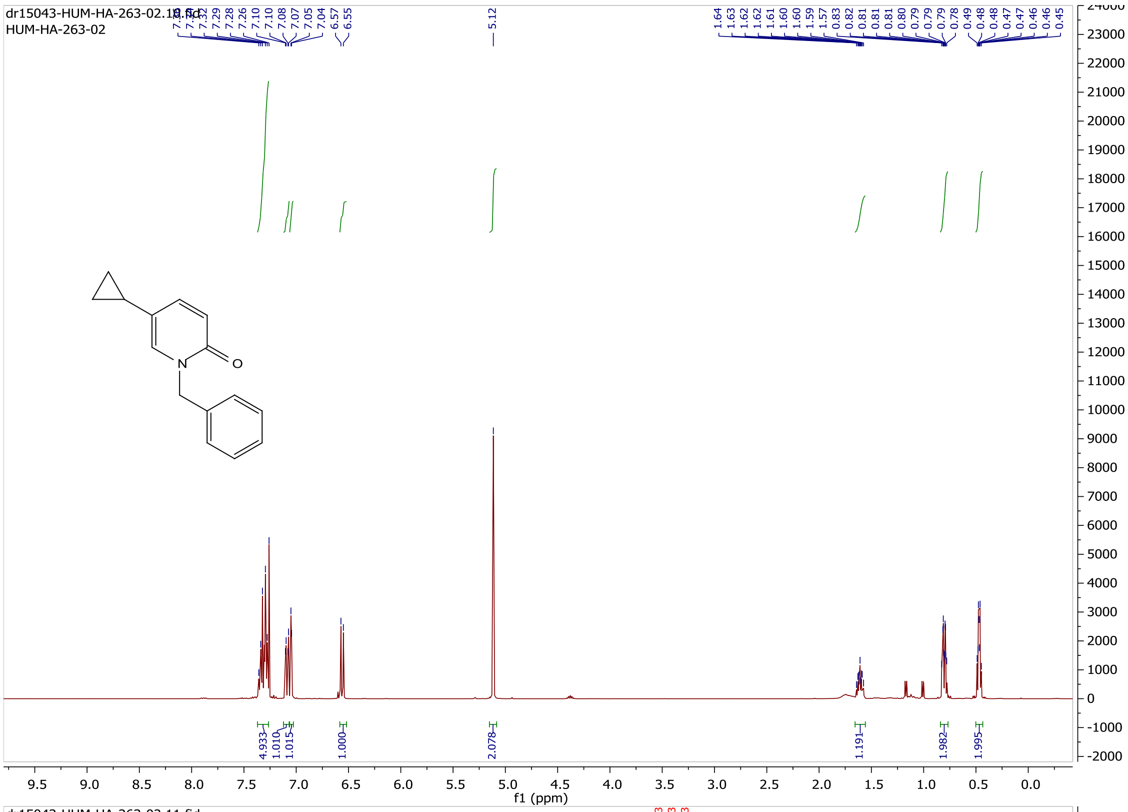
dr15043-HUM-HA-263-02.10.fid
HUM-HA-263-02
7.29
7.28
7.26
7.10
7.10
7.08
7.07
7.05
7.04
6.57
6.55
5.12
1.64
1.63
1.62
1.62
1.61
1.60
1.60
1.59
1.57
0.83
0.82
0.81
0.81
0.81
0.80
0.79
0.79
0.79
0.78
0.49
0.48
0.48
0.47
0.47
0.46
0.46
0.45
4.933
1.010
1.015
1.000
2.078
1.191
1.982
1.995
N
O
f1 (ppm)

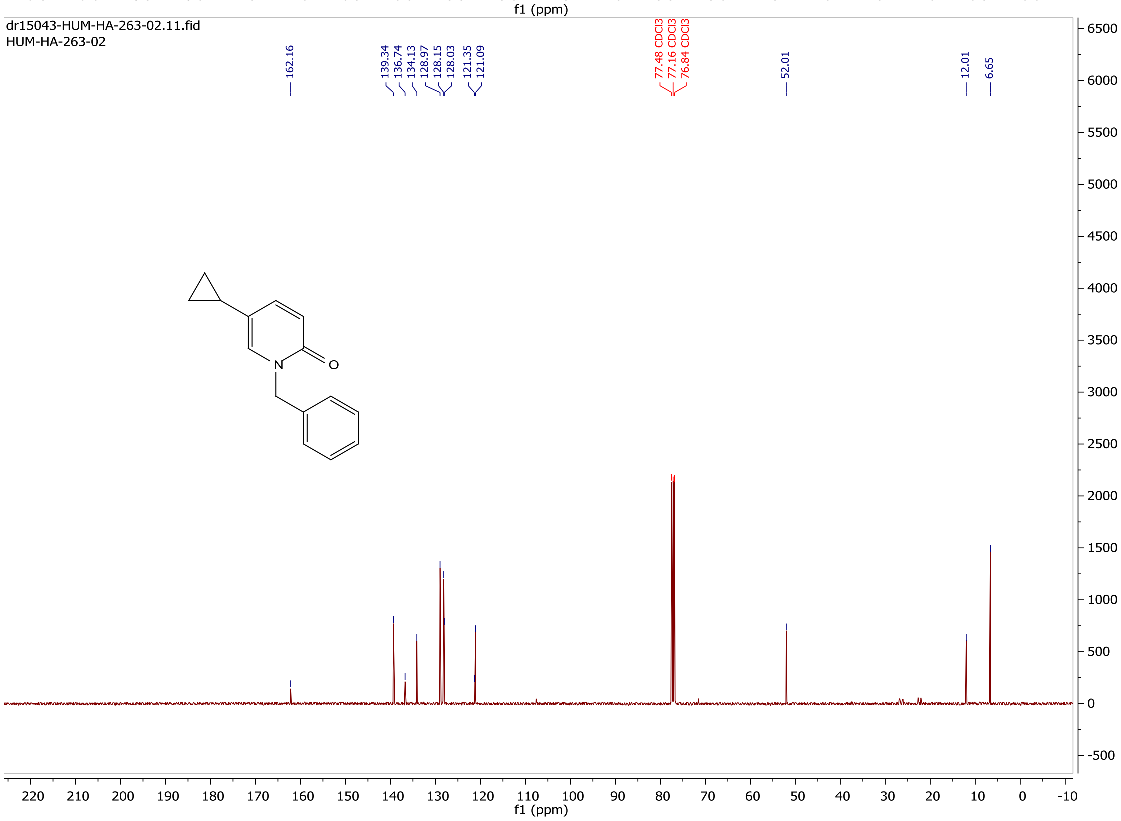
dr15043-HUM-HA-263-02.11.fid
HUM-HA-263-02
162.16
139.34
136.74
134.13
128.97
128.15
128.03
121.35
121.09
77.48 CDCl3
77.16 CDCl3
76.84 CDCl3
52.01
12.01
6.65
N
O
f1 (ppm)

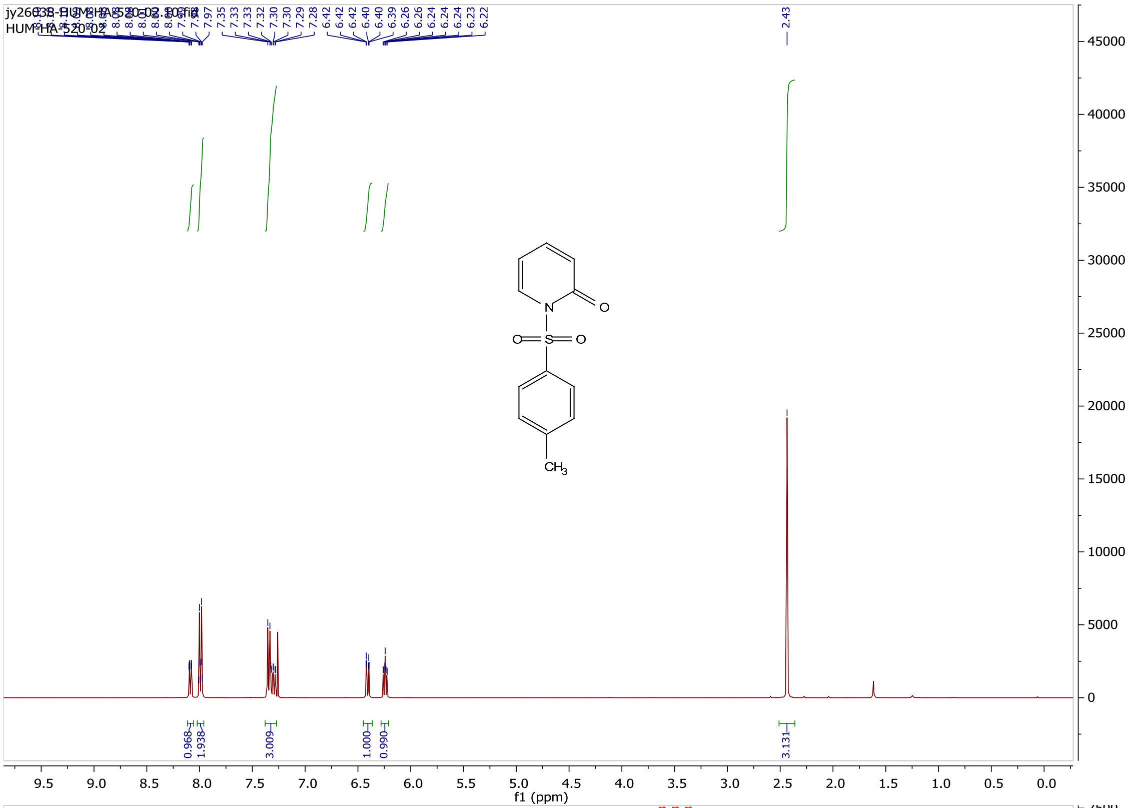
jy26038-HUM-HA-520-02.10.fid
HUM-HA-520-02
2.43
0.968
1.938
3.009
1.000
0.990
3.131
f1 (ppm)

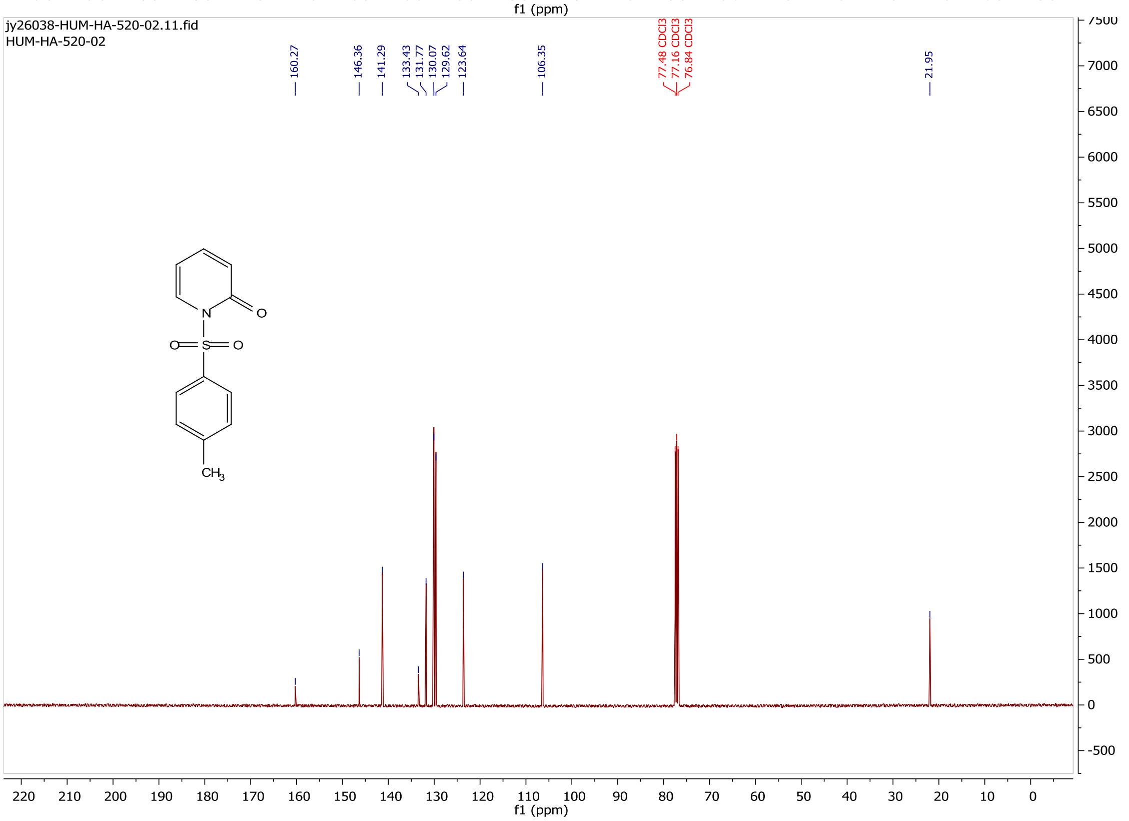
jy26038-HUM-HA-520-02.11.fid
HUM-HA-520-02
160.27
146.36
141.29
133.43
131.77
130.07
129.62
123.64
106.35
77.48 CDCl3
77.16 CDCl3
76.84 CDCl3
21.95
f1 (ppm)

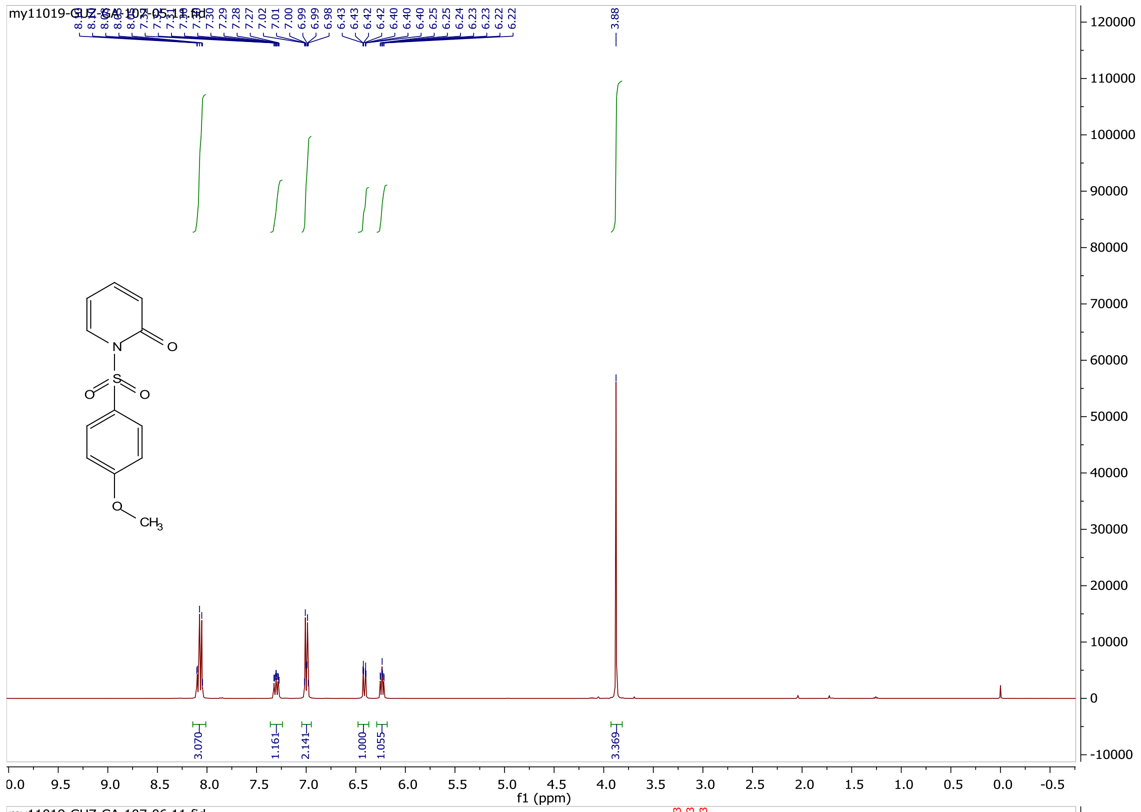

my11019-GUZ-GA-107-05.11.fid
3.88
3.070
1.161
2.141
1.000
1.055
3.369
f1 (ppm)

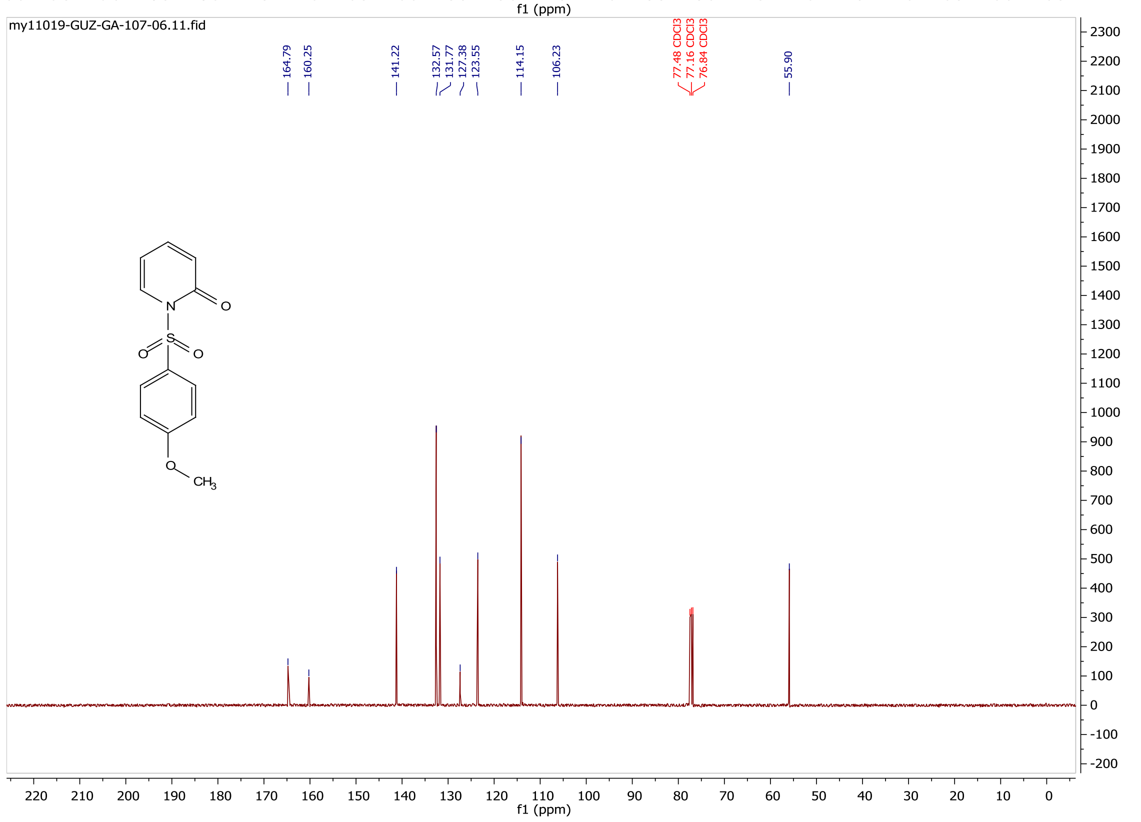

my11019-GUZ-GA-107-06.11.fid
164.79
160.25
141.22
132.57
131.77
127.38
123.55
114.15
106.23
77.48 CDCl3
77.16 CDCl3
76.84 CDCl3
55.90
f1 (ppm)

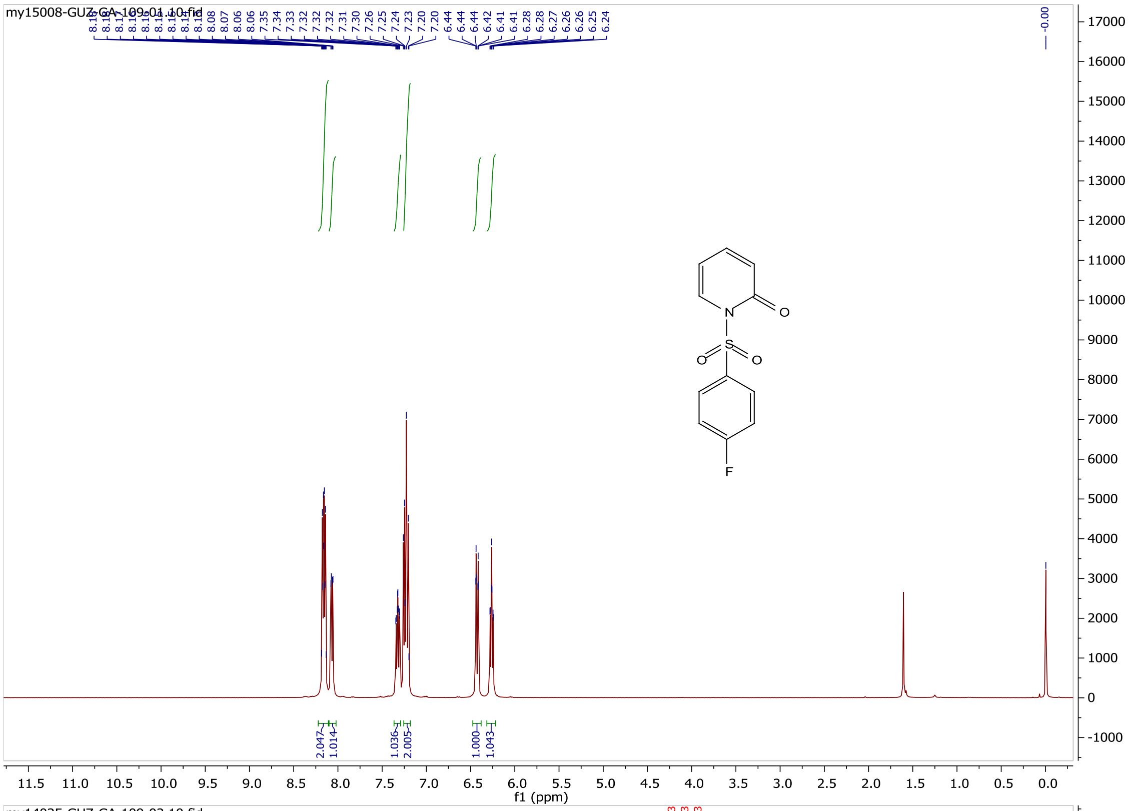
my15008-GUZ-GA-109-01.10.fid
8.08
8.07
8.06
8.06
7.35
7.34
7.33
7.32
7.32
7.32
7.31
7.30
7.26
7.25
7.24
7.23
7.20
6.44
6.44
6.42
6.41
6.41
6.28
6.28
6.27
6.26
6.26
6.25
6.24
-0.00
2.047
1.014
1.036
2.005
1.000
1.043
N
O
S
O
O
F
f1 (ppm)

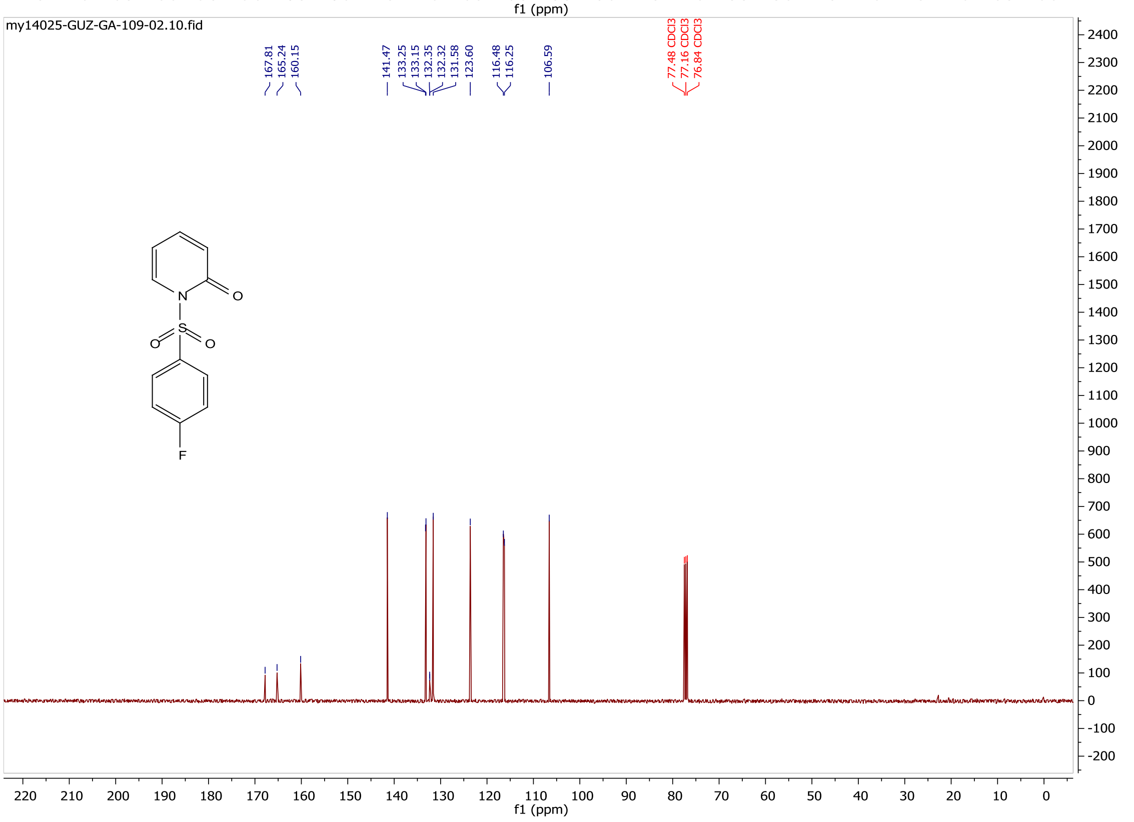
my14025-GUZ-GA-109-02.10.fid
167.81
165.24
160.15
141.47
133.25
133.15
132.35
132.32
131.58
123.60
116.48
116.25
106.59
77.48 CDCl3
77.16 CDCl3
76.84 CDCl3
N
O
S
O
O
F
f1 (ppm)

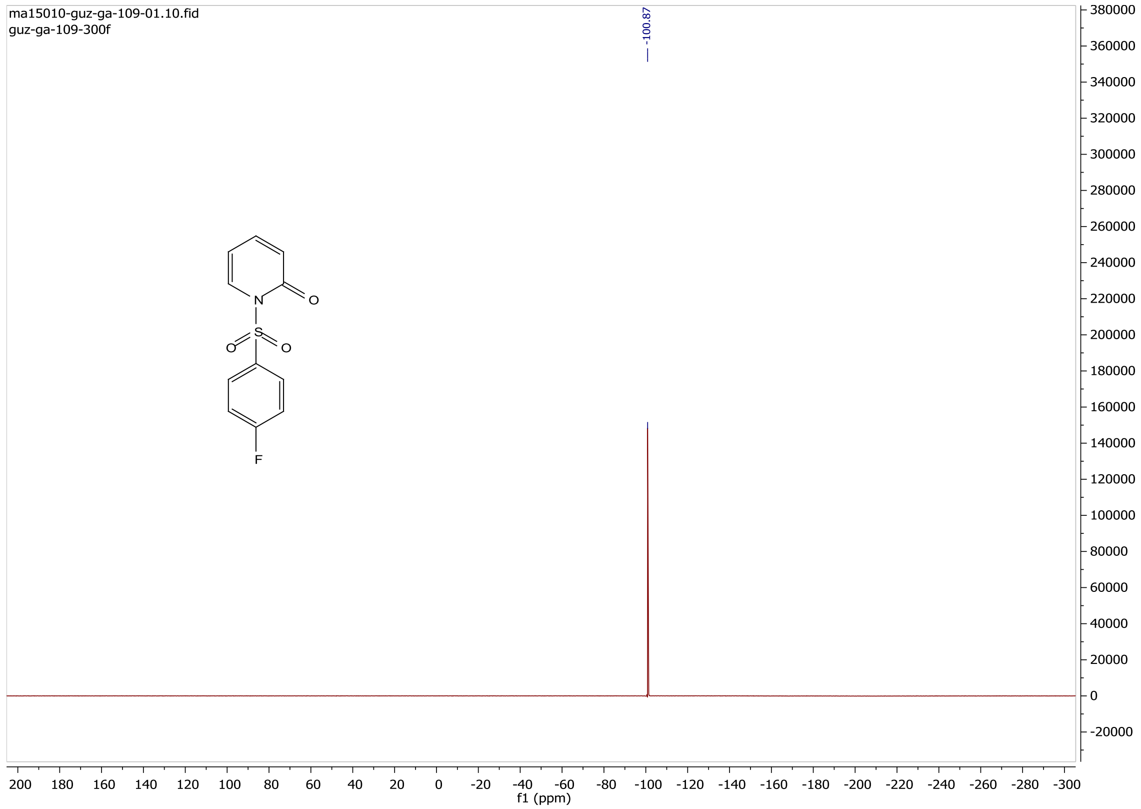

ma15010-guz-ga-109-01.10.fid
guz-ga-109-300f
-100.87
f1 (ppm)

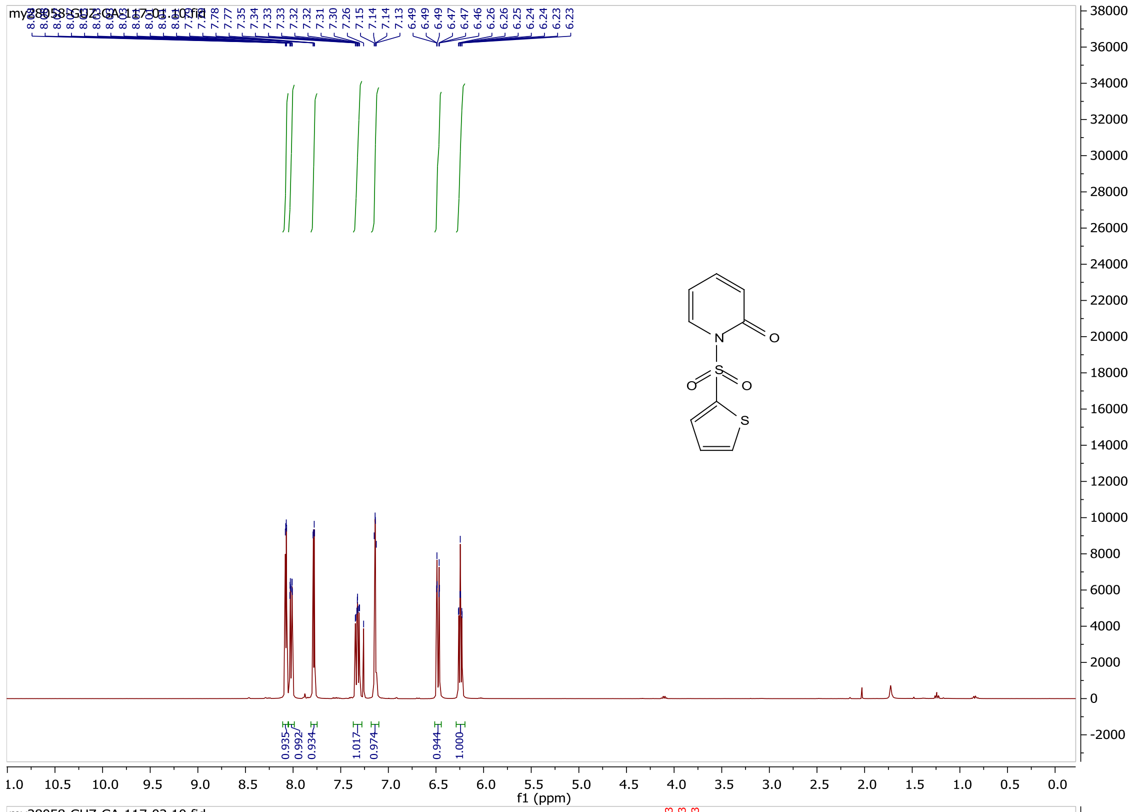
7.35
7.34
7.33
7.33
7.32
7.32
7.31
7.30
7.26
7.15
7.14
7.14
7.13
6.49
6.49
6.47
6.47
6.46
6.26
6.25
6.24
6.24
6.23
6.23
0.935
0.992
0.934
1.017
0.974
0.944
1.000
f1 (ppm)

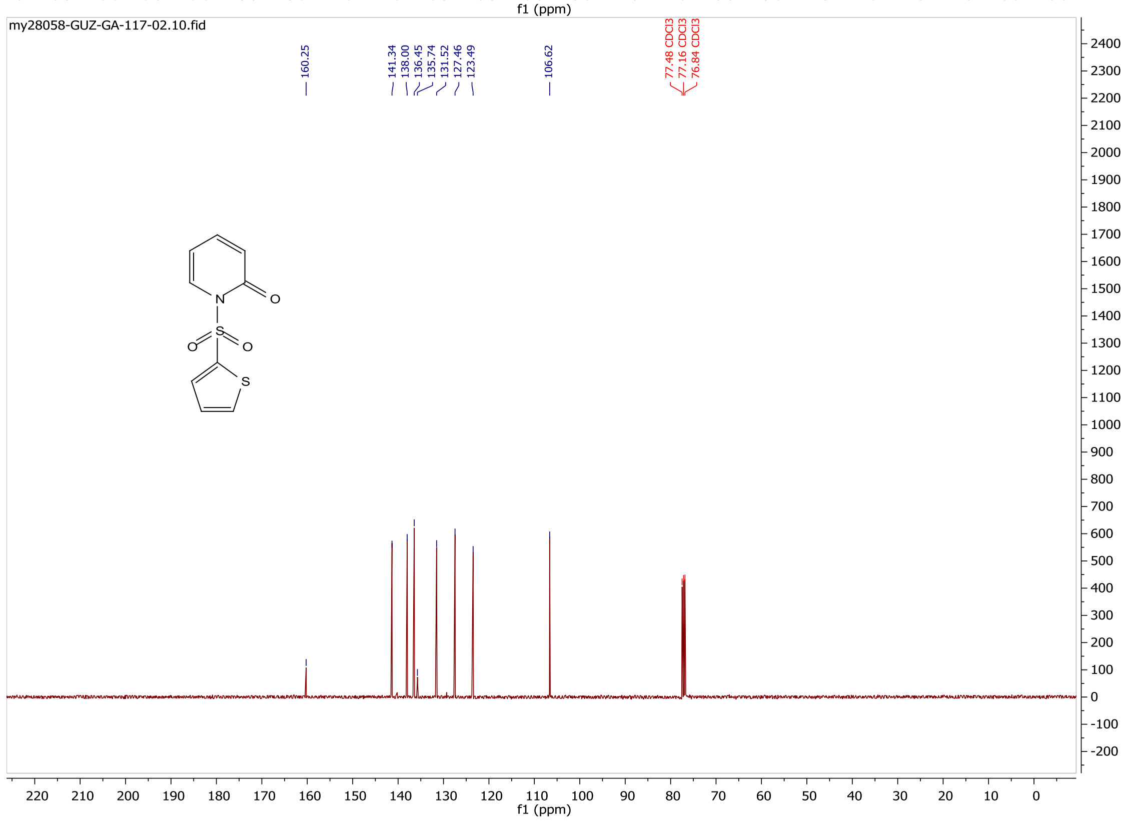
my28058-GUZ-GA-117-02.10.fid
160.25
141.34
138.00
136.45
135.74
131.52
127.46
123.49
106.62
77.48 CDCl3
77.16 CDCl3
76.84 CDCl3
f1 (ppm)

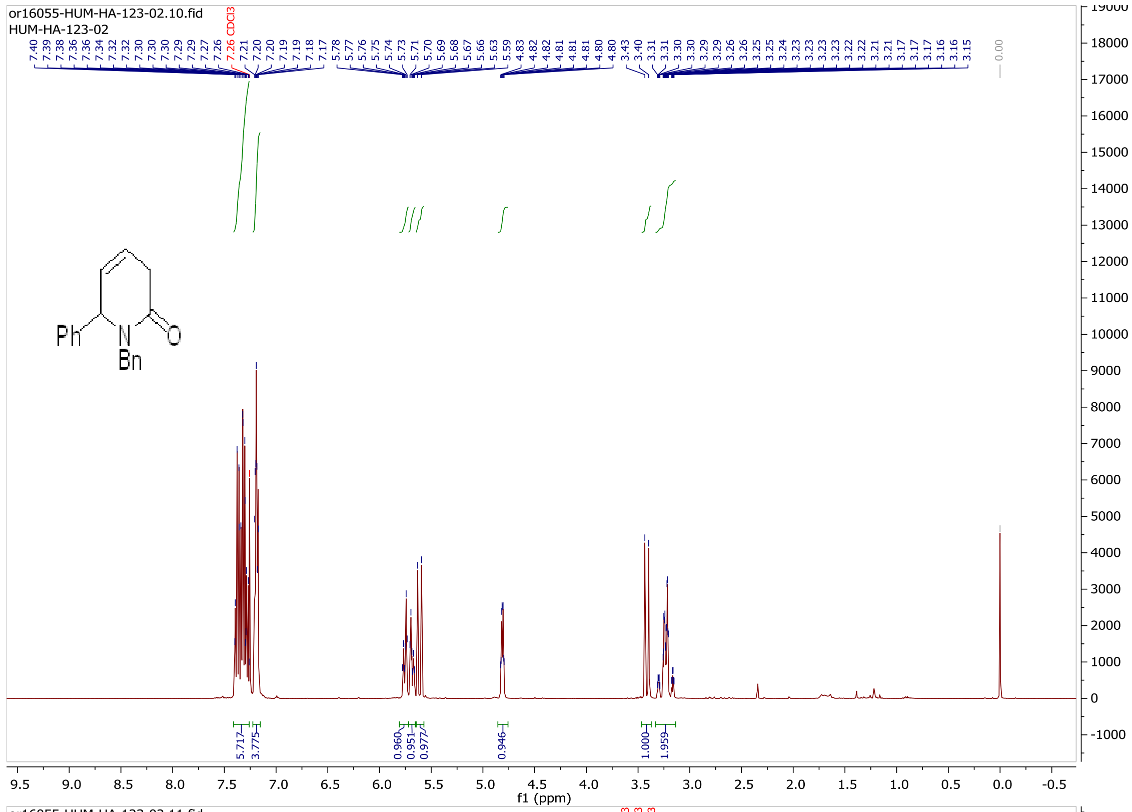
or16055-HUM-HA-123-02.10.fid
HUM-HA-123-02
7.40 7.39 7.38 7.36 7.36 7.34 7.32 7.32 7.32 7.30 7.30 7.30 7.29 7.29 7.27 7.26 CDCl3 7.21 7.20 7.20 7.19 7.19 7.18 7.17 5.78 5.77 5.76 5.75 5.74 5.73 5.71 5.70 5.69 5.68 5.67 5.66 5.63 5.59 4.83 4.82 4.81 4.81 4.81 4.80 3.43 3.40 3.31 3.31 3.30 3.30 3.29 3.29 3.26 3.26 3.25 3.25 3.24 3.23 3.23 3.23 3.23 3.22 3.22 3.21 3.21 3.17 3.17 3.17 3.16 3.16 3.15 0.00
Ph
N
O
Bn
5.717
3.775
0.960
0.951
0.977
0.946
1.000
1.959
f1 (ppm)
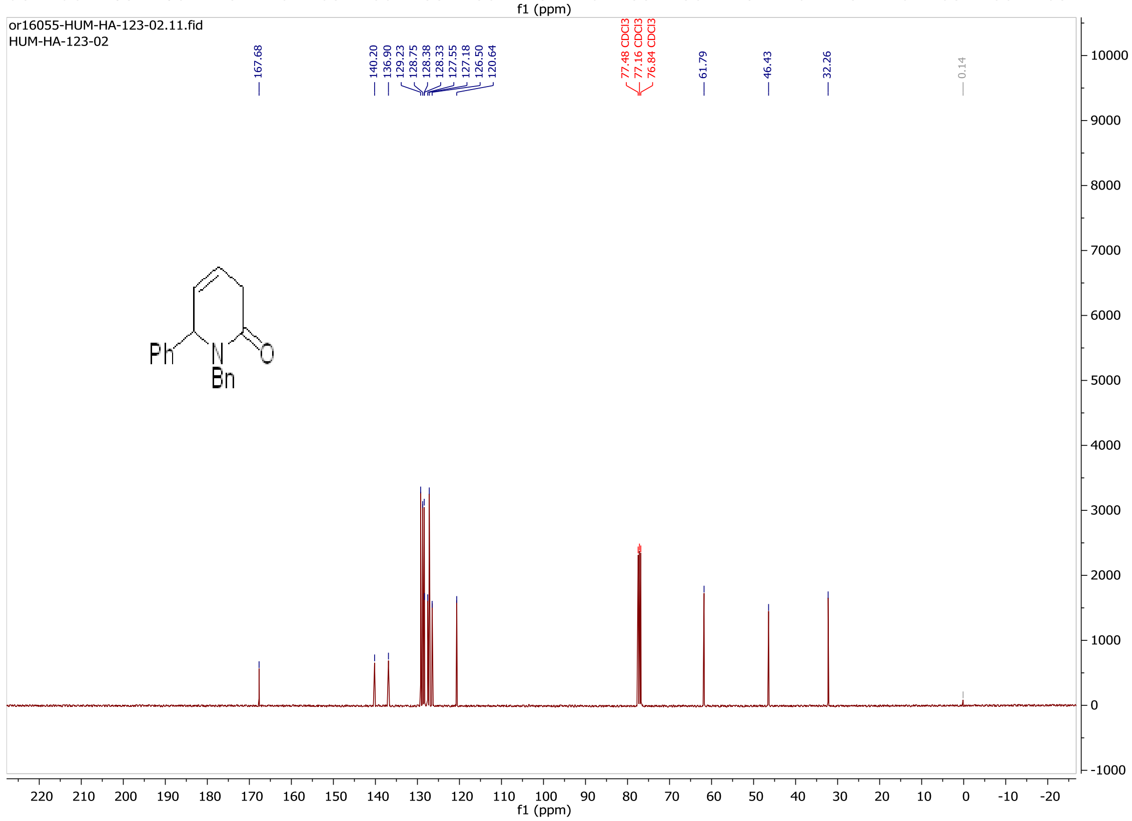
or16055-HUM-HA-123-02.11.fid
HUM-HA-123-02
167.68
140.20 136.90 129.23 128.75 128.38 128.33 127.55 127.18 126.50 120.64
77.48 CDCl3 77.16 CDCl3 76.84 CDCl3
61.79
46.43
32.26
0.14
Ph
N
O
Bn
f1 (ppm)

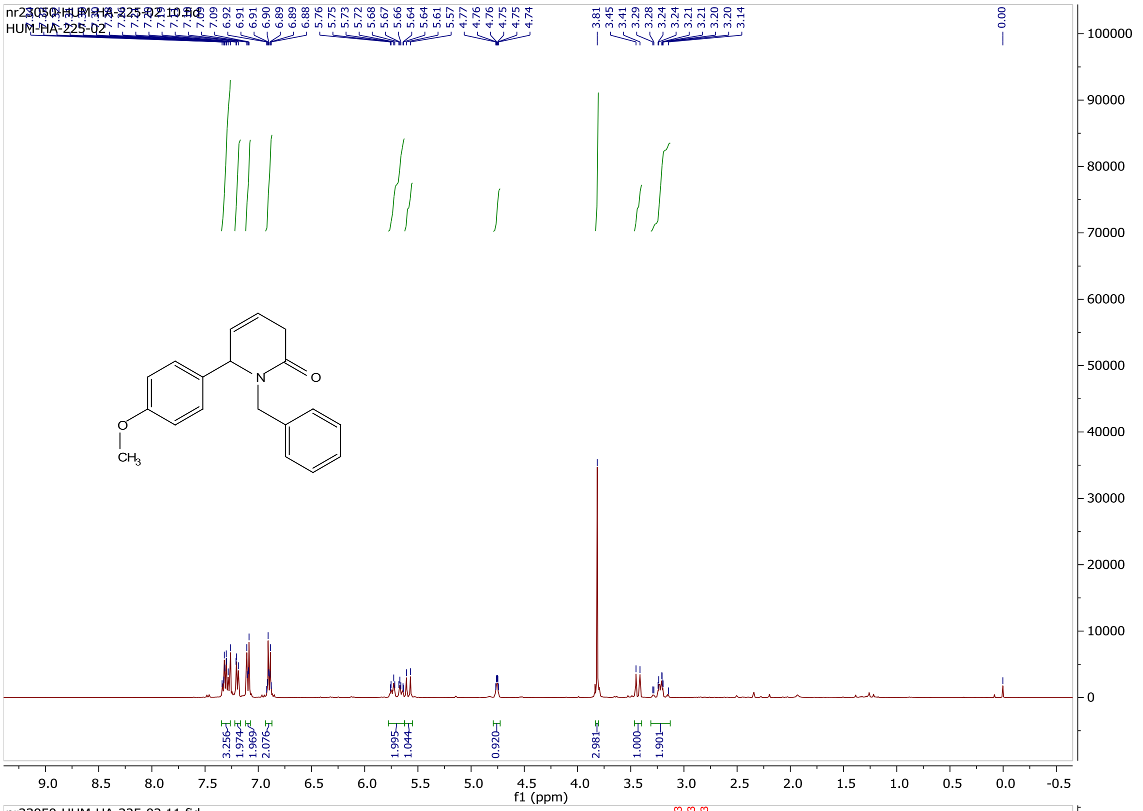
nr23050-HUM-HA-225-02.10.fid
HUM-HA-225-02
7.09
6.92
6.91
6.91
6.90
6.89
6.89
6.88
5.76
5.75
5.73
5.72
5.68
5.67
5.66
5.64
5.64
5.61
5.57
4.77
4.76
4.76
4.75
4.75
4.74
3.81
3.45
3.41
3.29
3.28
3.24
3.24
3.21
3.21
3.20
3.20
3.14
0.00
3.256
1.974
1.969
2.076
1.995
1.044
0.920
2.981
1.000
1.901
N
O
CH3
f1 (ppm)

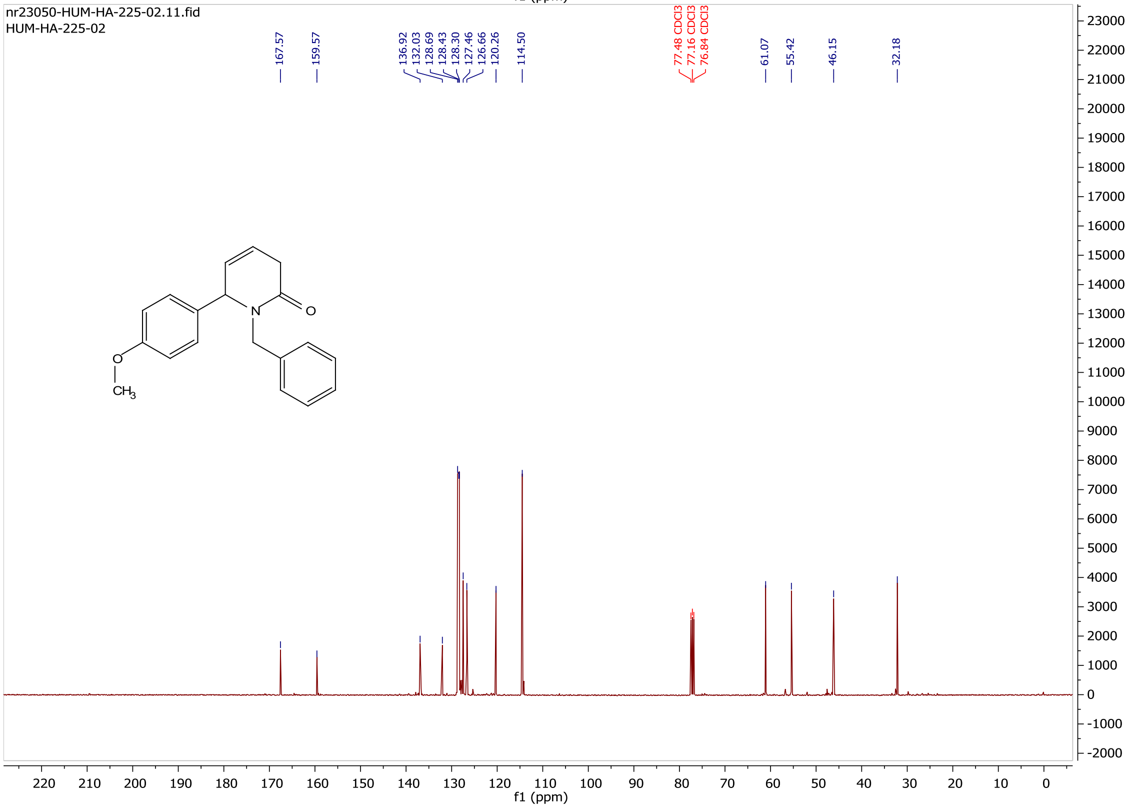
nr23050-HUM-HA-225-02.11.fid
HUM-HA-225-02
167.57
159.57
136.92
132.03
128.69
128.43
128.30
127.46
126.66
120.26
114.50
77.48 CDCl3
77.16 CDCl3
76.84 CDCl3
61.07
55.42
46.15
32.18
N
O
CH3
f1 (ppm)

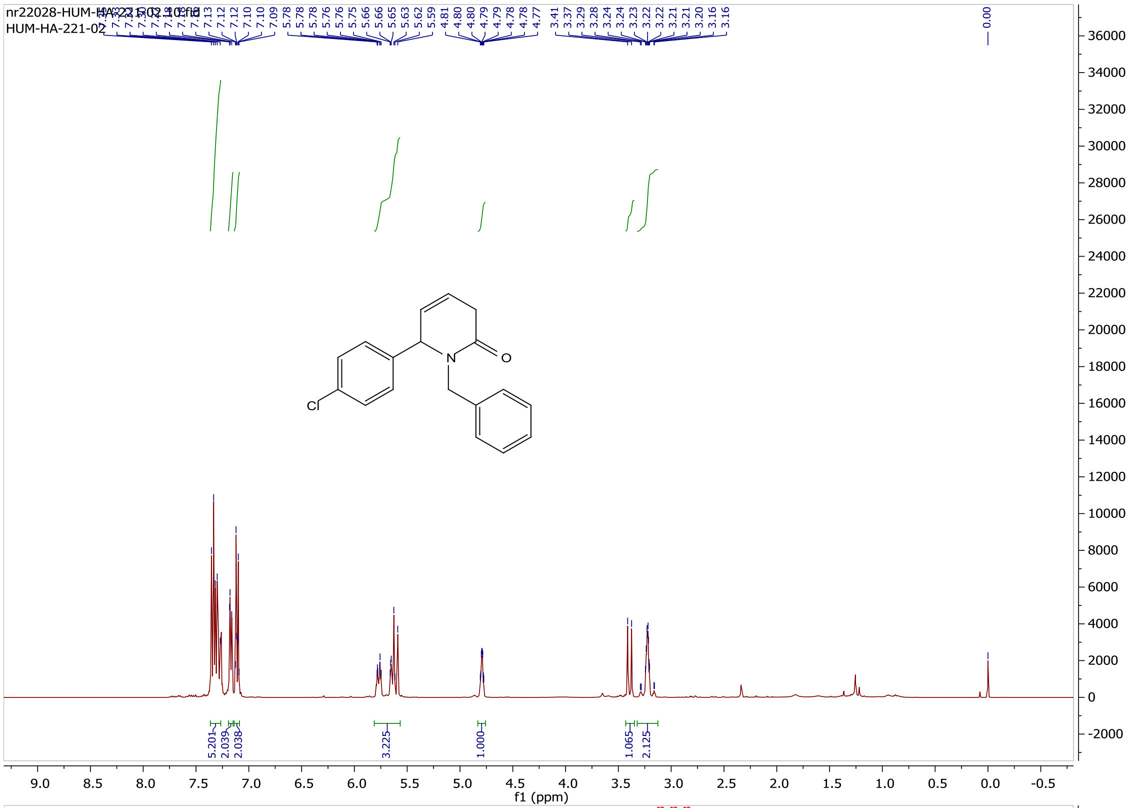
HUM-HA-221-02
7.13 7.12 7.12 7.10 7.10 7.09 5.78 5.78 5.78 5.76 5.75 5.66 5.66 5.65 5.63 5.62 5.59 4.81 4.80 4.80 4.79 4.79 4.78 4.78 4.77 3.41 3.37 3.29 3.28 3.24 3.24 3.23 3.22 3.22 3.21 3.21 3.20 3.16 3.16
0.00
5.201 2.039 2.038 3.225 1.000 1.065 2.125
Cl
N
O
f1 (ppm)

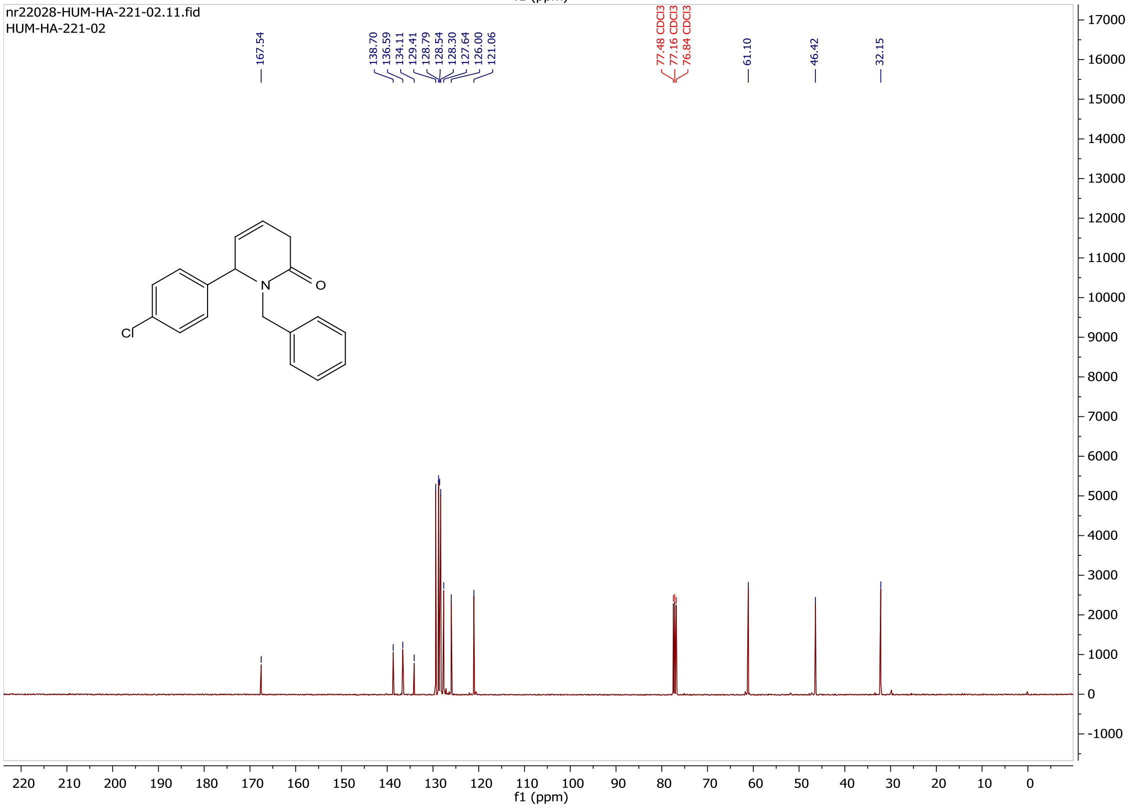
nr22028-HUM-HA-221-02.11.fid
HUM-HA-221-02
167.54
138.70 136.59 134.11 129.41 128.79 128.54 128.30 127.64 126.00 121.06
77.48 CDCl3 77.16 CDCl3 76.84 CDCl3
61.10
46.42
32.15
Cl
N
O
f1 (ppm)

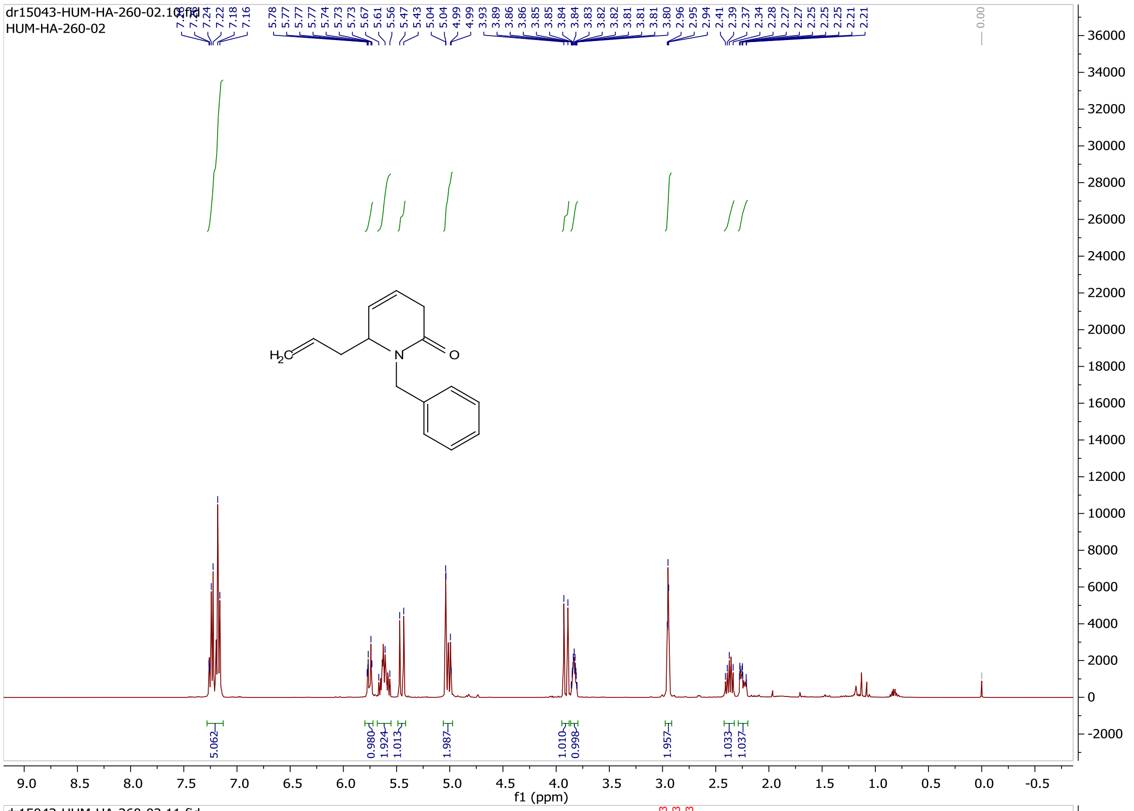
dr15043-HUM-HA-260-02.10.fid
HUM-HA-260-02
H2C
N
O
f1 (ppm)

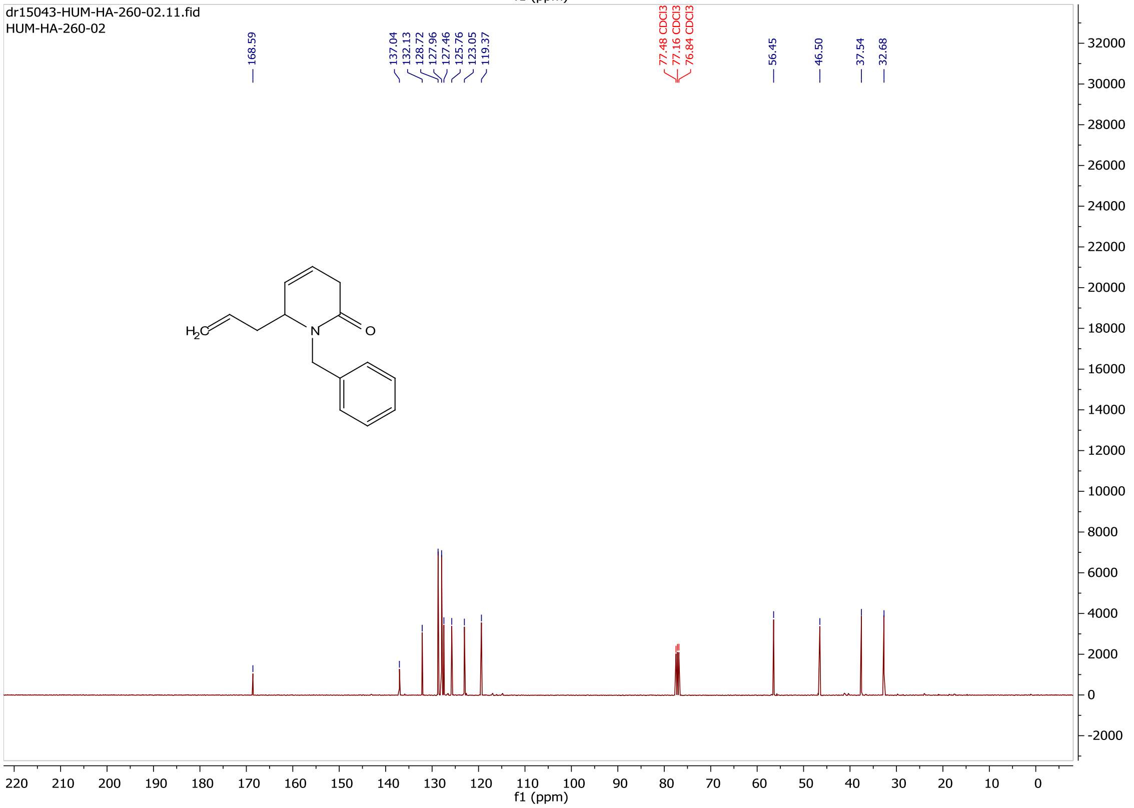
dr15043-HUM-HA-260-02.11.fid
HUM-HA-260-02
168.59
137.04
132.13
128.72
127.96
127.46
125.76
123.05
119.37
77.48 CDCl3
77.16 CDCl3
76.84 CDCl3
56.45
46.50
37.54
32.68
H2C
N
O
f1 (ppm)

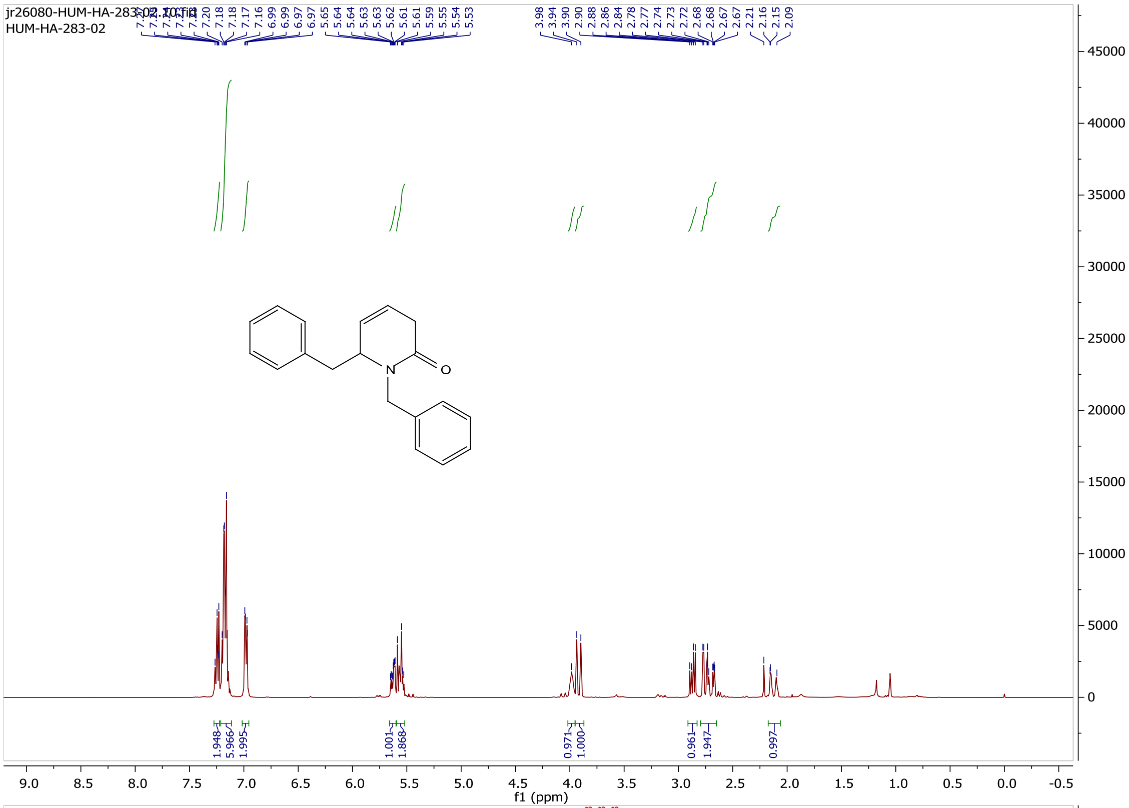
HUM-HA-283-02
7.18 7.18 7.17 7.16 6.99 6.99 6.97 6.97 5.65 5.64 5.64 5.63 5.63 5.62 5.61 5.61 5.59 5.55 5.54 5.53
3.98 3.94 3.90 2.90 2.88 2.86 2.84 2.78 2.77 2.74 2.73 2.72 2.68 2.68 2.67 2.67 2.21 2.16 2.15 2.09
N
O
1.948 5.966 1.995 1.001 1.868 0.971 1.000 0.961 1.947 0.997
f1 (ppm)

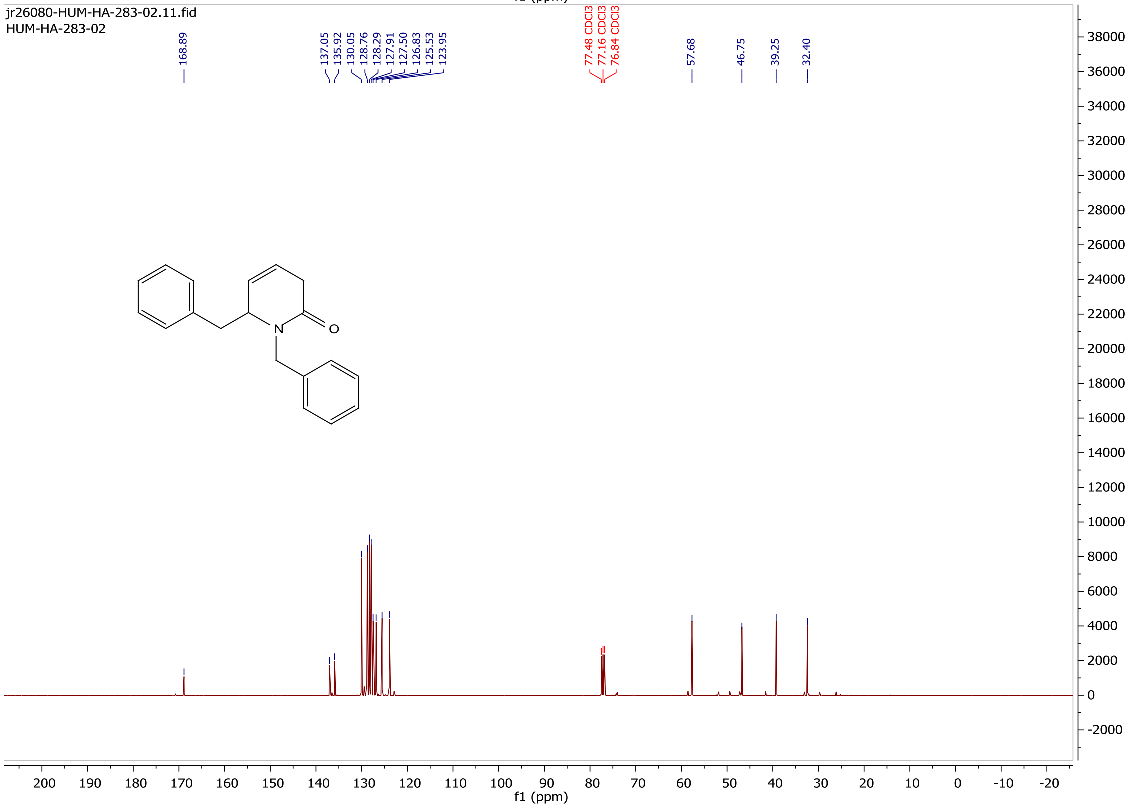
jr26080-HUM-HA-283-02.11.fid
HUM-HA-283-02
168.89
137.05 135.92 130.05 128.76 128.29 127.91 127.50 126.83 125.53 123.95
77.48 CDCl3 77.16 CDCl3 76.84 CDCl3
57.68 46.75 39.25 32.40
N
O
f1 (ppm)

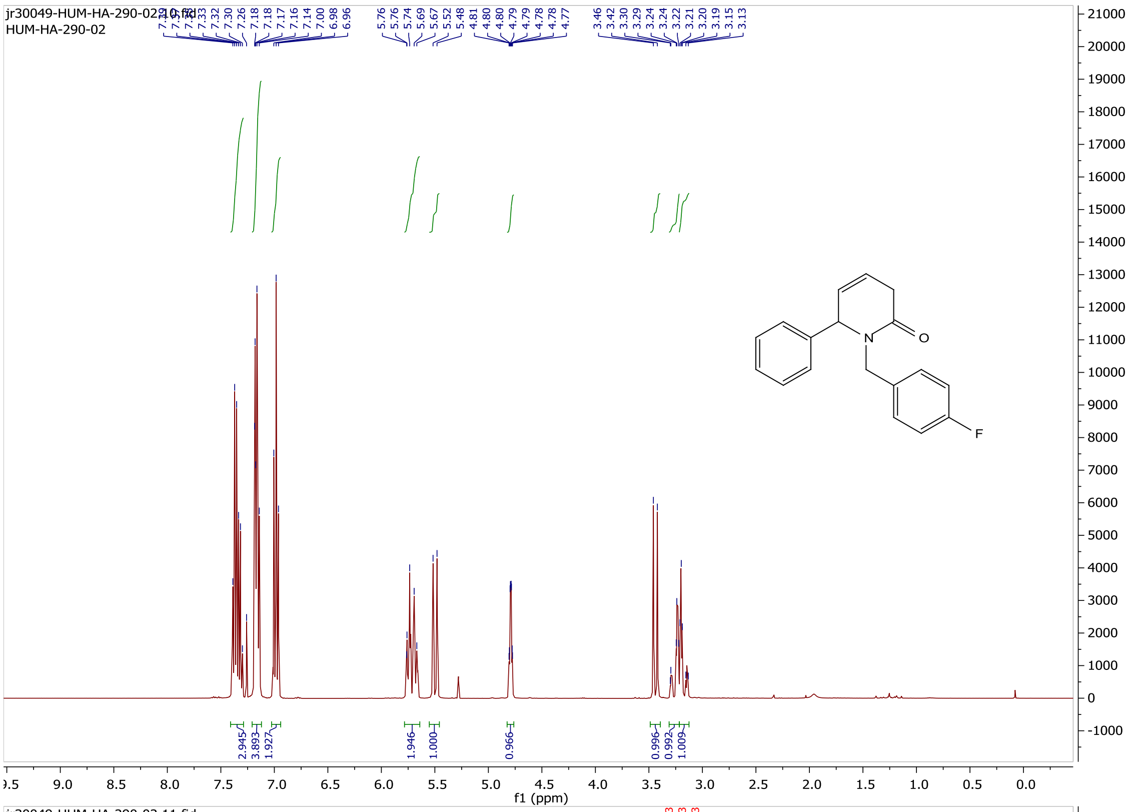
jr30049-HUM-HA-290-02.10.fid
HUM-HA-290-02
f1 (ppm)
N
O
F

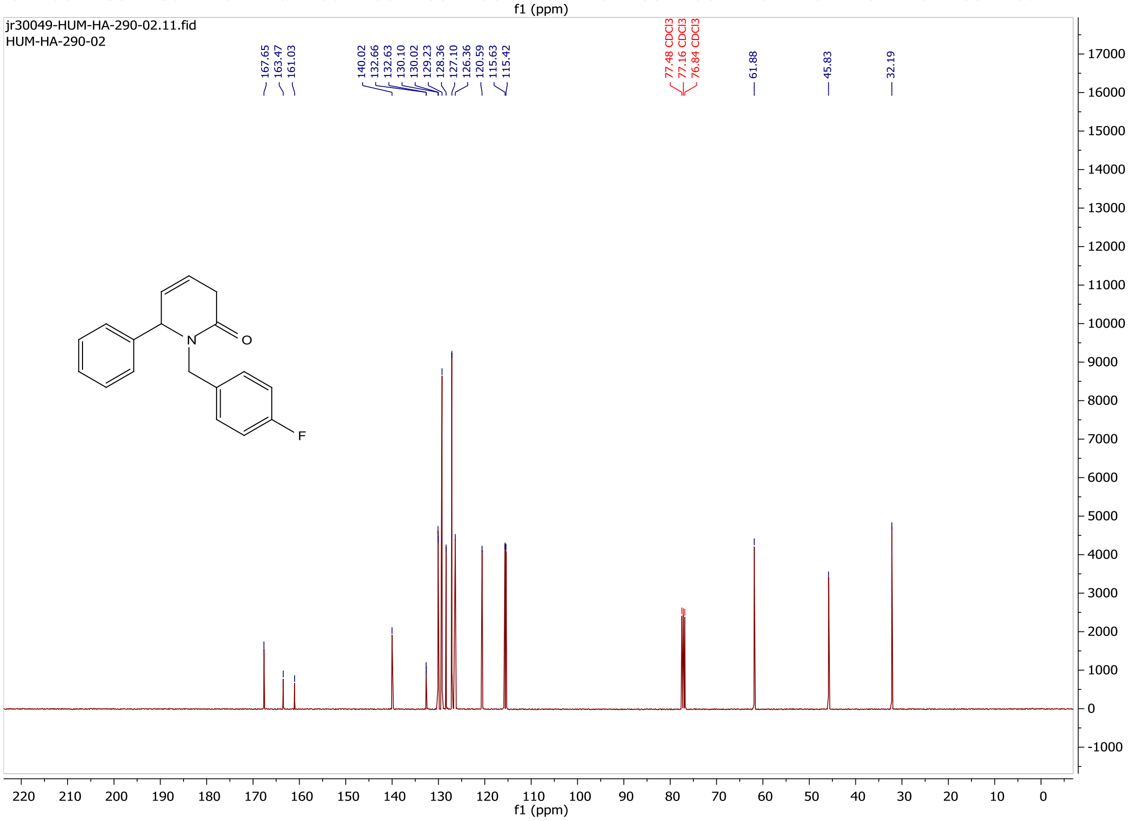
jr30049-HUM-HA-290-02.11.fid
HUM-HA-290-02
167.65
163.47
161.03
140.02
132.66
132.63
130.10
130.02
129.23
128.36
127.10
126.36
120.59
115.63
115.42
77.48 CDCl3
77.16 CDCl3
76.84 CDCl3
61.88
45.83
32.19
f1 (ppm)
N
O
F

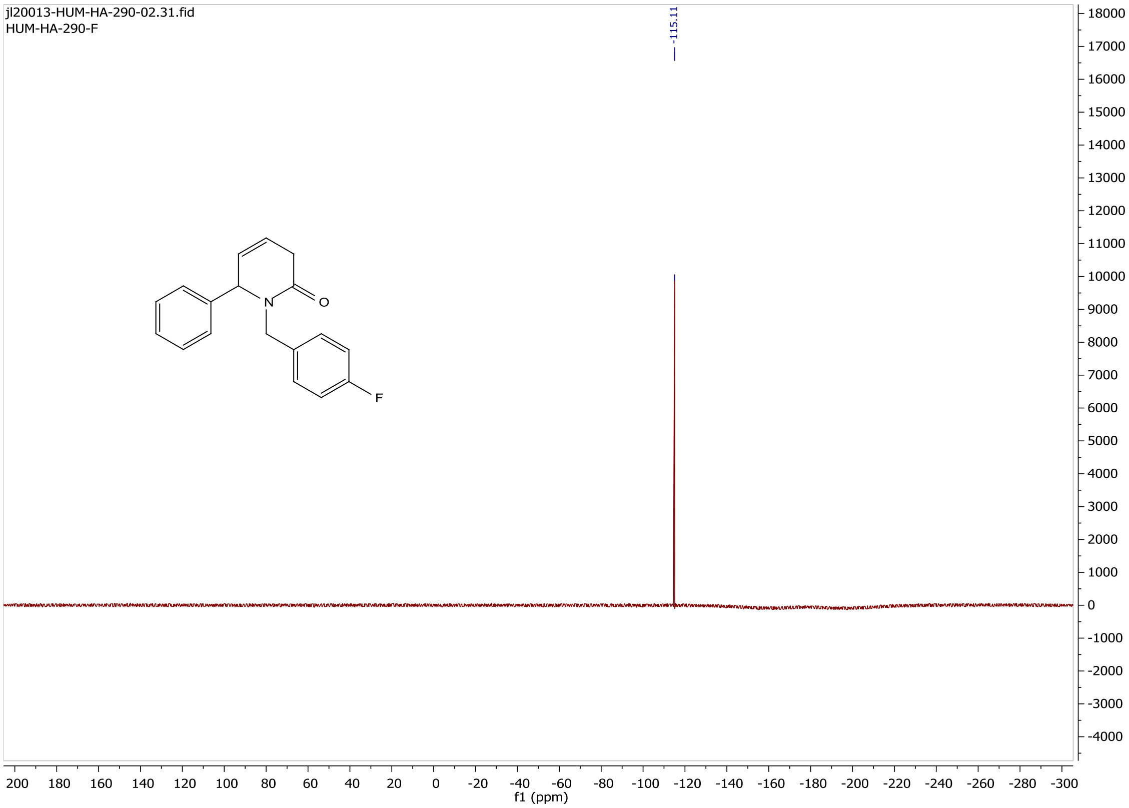
jl20013-HUM-HA-290-02.31.fid
HUM-HA-290-F
-115.11
N
O
F
f1 (ppm)

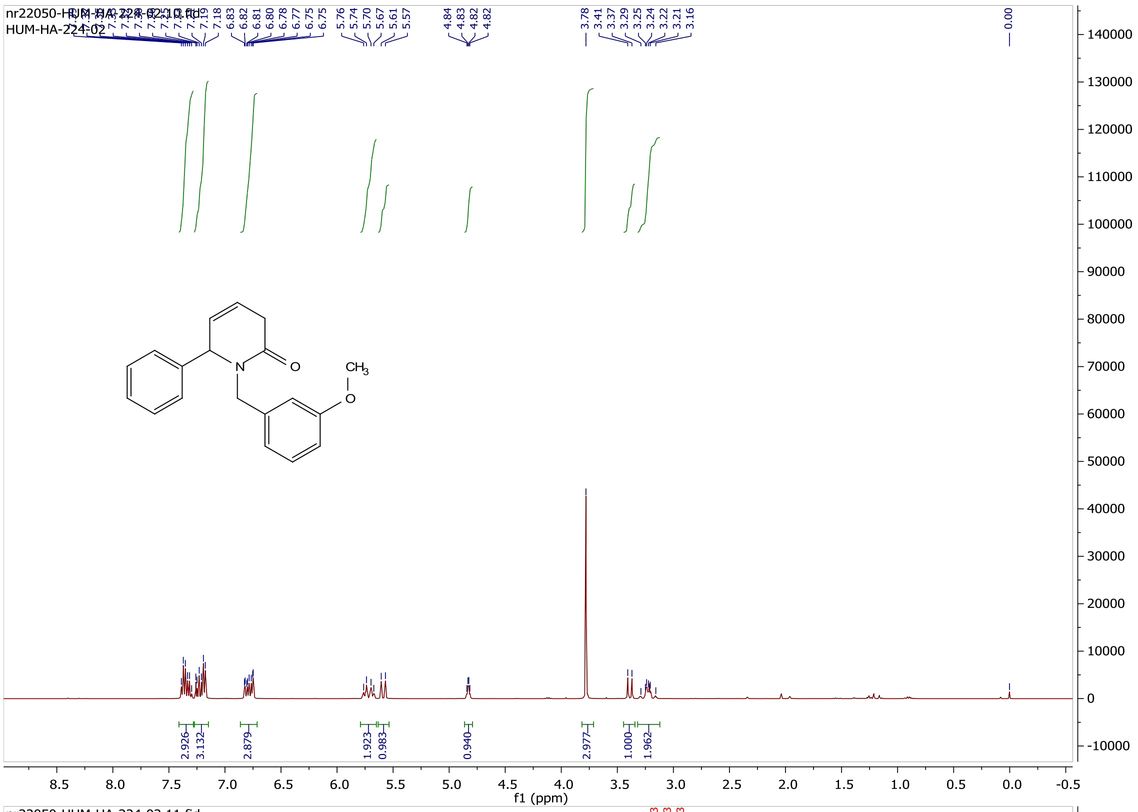
nr22050-HUM-HA-224-02.10.fid
HUM-HA-224-02
6.83 6.82 6.81 6.80 6.78 6.77 6.75 6.75
5.76 5.74 5.70 5.67 5.61 5.57
4.84 4.83 4.82 4.82
3.78 3.41 3.37 3.29 3.25 3.24 3.22 3.21 3.16
0.00
2.926 3.132 2.879 1.923 0.983 0.940 2.977 1.000 1.962
CH3
O
N
f1 (ppm)

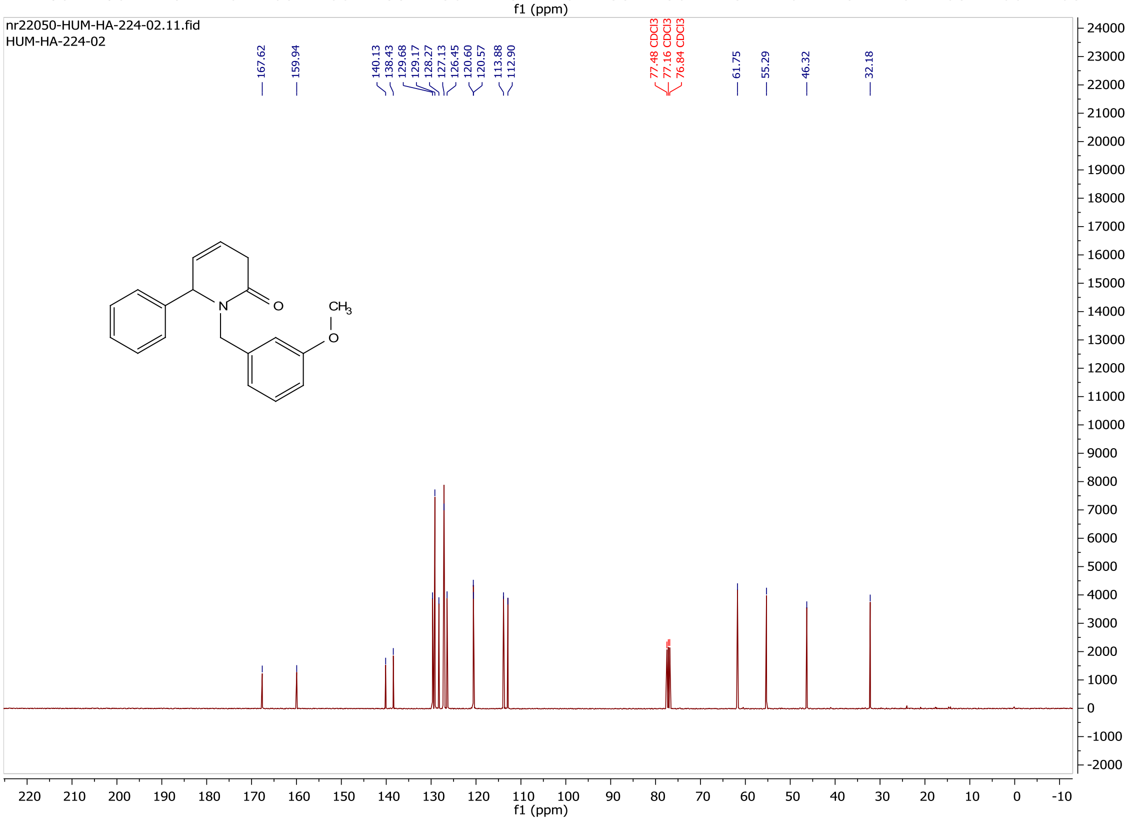
nr22050-HUM-HA-224-02.11.fid
HUM-HA-224-02
167.62
159.94
140.13 138.43 129.68 129.17 128.27 127.13 126.45 120.60 120.57 113.88 112.90
77.48 CDCl3
77.16 CDCl3
76.84 CDCl3
61.75
55.29
46.32
32.18
CH3
O
N
f1 (ppm)

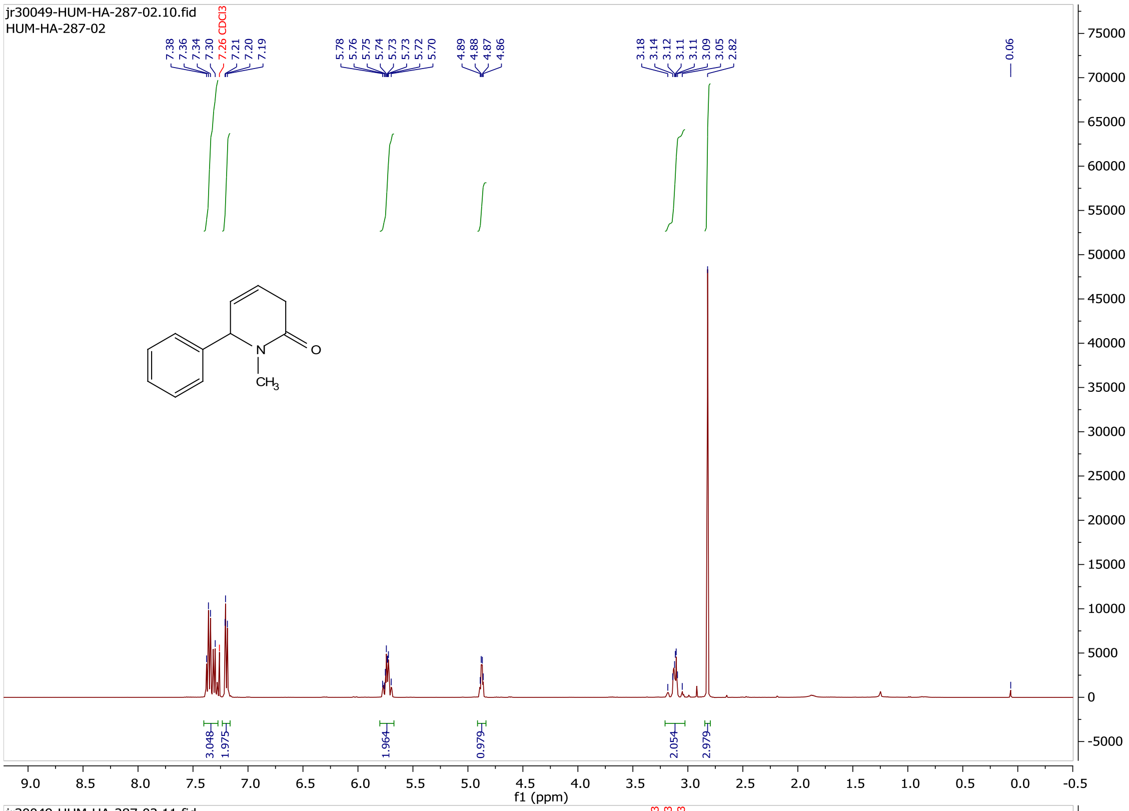
jr30049-HUM-HA-287-02.10.fid
HUM-HA-287-02
7.38
7.36
7.34
7.30
7.26 CDCl3
7.21
7.20
7.19
5.78
5.76
5.75
5.74
5.73
5.73
5.72
5.70
4.89
4.88
4.87
4.86
3.18
3.14
3.12
3.11
3.11
3.09
3.05
2.82
0.06
N
O
CH3
3.048
1.975
1.964
0.979
2.054
2.979
f1 (ppm)

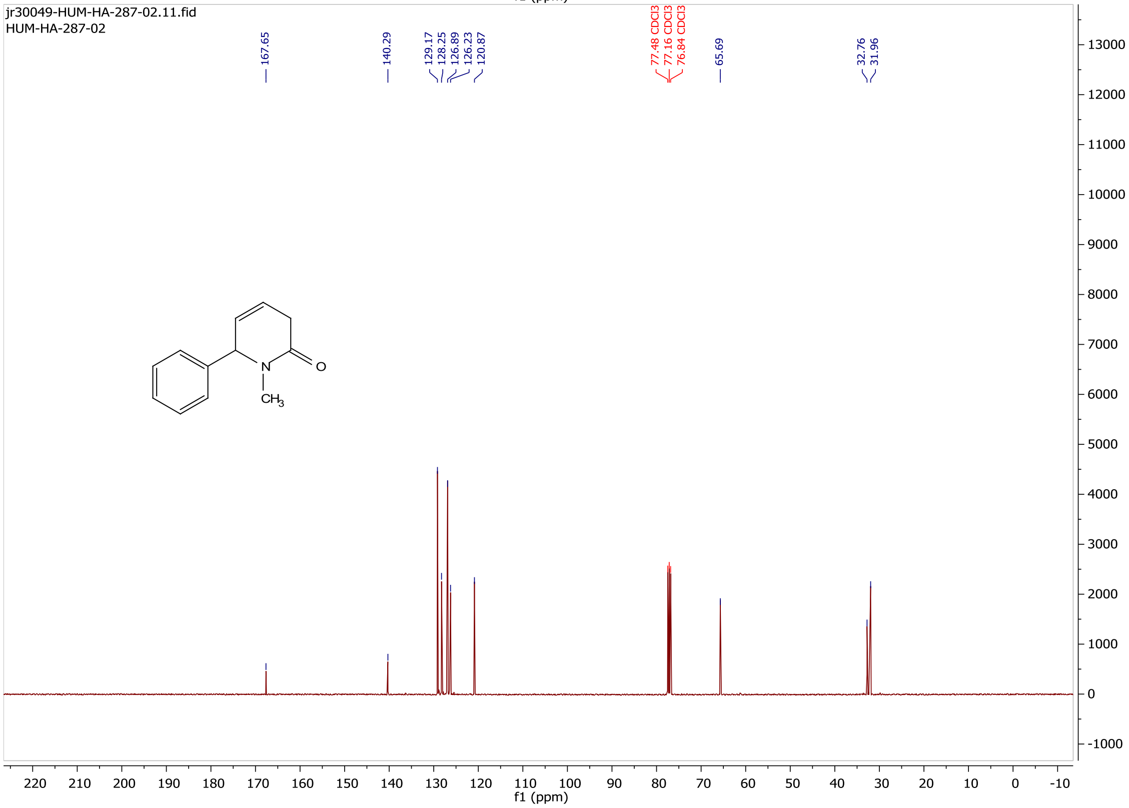
jr30049-HUM-HA-287-02.11.fid
HUM-HA-287-02
167.65
140.29
129.17
128.25
126.89
126.23
120.87
77.48 CDCl3
77.16 CDCl3
76.84 CDCl3
65.69
32.76
31.96
N
O
CH3
f1 (ppm)

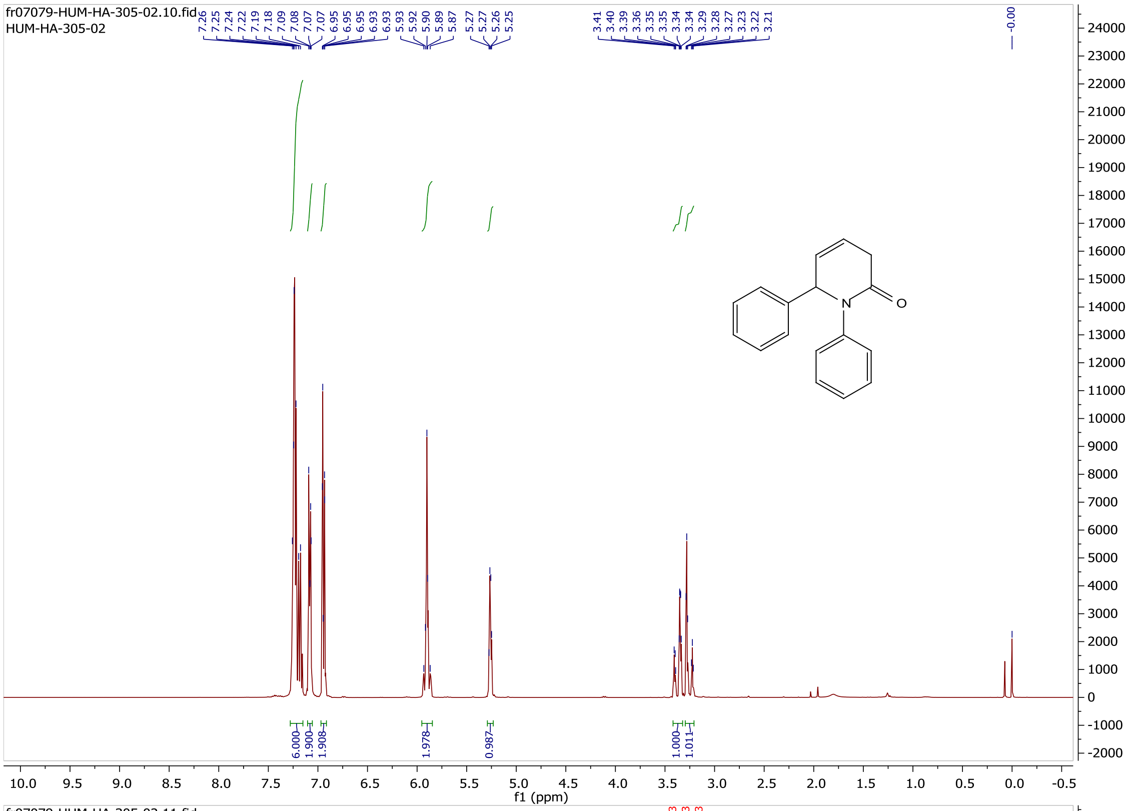
fr07079-HUM-HA-305-02.10.fid
HUM-HA-305-02
7.26 7.25 7.24 7.22 7.19 7.18 7.09 7.08 7.07 7.07 6.95 6.95 6.93 6.93 5.93 5.92 5.90 5.89 5.87 5.27 5.27 5.26 5.25 3.41 3.40 3.39 3.36 3.35 3.35 3.34 3.34 3.29 3.28 3.27 3.23 3.22 3.21 -0.00
N
O
6.000 1.900 1.908 1.978 0.987 1.000 1.011
f1 (ppm)

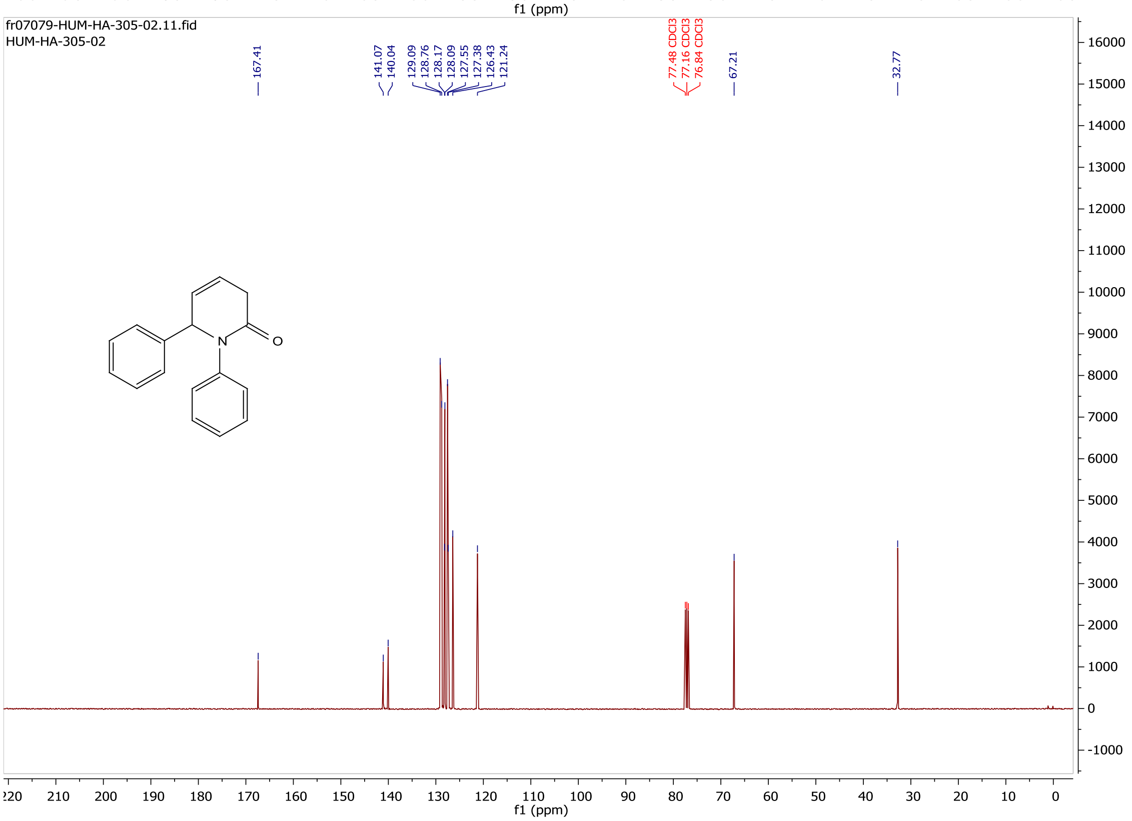
fr07079-HUM-HA-305-02.11.fid
HUM-HA-305-02
167.41 141.07 140.04 129.09 128.76 128.17 128.09 127.55 127.38 126.43 121.24 77.48 CDCl3 77.16 CDCl3 76.84 CDCl3 67.21 32.77
N
O
f1 (ppm)

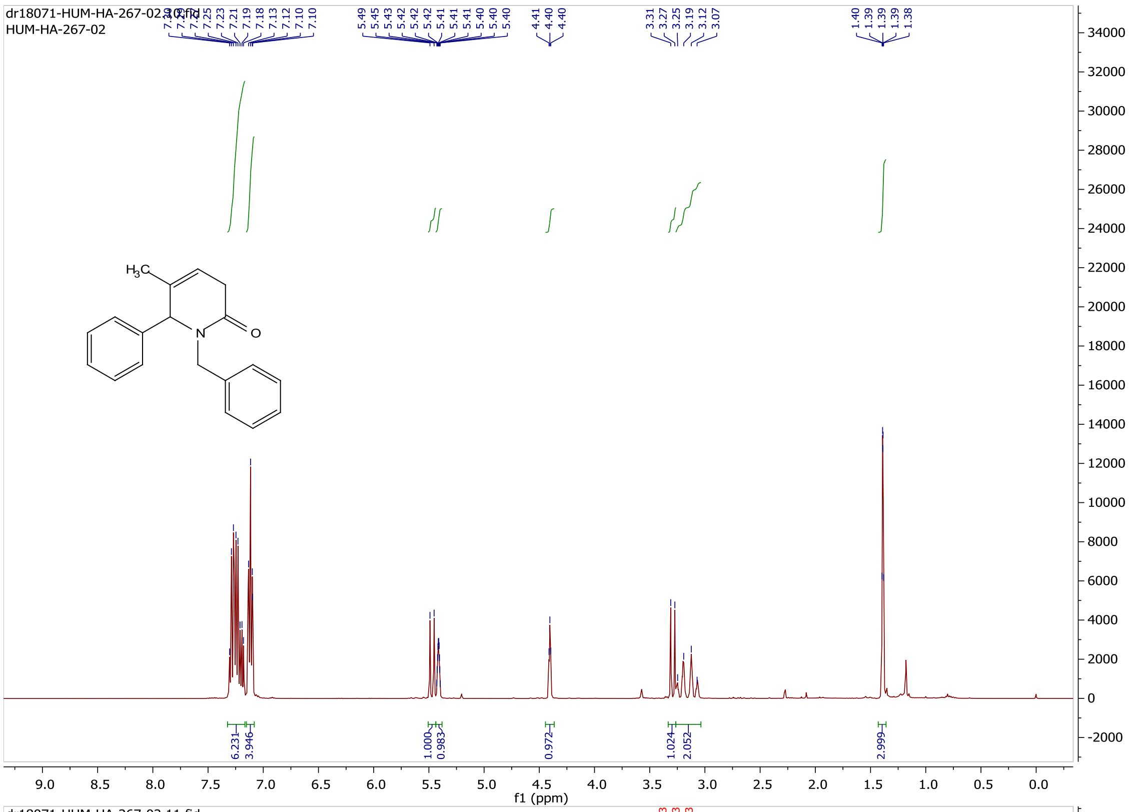
dr18071-HUM-HA-267-02.10.fid
HUM-HA-267-02
f1 (ppm)

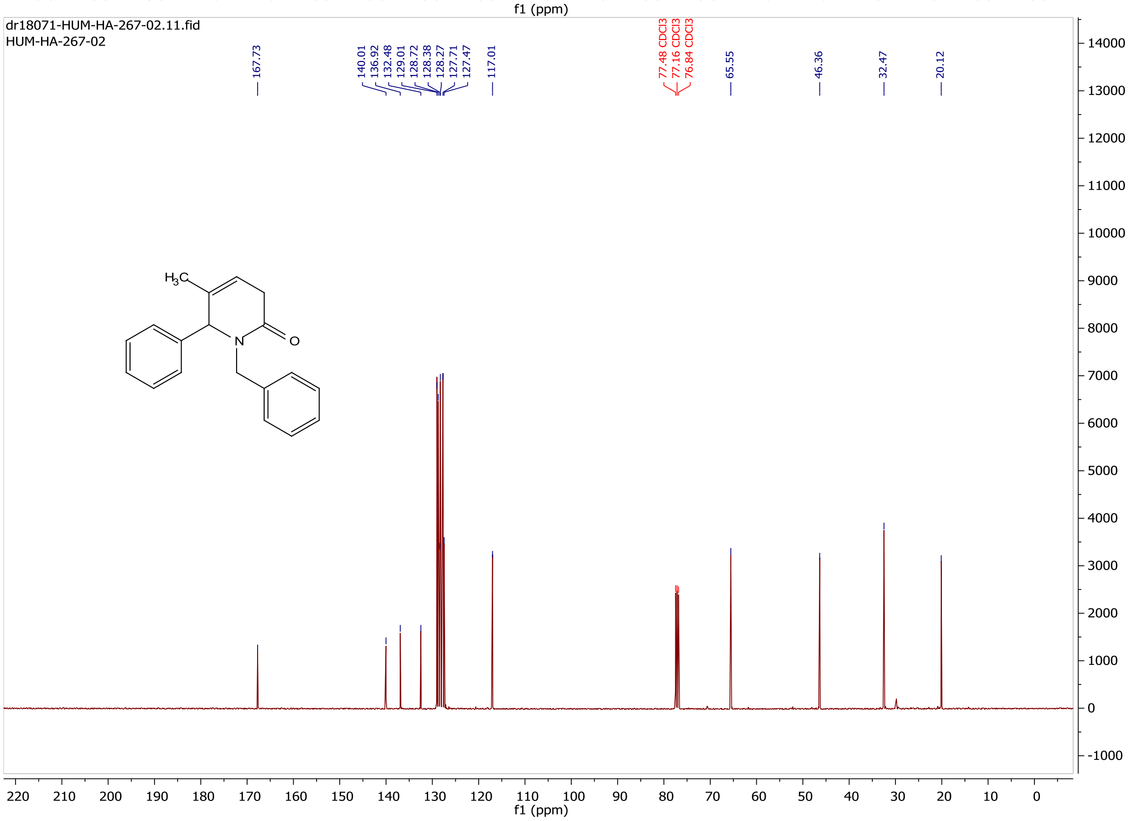
dr18071-HUM-HA-267-02.11.fid
HUM-HA-267-02
167.73
140.01
136.92
132.48
129.01
128.72
128.38
128.27
127.71
127.47
117.01
77.48 CDCl3
77.16 CDCl3
76.84 CDCl3
65.55
46.36
32.47
20.12
f1 (ppm)

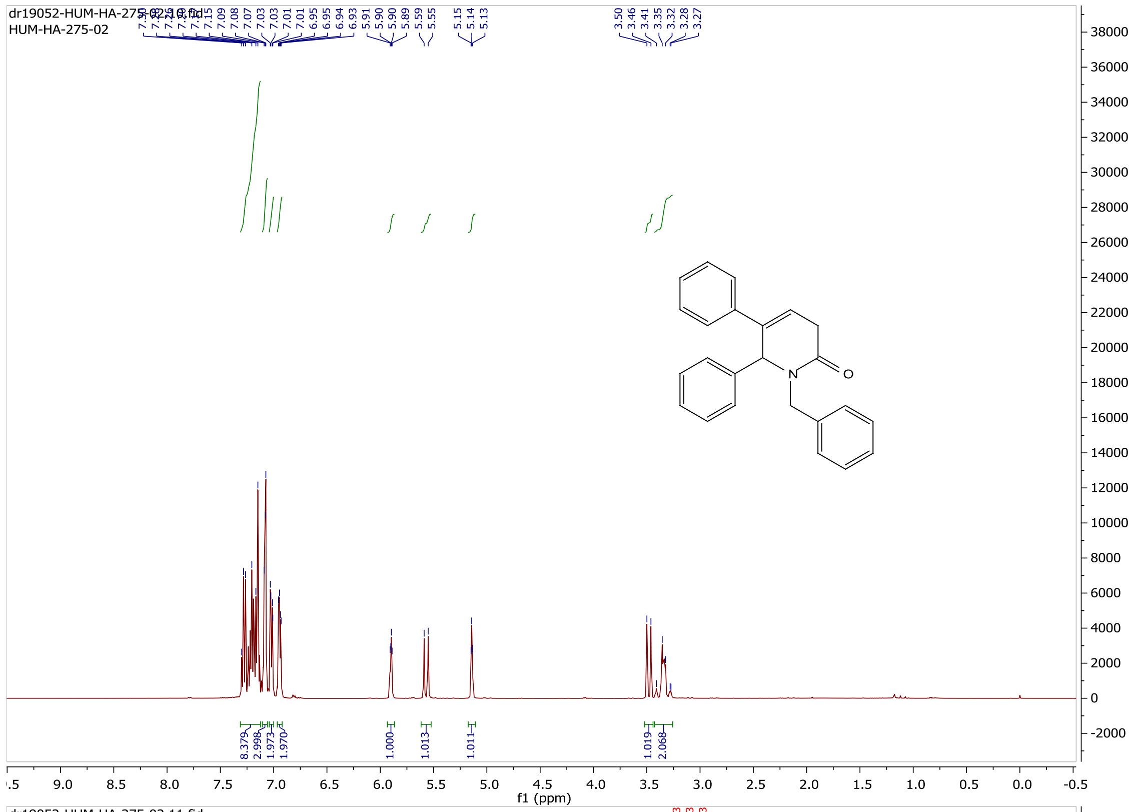
dr19052-HUM-HA-275-02.10.fid
HUM-HA-275-02
f1 (ppm)

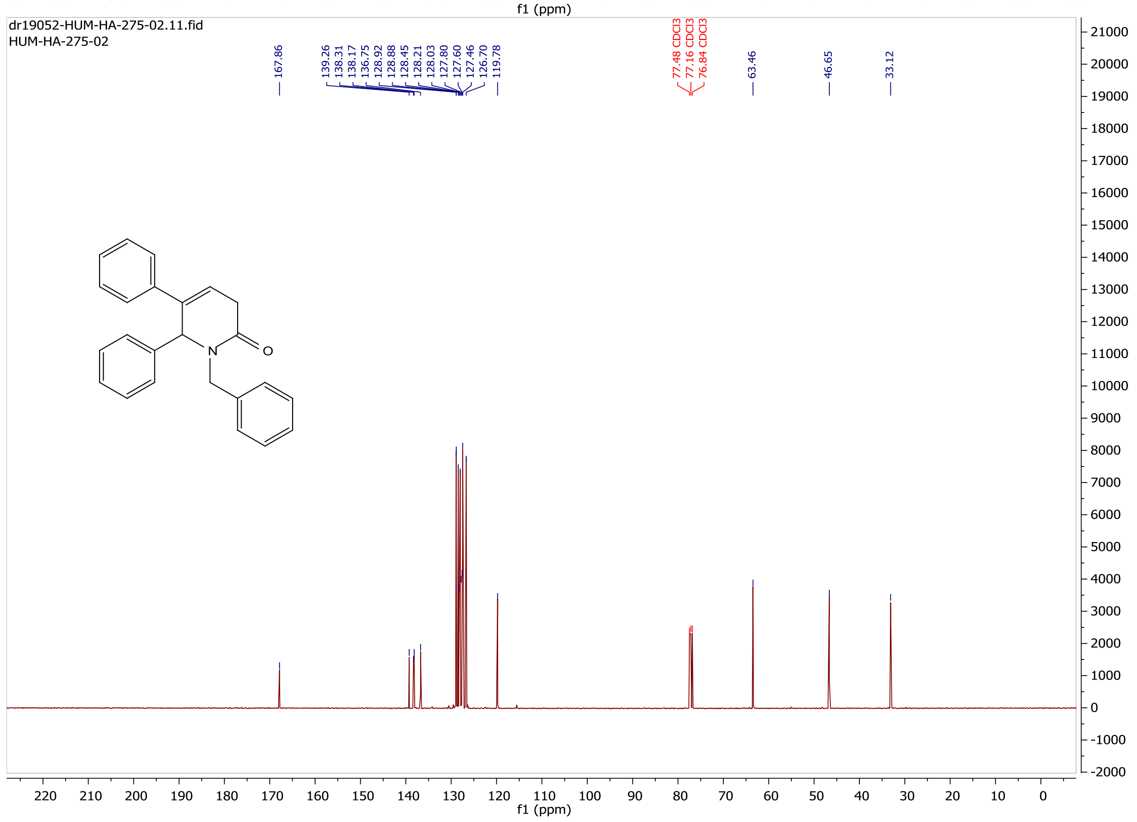
dr19052-HUM-HA-275-02.11.fid
HUM-HA-275-02
167.86
63.46
46.65
33.12
77.48 CDCl3
77.16 CDCl3
76.84 CDCl3
f1 (ppm)

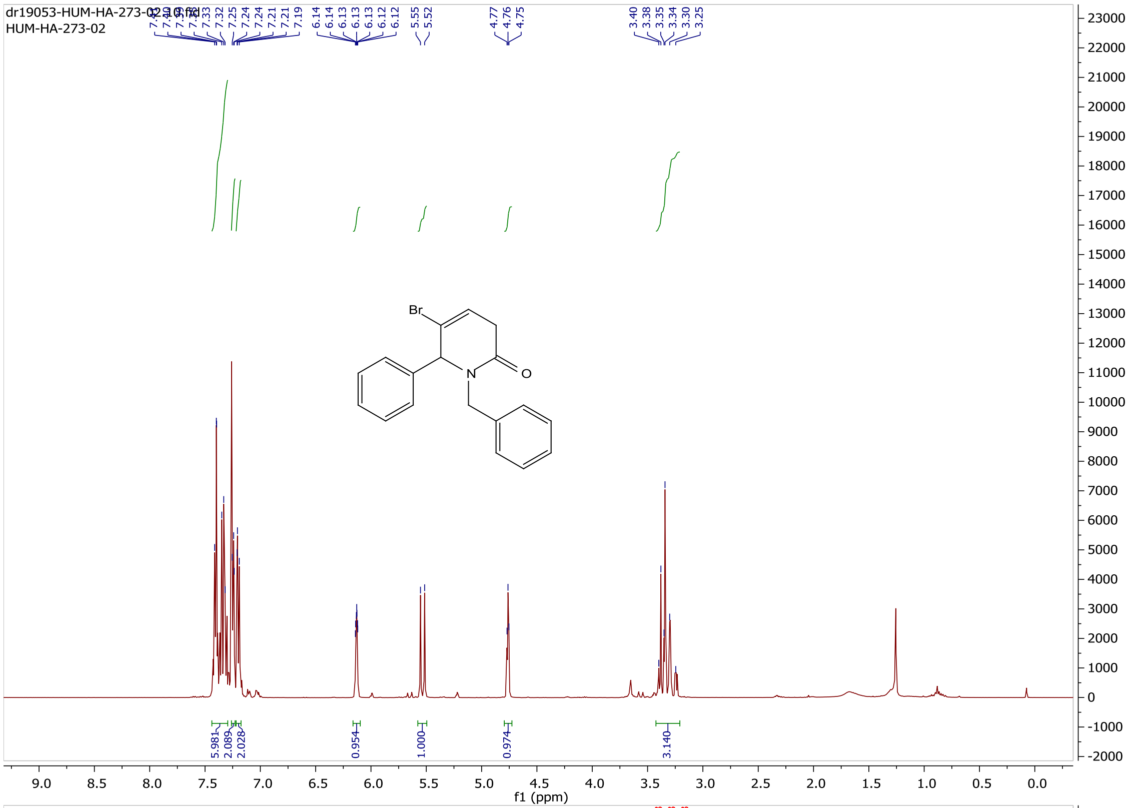
dr19053-HUM-HA-273-02.10.fid
HUM-HA-273-02
7.32
7.25
7.24
7.24
7.21
7.21
7.19
6.14
6.14
6.13
6.13
6.13
6.12
6.12
5.55
5.52
4.77
4.76
4.75
3.40
3.38
3.35
3.34
3.30
3.25
Br
N
O
5.981
2.089
2.028
0.954
1.000
0.974
3.140
f1 (ppm)

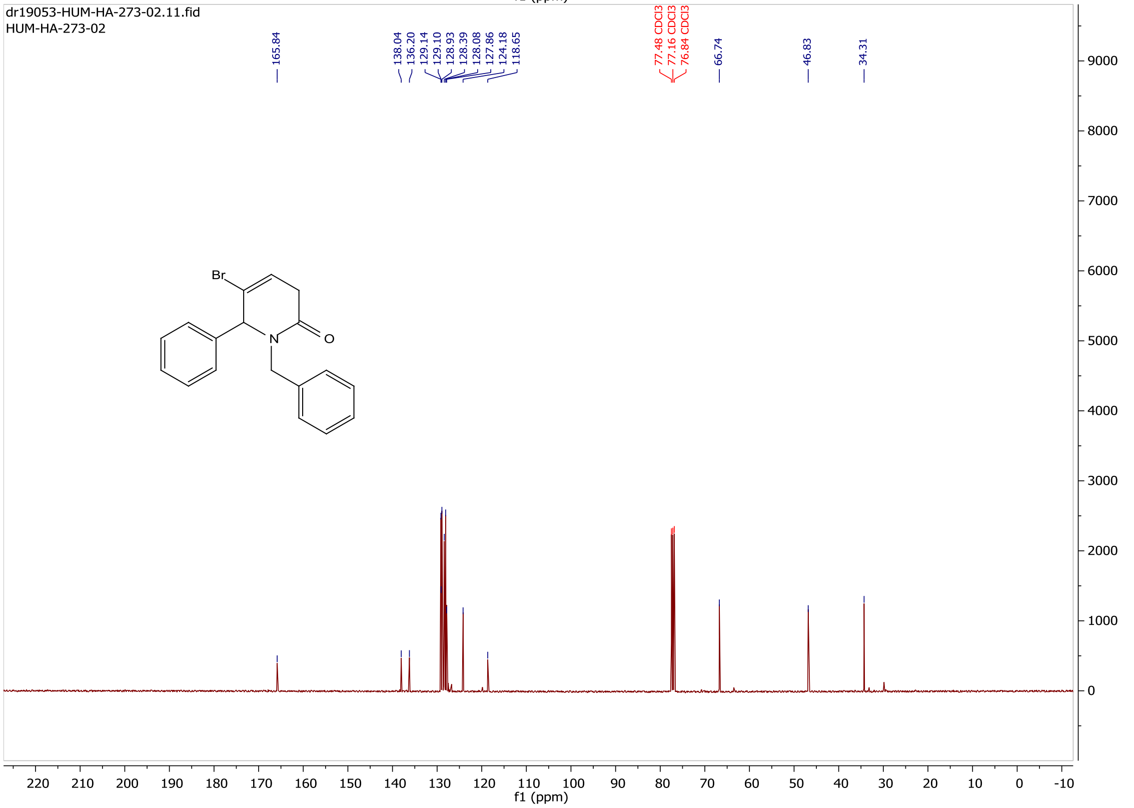
dr19053-HUM-HA-273-02.11.fid
HUM-HA-273-02
165.84
138.04
136.20
129.14
129.10
128.93
128.39
128.08
127.86
124.18
118.65
77.48 CDCl3
77.16 CDCl3
76.84 CDCl3
66.74
46.83
34.31
Br
N
O
f1 (ppm)

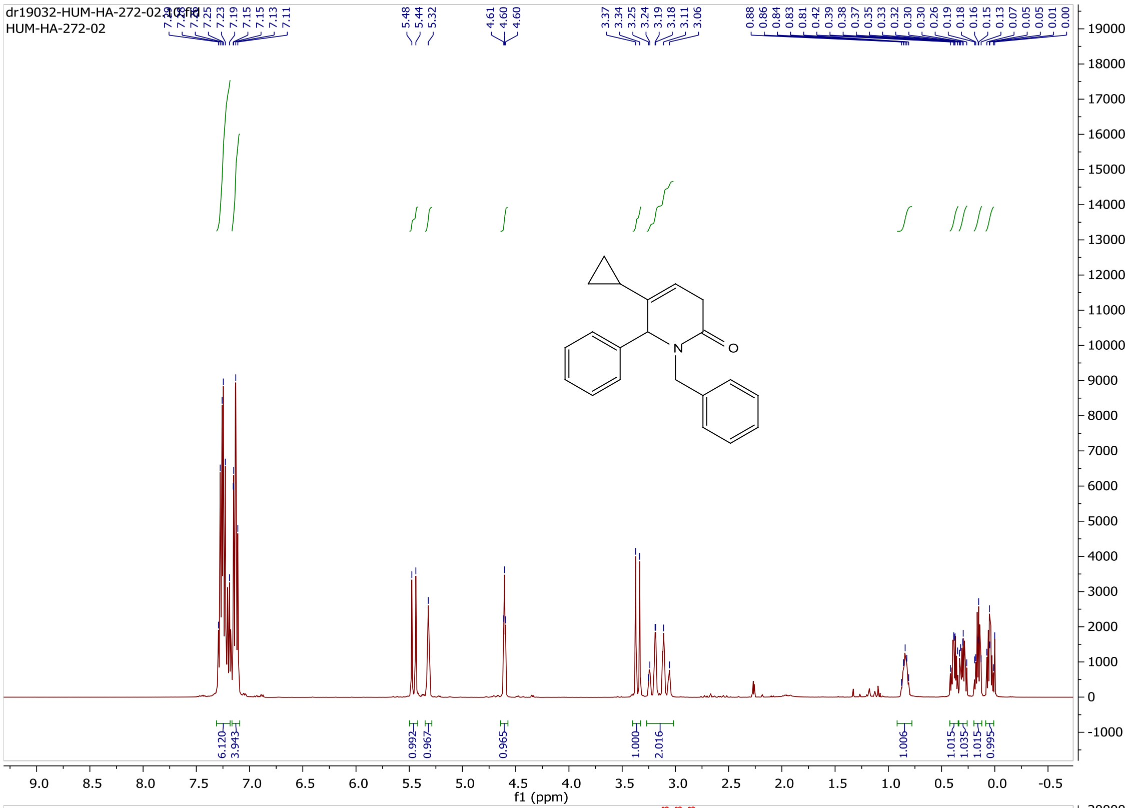
dr19032-HUM-HA-272-02.10.fid
HUM-HA-272-02
f1 (ppm)

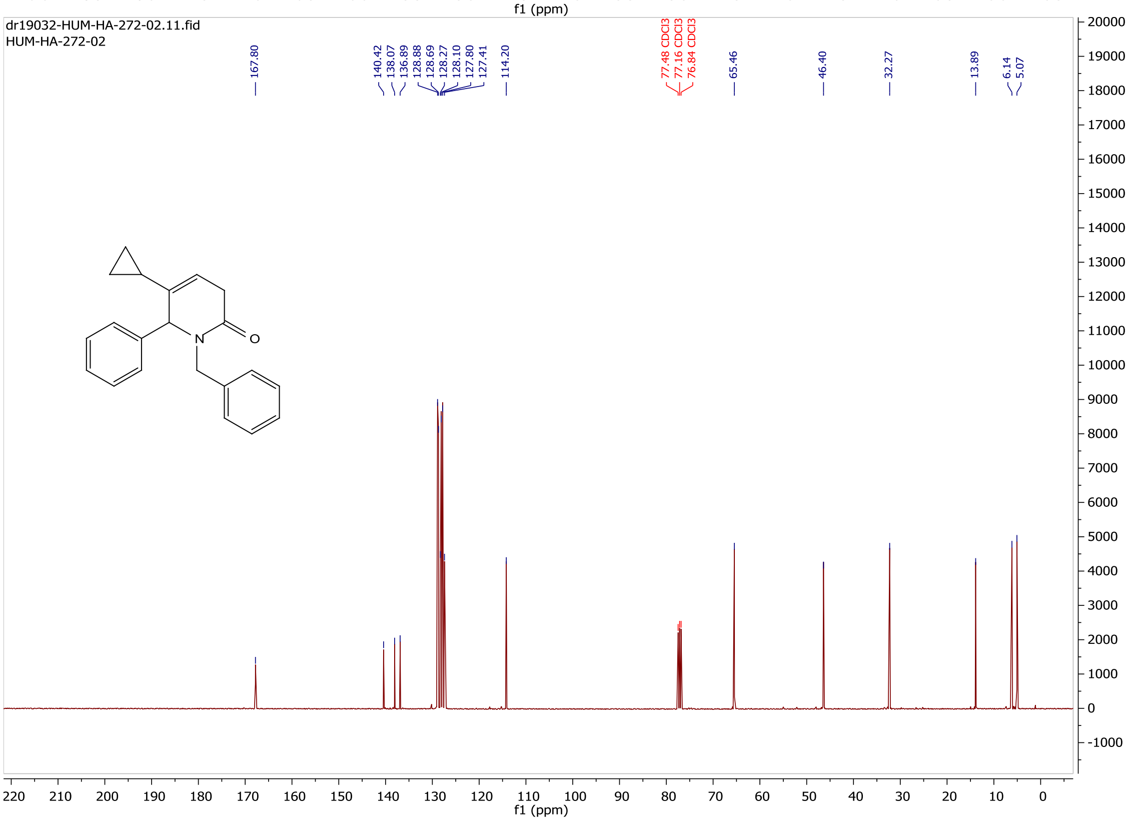
dr19032-HUM-HA-272-02.11.fid
HUM-HA-272-02
167.80
140.42
138.07
136.89
128.88
128.69
128.27
128.10
127.80
127.41
114.20
77.48 CDCl3
77.16 CDCl3
76.84 CDCl3
65.46
46.40
32.27
13.89
6.14
5.07
f1 (ppm)

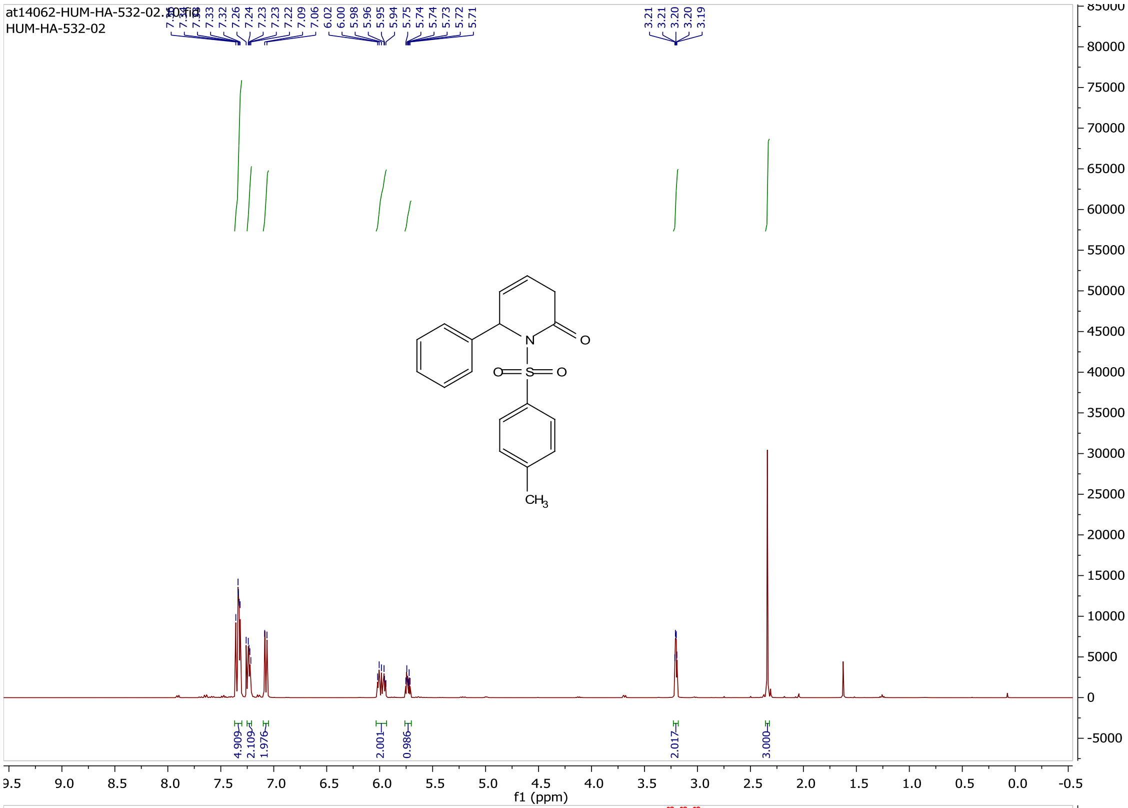
at14062-HUM-HA-532-02.10.fid
HUM-HA-532-02
7.33
7.32
7.26
7.24
7.23
7.23
7.22
7.09
7.06
6.02
6.00
5.98
5.96
5.95
5.94
5.75
5.74
5.74
5.73
5.72
5.71
3.21
3.21
3.20
3.20
3.19
4.909
2.109
1.976
2.001
0.986
2.017
3.000
N
O
S
O
O
CH3
f1 (ppm)

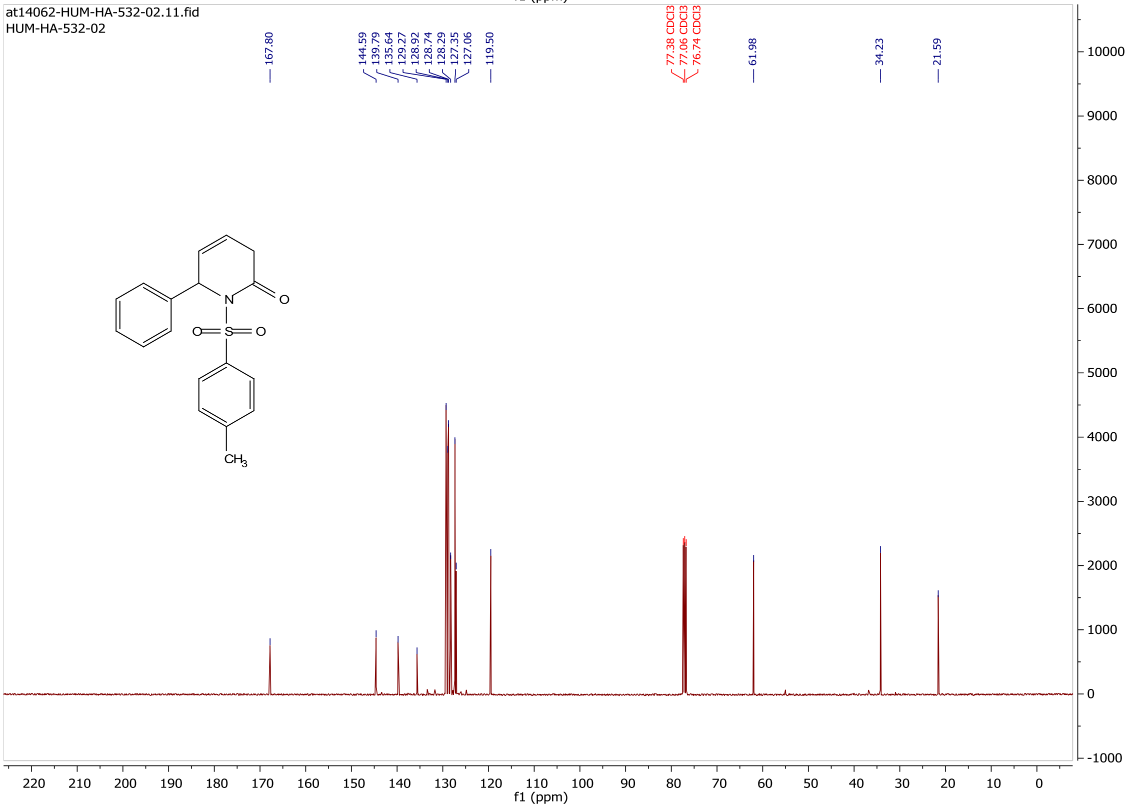
at14062-HUM-HA-532-02.11.fid
HUM-HA-532-02
167.80
144.59
139.79
135.64
129.27
128.92
128.74
128.29
127.35
127.06
119.50
77.38 CDCl3
77.06 CDCl3
76.74 CDCl3
61.98
34.23
21.59
N
O
S
O
O
CH3
f1 (ppm)

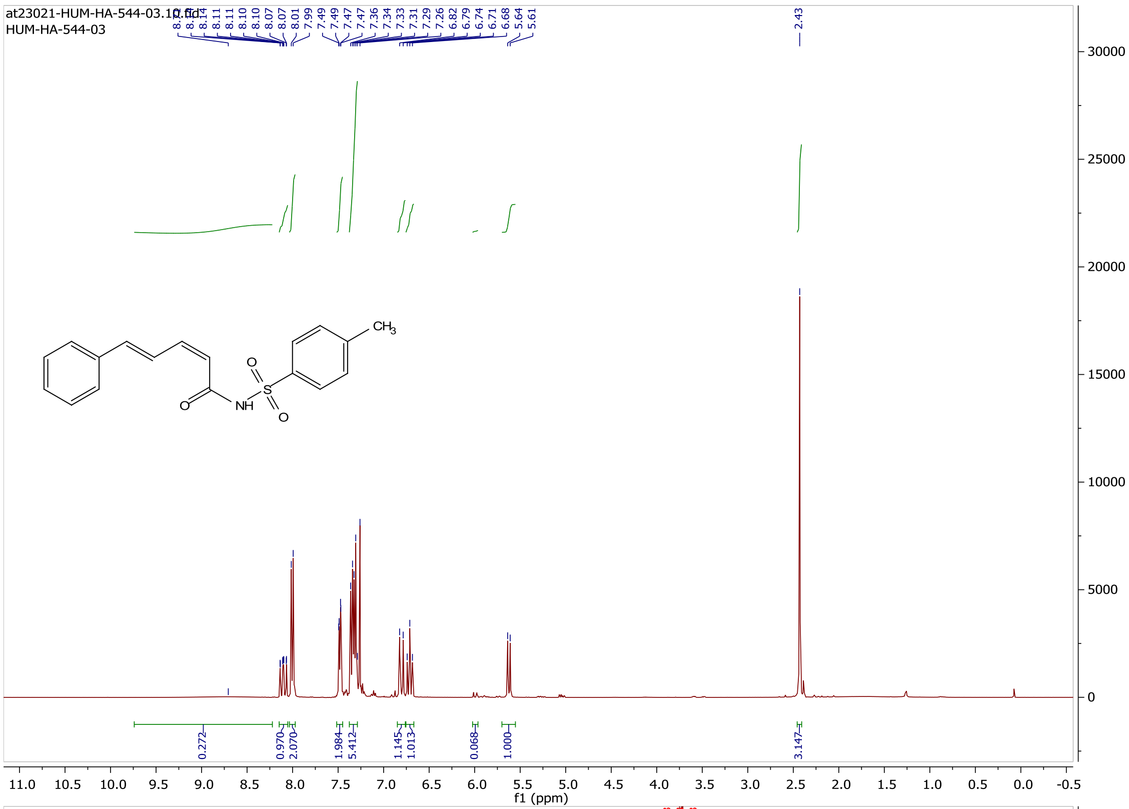
at23021-HUM-HA-544-03.10.fid
HUM-HA-544-03
2.43
CH3
NH
f1 (ppm)

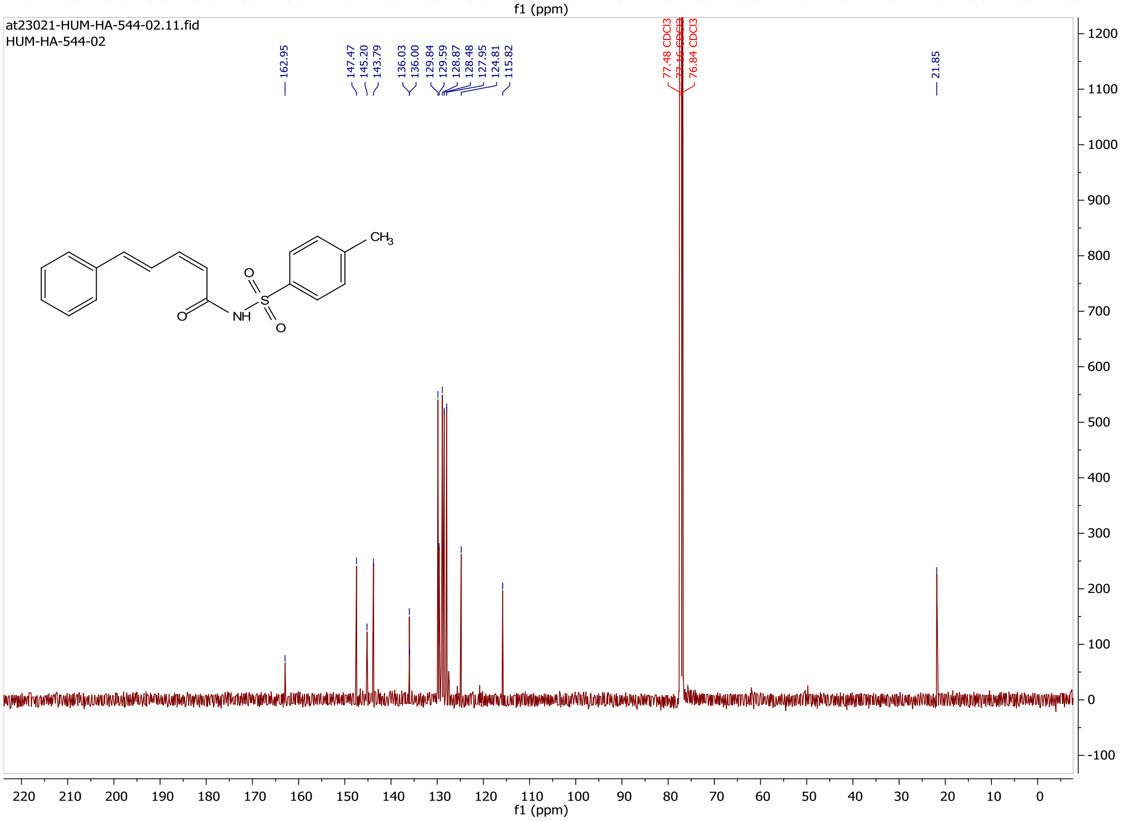
at23021-HUM-HA-544-02.11.fid
HUM-HA-544-02
162.95
147.47
145.20
143.79
136.03
136.00
129.84
129.59
128.87
128.48
127.95
124.81
115.82
77.48 CDCl3
76.84 CDCl3
21.85
CH3
NH
f1 (ppm)

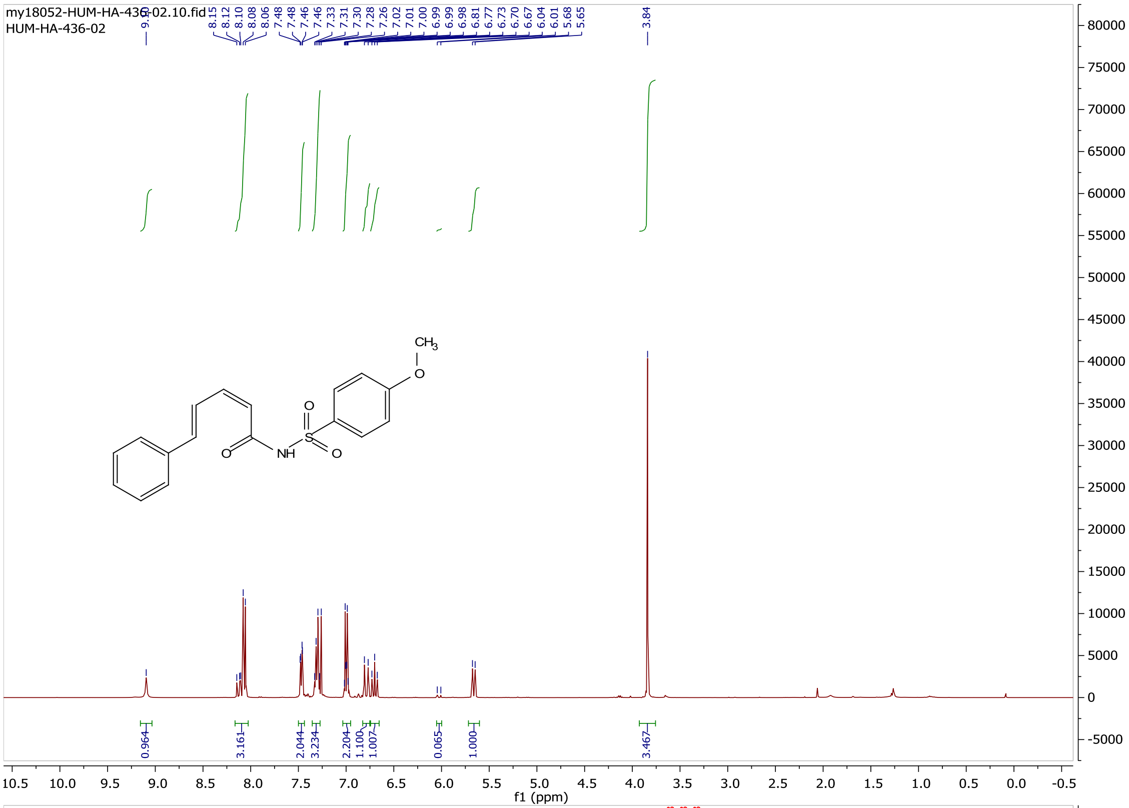
my18052-HUM-HA-436-02.10.fid
HUM-HA-436-02
9.11
8.15
8.12
8.10
8.08
8.06
7.48
7.46
7.46
7.33
7.31
7.30
7.28
7.26
7.02
7.01
7.00
6.99
6.99
6.98
6.81
6.77
6.73
6.70
6.67
6.04
6.01
5.68
5.65
3.84
CH3
O
O
S
O
O
NH
0.964
3.161
2.044
3.234
2.204
1.100
1.007
0.065
1.000
3.467
f1 (ppm)

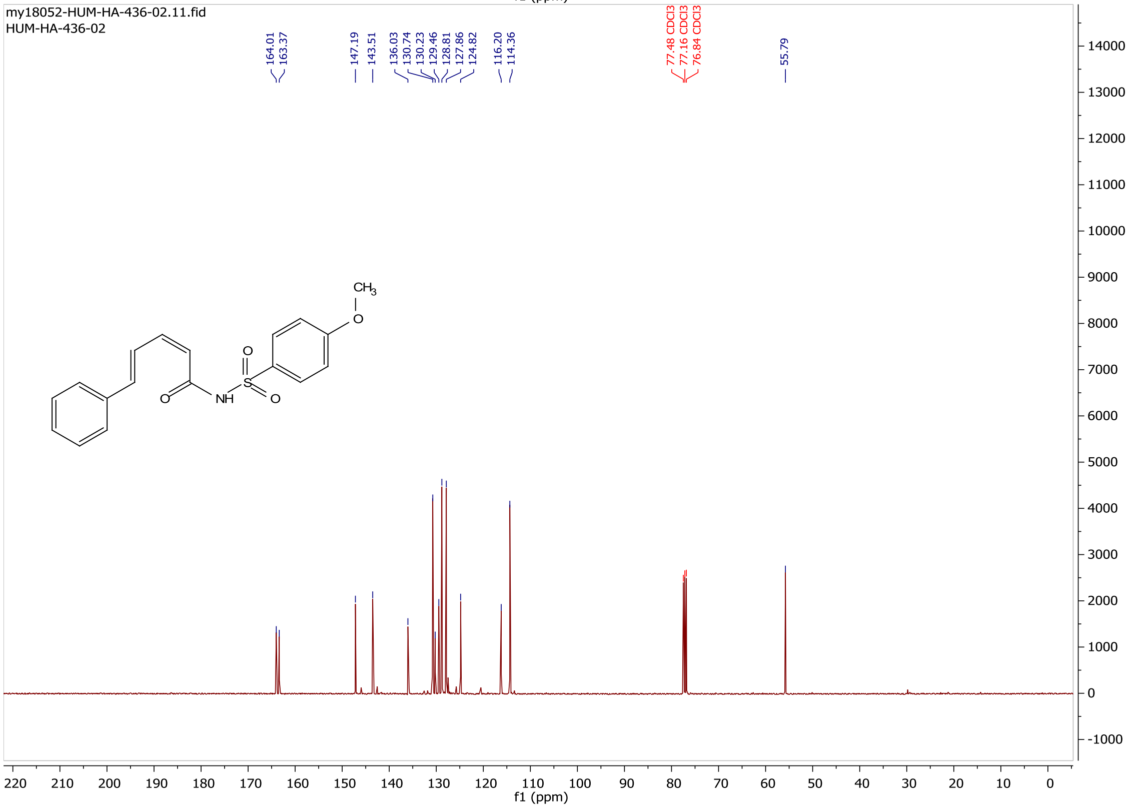
my18052-HUM-HA-436-02.11.fid
HUM-HA-436-02
164.01
163.37
147.19
143.51
136.03
130.74
130.23
129.46
128.81
127.86
124.82
116.20
114.36
77.48 CDCl3
77.16 CDCl3
76.84 CDCl3
55.79
CH3
O
O
S
O
O
NH
f1 (ppm)

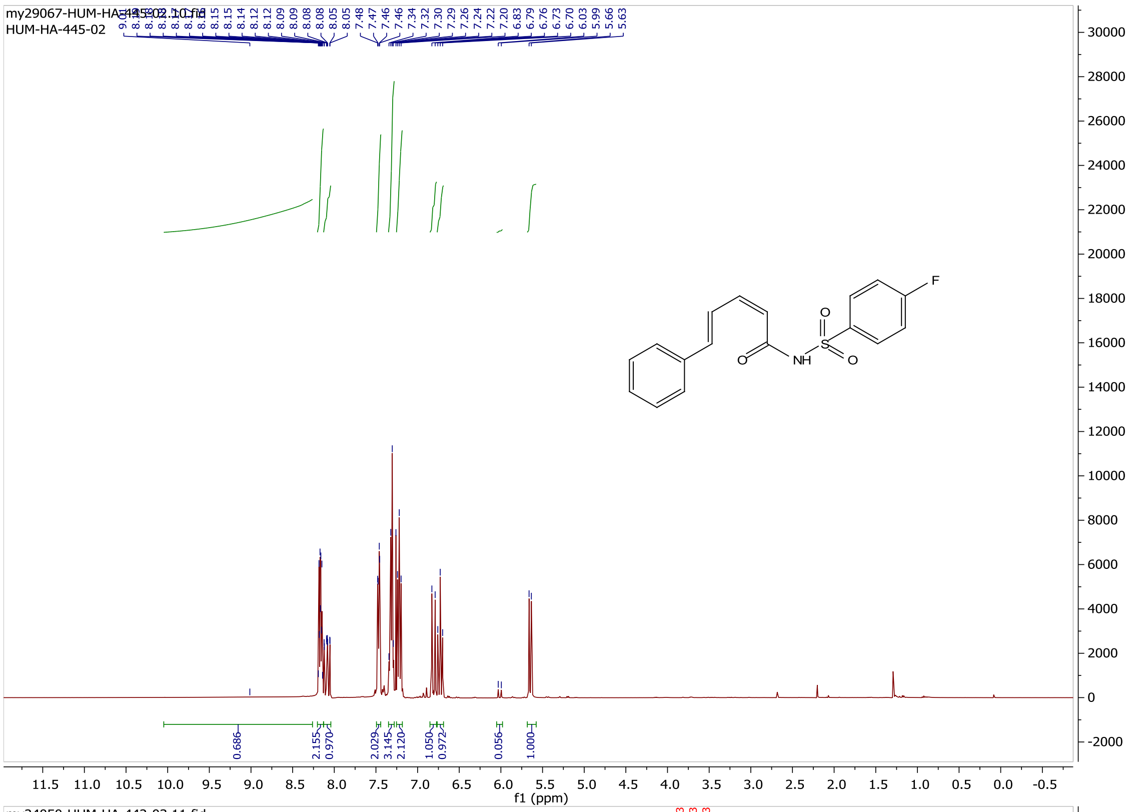
my29067-HUM-HA-445-02.10.fid
HUM-HA-445-02
F
O
S
O
O
NH
f1 (ppm)

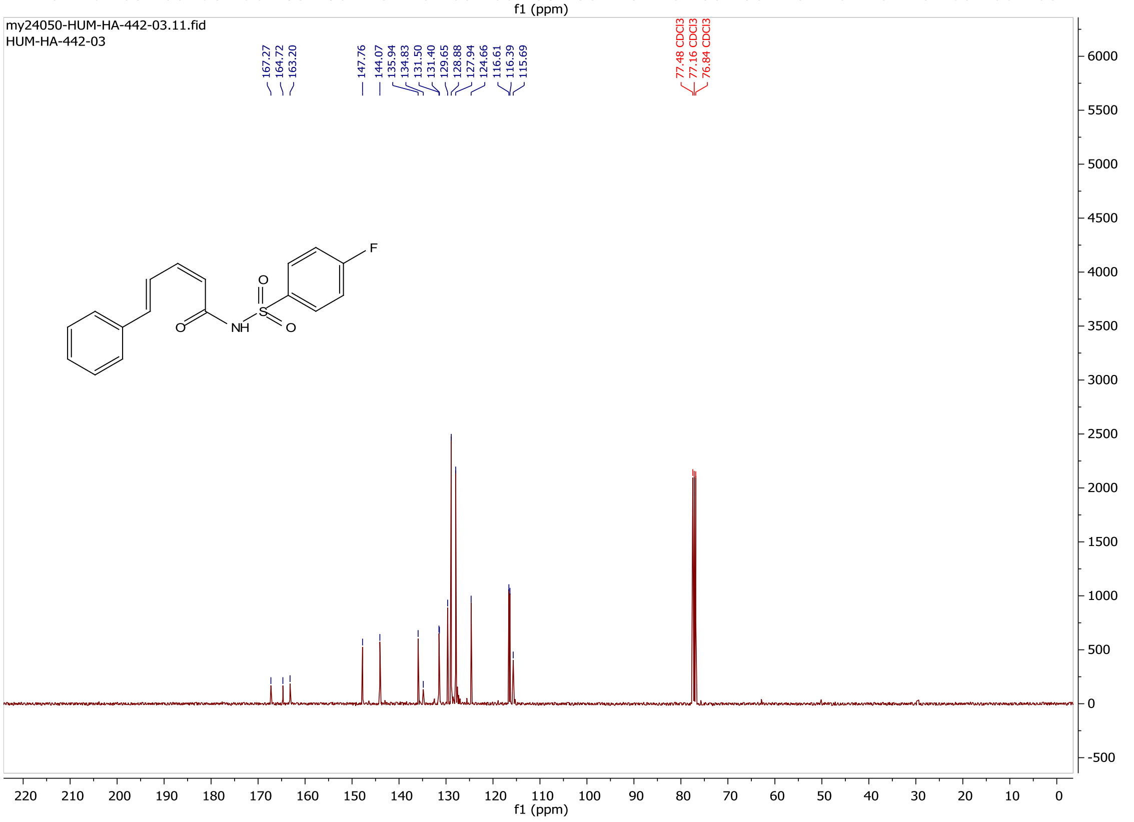
my24050-HUM-HA-442-03.11.fid
HUM-HA-442-03
167.27
164.72
163.20
147.76
144.07
135.94
134.83
131.50
131.40
129.65
128.88
127.94
124.66
116.61
116.39
115.69
77.48 CDCl3
77.16 CDCl3
76.84 CDCl3
F
O
S
O
O
NH
f1 (ppm)

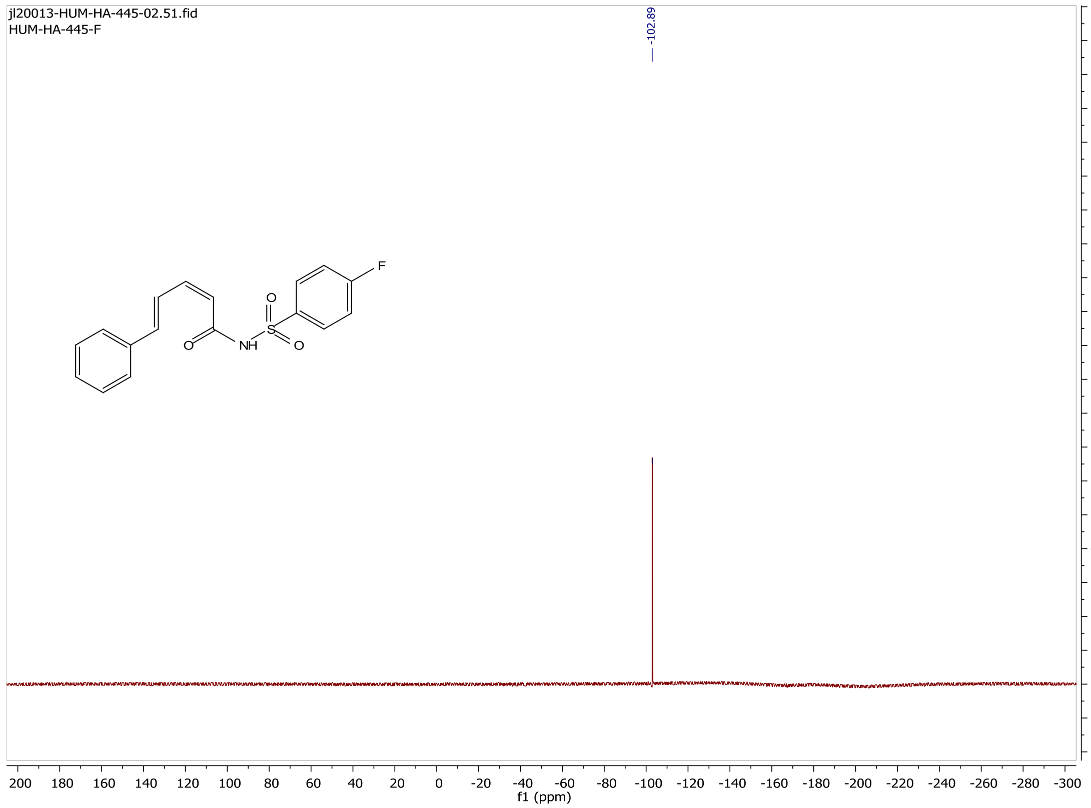
jl20013-HUM-HA-445-02.51.fid
HUM-HA-445-F
-102.89
F
O
S
NH
O
O
20000
19000
18000
17000
16000
15000
14000
13000
12000
11000
10000
9000
8000
7000
6000
5000
4000
3000
2000
1000
0
-1000
-2000
200 180 160 140 120 100 80 60 40 20 0 -20 -40 -60 -80 -100 -120 -140 -160 -180 -200 -220 -240 -260 -280 -300
f1 (ppm)

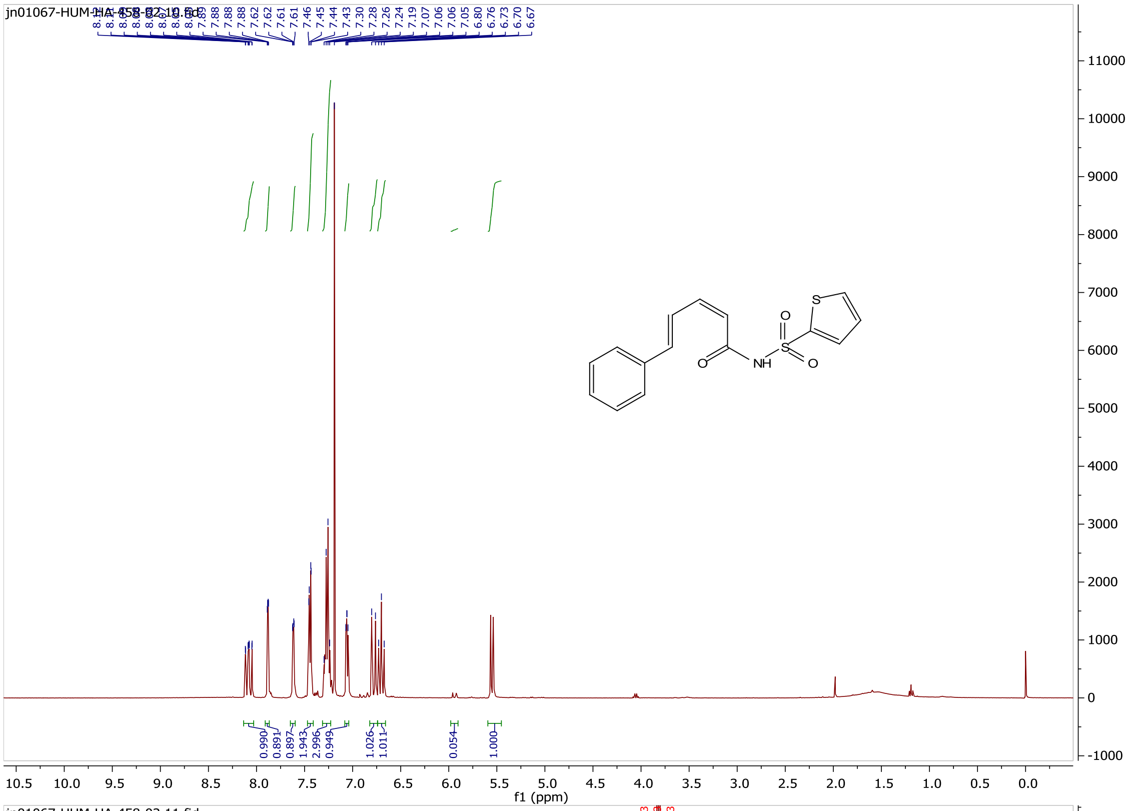
jn01067-HUM-HA-458-02.10.fid
7.88
7.88
7.88
7.62
7.62
7.61
7.61
7.46
7.45
7.44
7.43
7.30
7.28
7.26
7.24
7.19
7.07
7.06
7.06
7.05
6.80
6.76
6.73
6.70
6.67
0.990
0.891
0.897
1.943
2.996
0.949
1.026
1.011
0.054
1.000
O
NH
S
O
O
S
f1 (ppm)

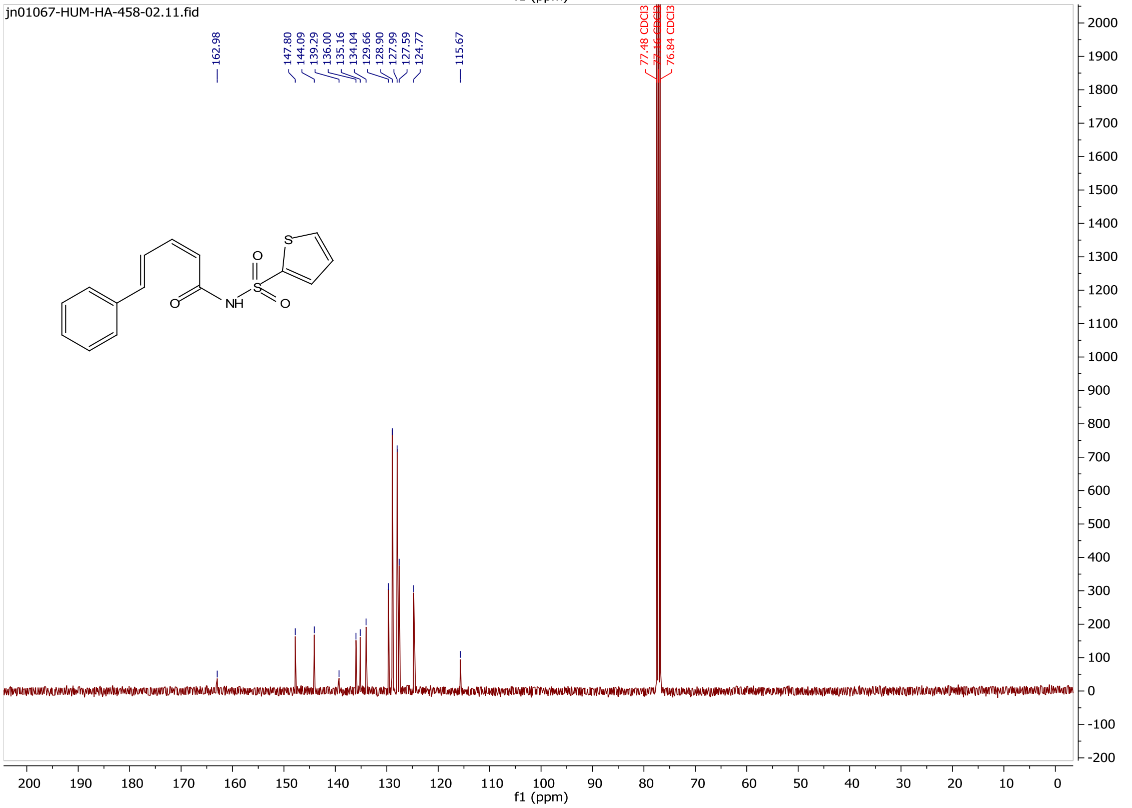
jn01067-HUM-HA-458-02.11.fid
162.98
147.80
144.09
139.29
136.00
135.16
134.04
129.66
128.90
127.99
127.59
124.77
115.67
77.48 CDCl3
76.84 CDCl3
O
NH
S
O
O
S
f1 (ppm)

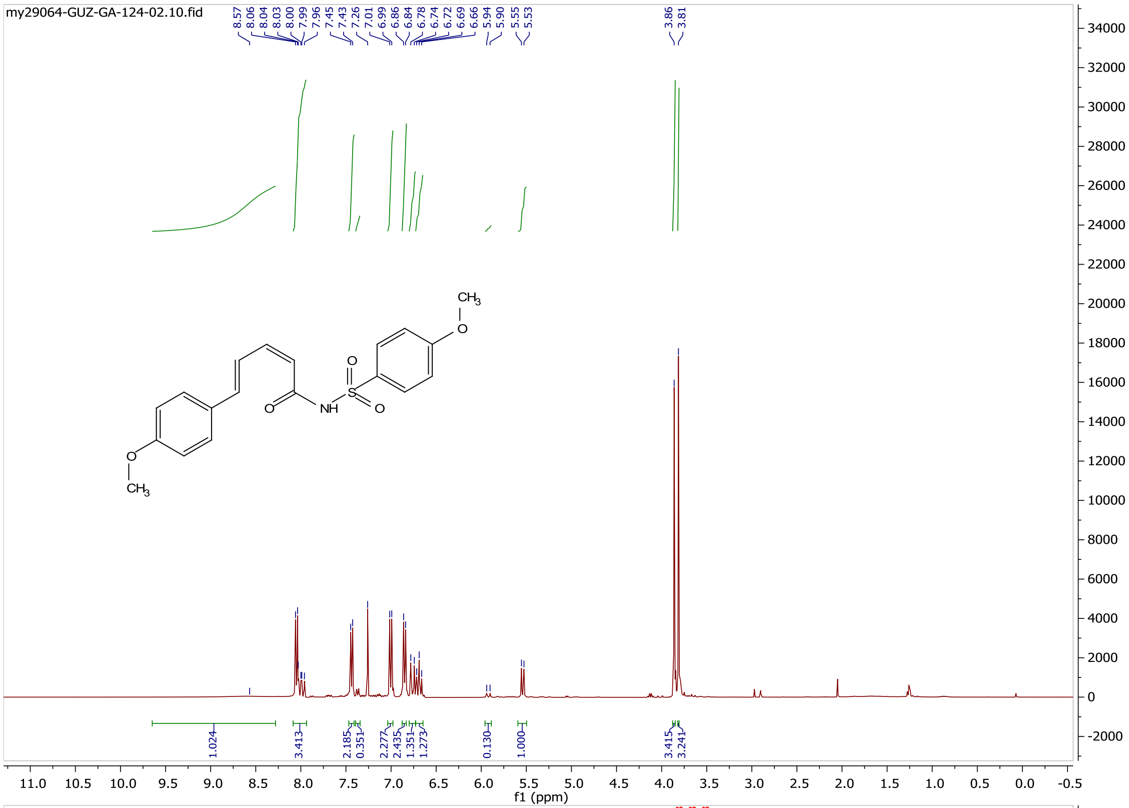
my29064-GUZ-GA-124-02.10.fid
8.57 8.06 8.04 8.03 8.00 7.99 7.96 7.45 7.43 7.26 7.01 6.99 6.86 6.84 6.78 6.74 6.72 6.69 6.66 5.94 5.90 5.55 5.53
3.86 3.81
1.024 3.413 2.185 0.351 2.277 2.435 1.351 1.273 0.130 1.000 3.415 3.241
f1 (ppm)

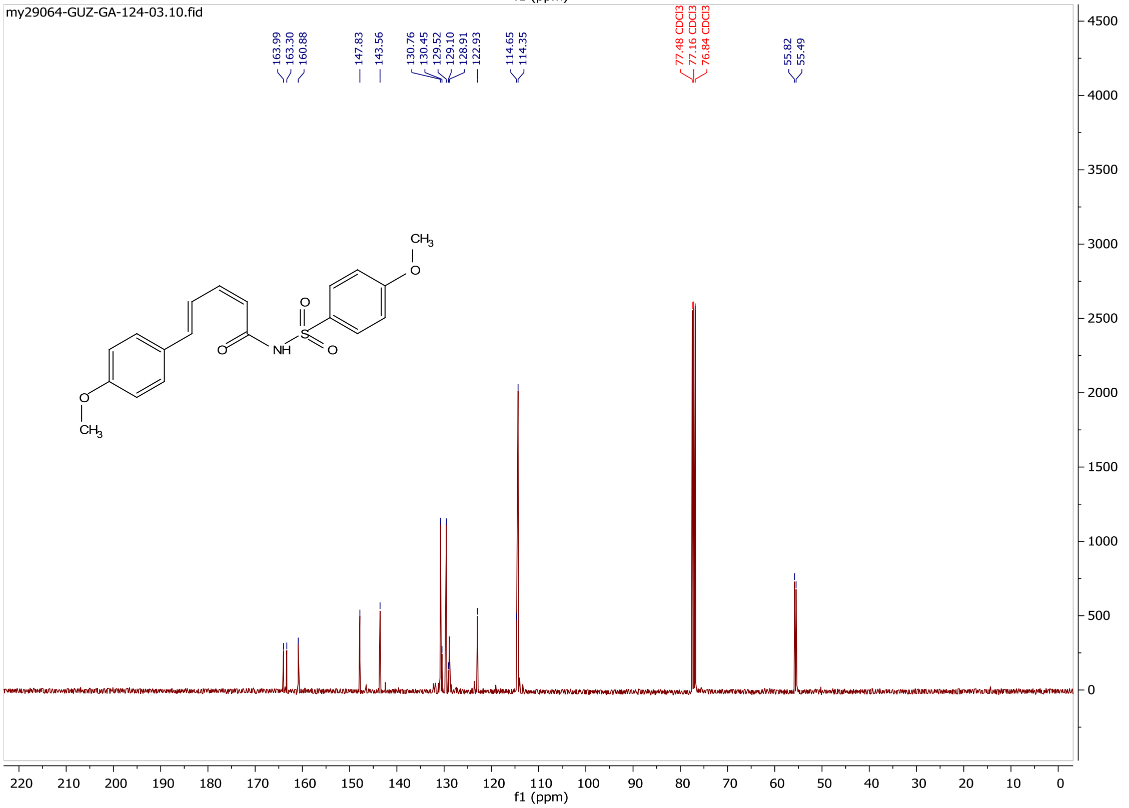
my29064-GUZ-GA-124-03.10.fid
163.99 163.30 160.88 147.83 143.56 130.76 130.45 129.52 129.10 128.91 122.93 114.65 114.35
77.48 CDCl3 77.16 CDCl3 76.84 CDCl3
55.82 55.49
f1 (ppm)

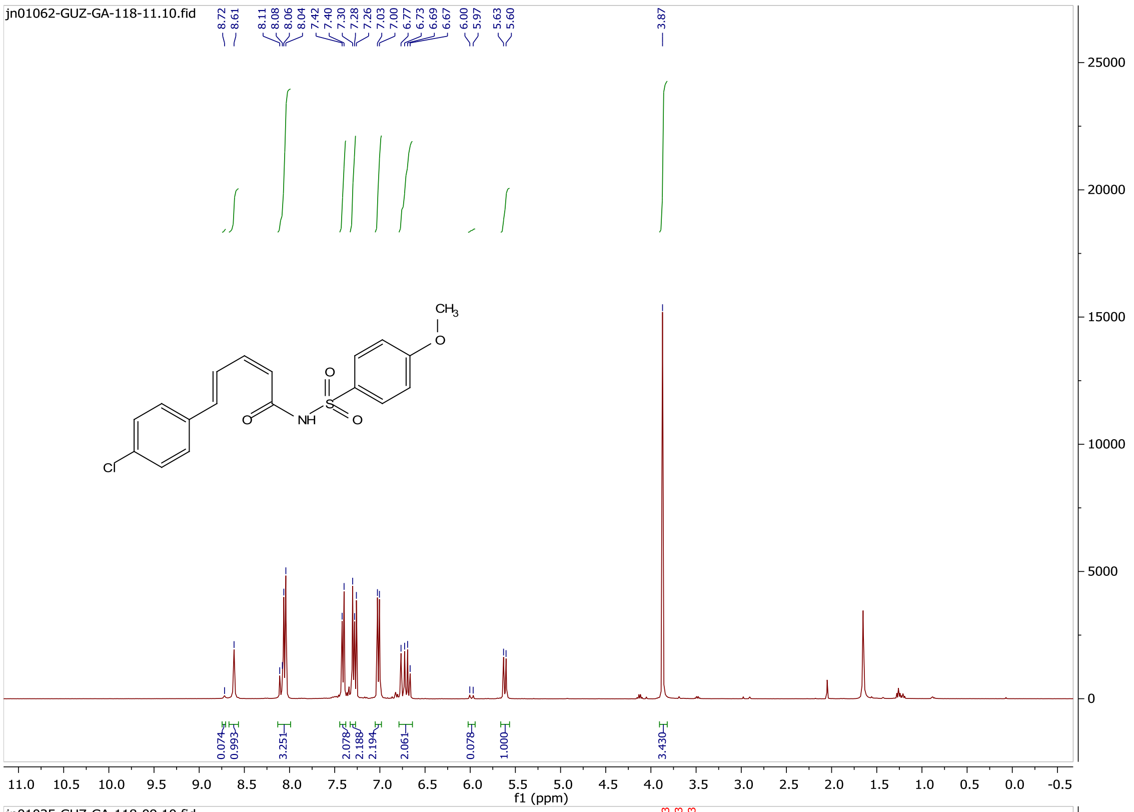

jn01062-GUZ-GA-118-11.10.fid
8.72
8.61
8.11
8.08
8.06
8.04
7.42
7.40
7.30
7.28
7.26
7.03
7.00
6.77
6.73
6.69
6.67
6.00
5.97
5.63
5.60
3.87
CH3
O
O
S
O
NH
O
Cl
0.074
0.993
3.251
2.078
2.188
2.194
2.061
0.078
1.000
3.430
f1 (ppm)

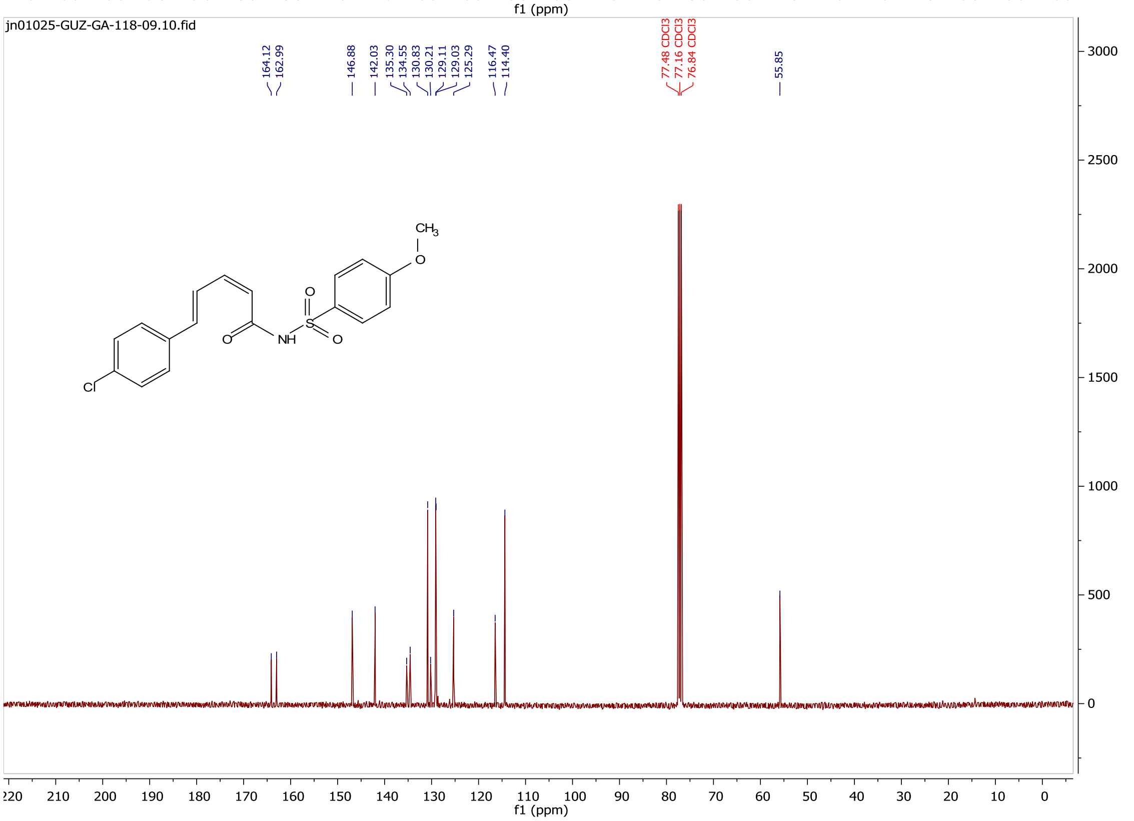

jn01025-GUZ-GA-118-09.10.fid
164.12
162.99
146.88
142.03
135.30
134.55
130.83
130.21
129.11
129.03
125.29
116.47
114.40
77.48 CDCl3
77.16 CDCl3
76.84 CDCl3
55.85
CH3
O
O
S
O
NH
O
Cl
f1 (ppm)

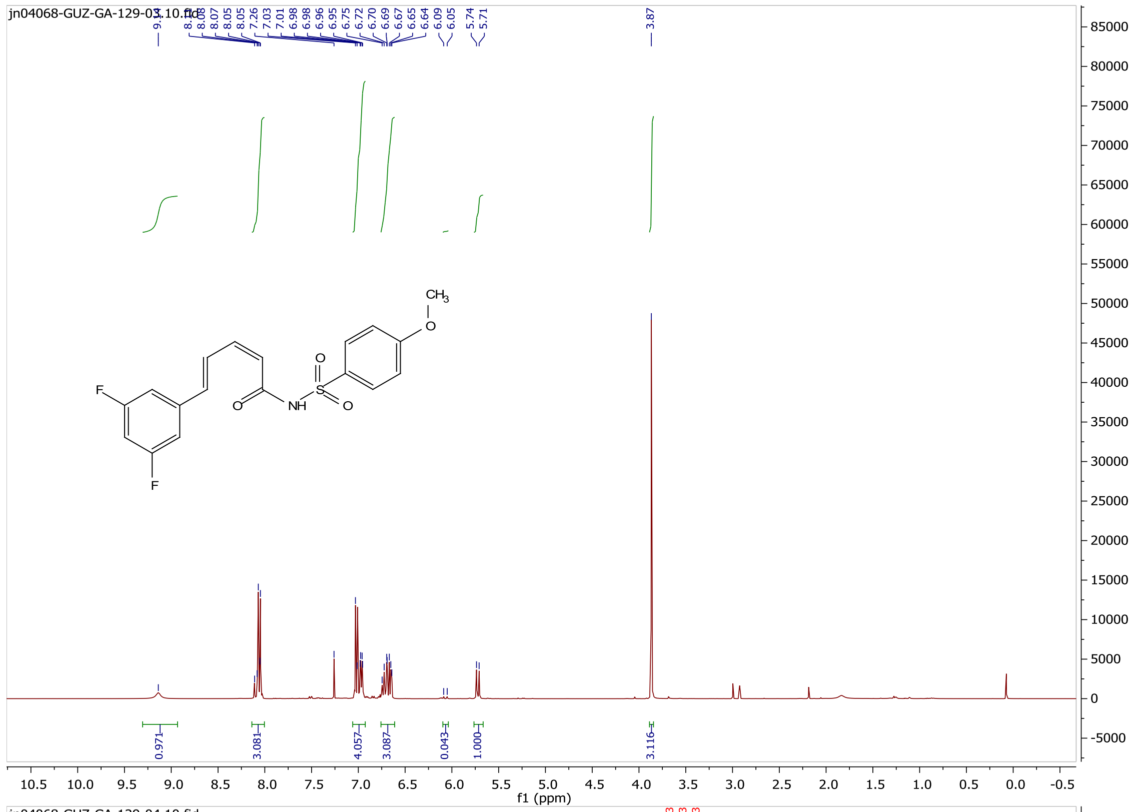
jn04068-GUZ-GA-129-03.10.fid
9.13
8.08
8.07
8.05
8.05
7.26
7.03
7.01
6.98
6.98
6.96
6.95
6.75
6.72
6.70
6.69
6.67
6.65
6.64
6.09
6.05
5.74
5.71
3.87
CH3
O
O
S
F
O
NH
O
F
0.971
3.081
4.057
3.087
0.043
1.000
3.116
f1 (ppm)

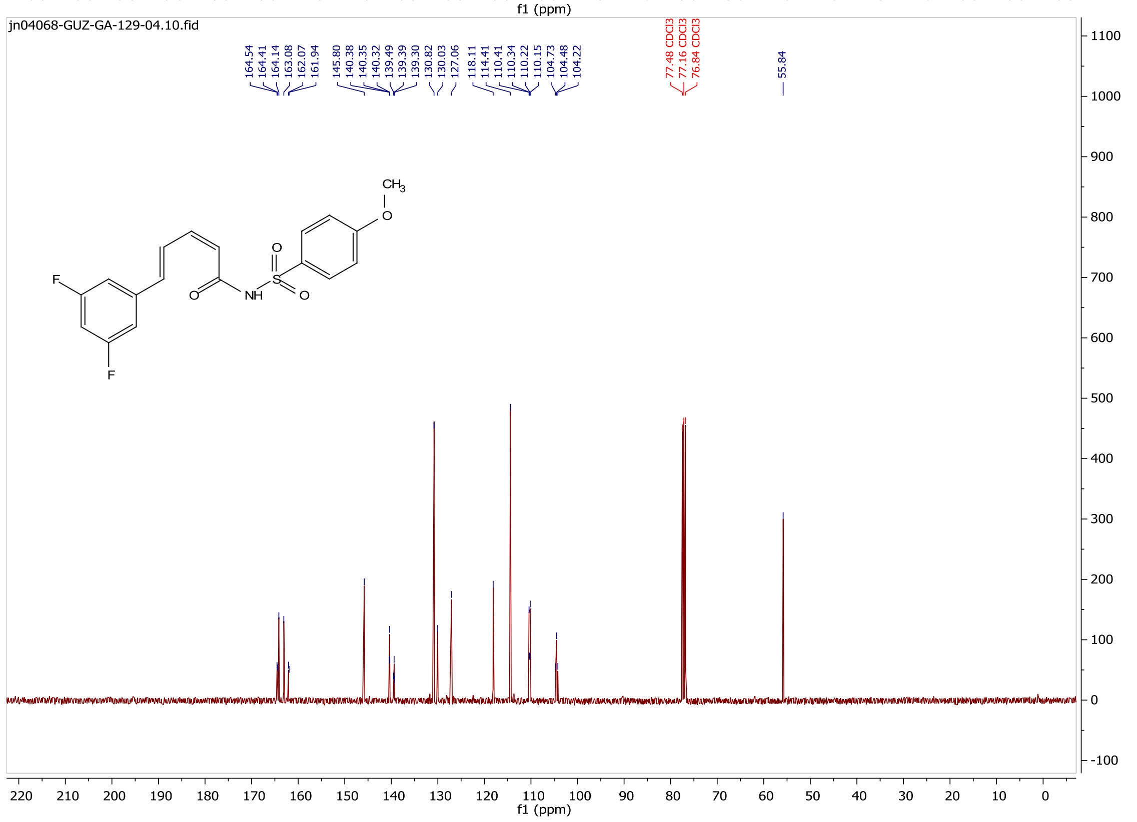
jn04068-GUZ-GA-129-04.10.fid
164.54
164.41
164.14
163.08
162.07
161.94
145.80
140.38
140.35
140.32
139.49
139.39
139.30
130.82
130.03
127.06
118.11
114.41
110.41
110.34
110.22
110.15
104.73
104.48
104.22
77.48 CDCl3
77.16 CDCl3
76.84 CDCl3
55.84
CH3
O
O
S
F
O
NH
O
F
f1 (ppm)

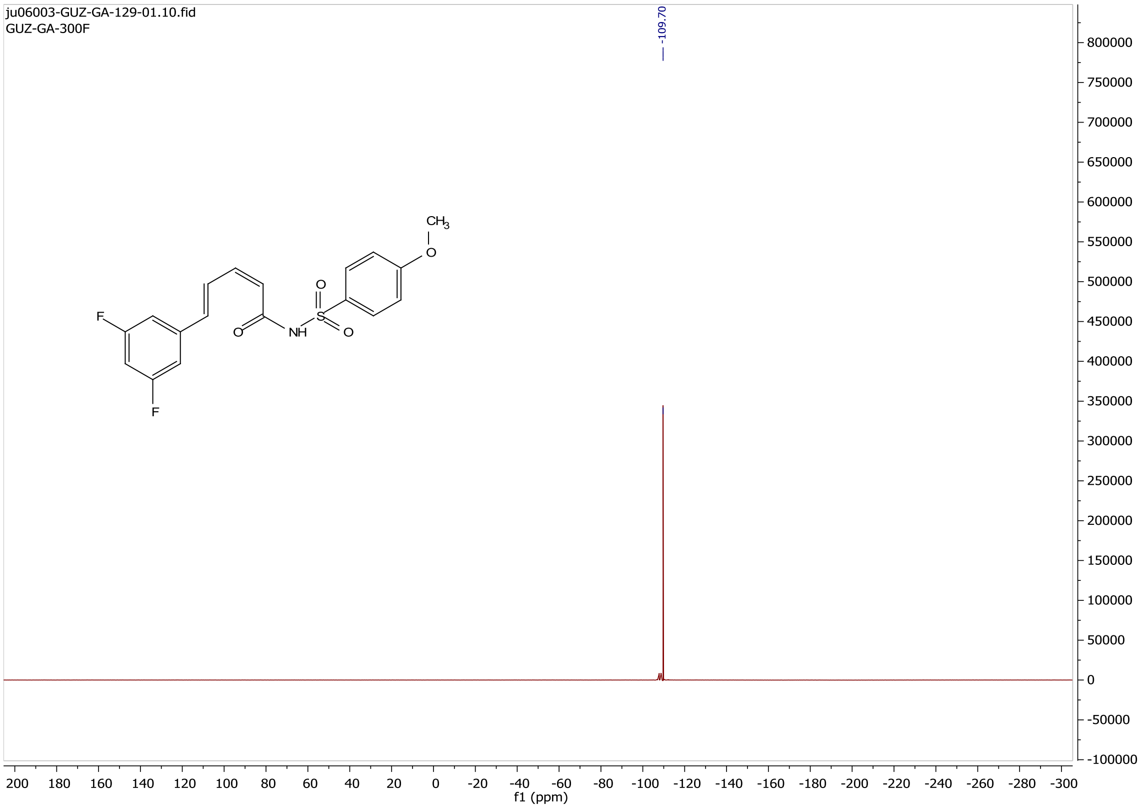
ju06003-GUZ-GA-129-01.10.fid
GUZ-GA-300F
-109.70
F
F
O
NH
S
O
O
O
CH_3
800000
750000
700000
650000
600000
550000
500000
450000
400000
350000
300000
250000
200000
150000
100000
50000
0
-50000
-100000
200 180 160 140 120 100 80 60 40 20 0 -20 -40 -60 -80 -100 -120 -140 -160 -180 -200 -220 -240 -260 -280 -300
f1 (ppm)

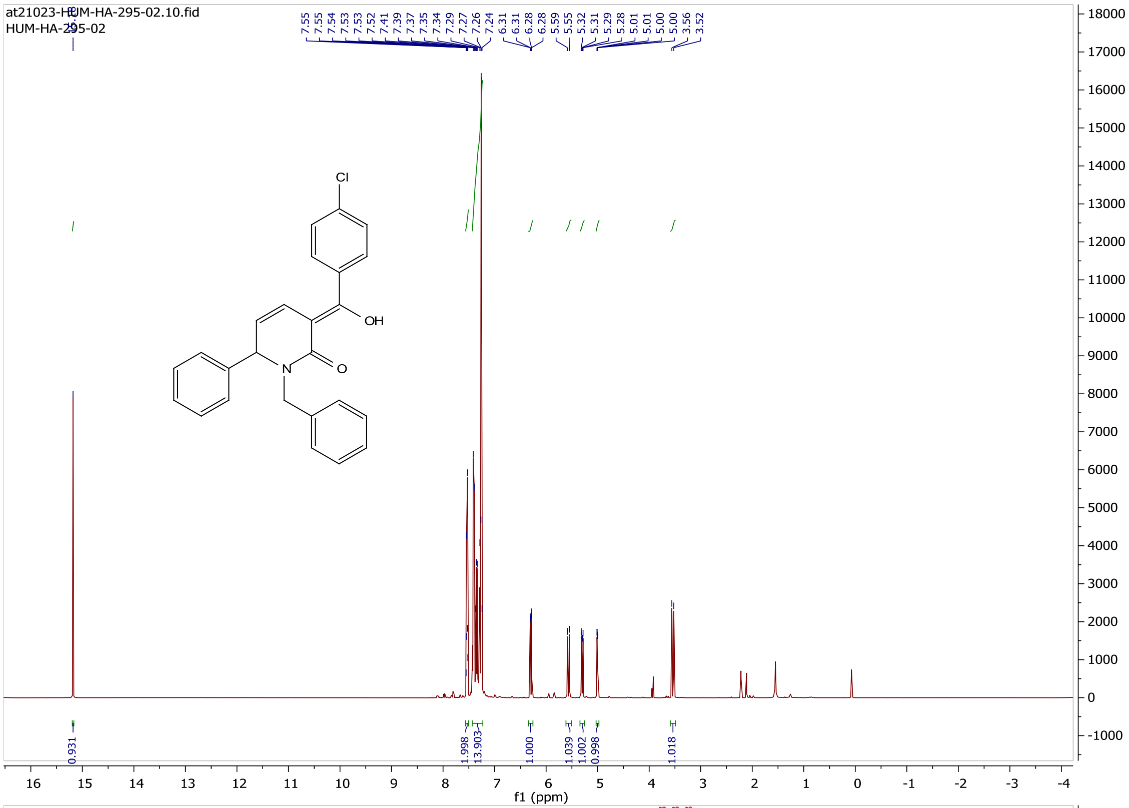
at21023-HUM-HA-295-02.10.fid
HUM-HA-295-02
7.55 7.55 7.54 7.53 7.53 7.52 7.41 7.39 7.37 7.35 7.34 7.29 7.27 7.26 7.24 6.31 6.31 6.28 6.28 5.59 5.55 5.32 5.31 5.29 5.28 5.01 5.01 5.00 5.00 3.56 3.52
Cl
OH
N
O
0.931
1.998
13.903
1.000
1.039
1.002
0.998
1.018
f1 (ppm)

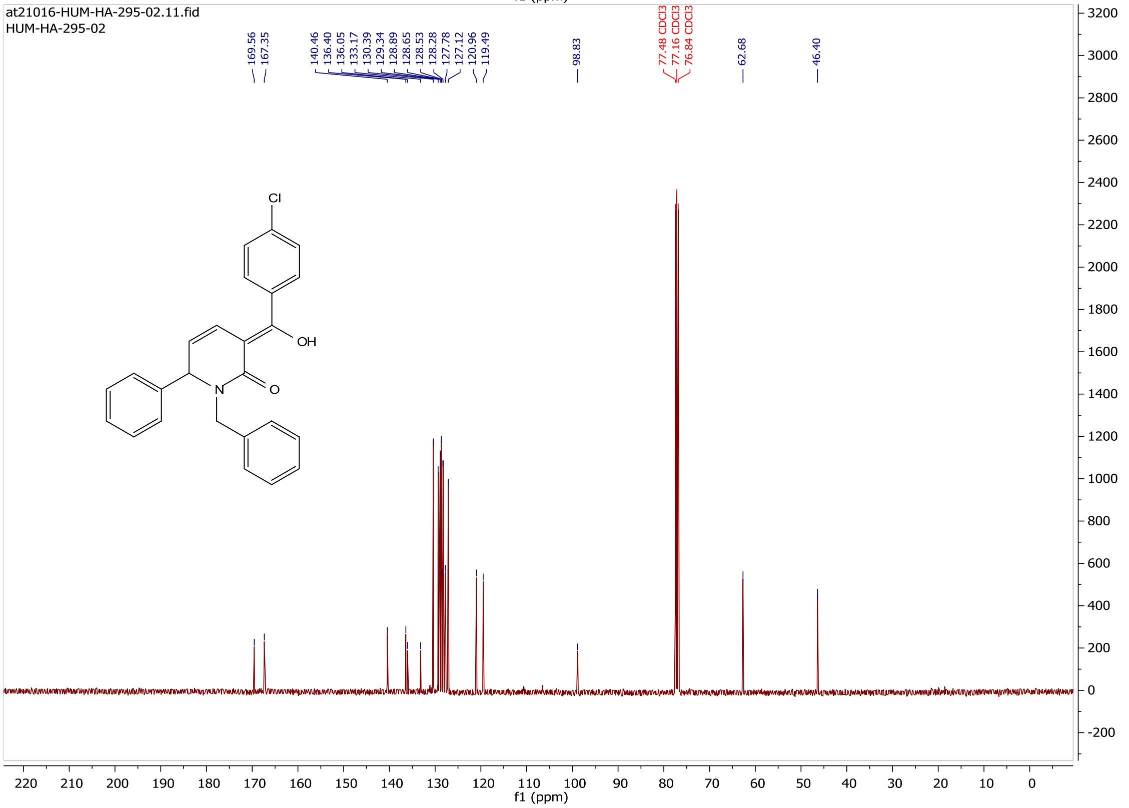
at21016-HUM-HA-295-02.11.fid
HUM-HA-295-02
169.56 167.35
140.46 136.40 136.05 133.17 130.39 129.34 128.89 128.65 128.53 128.28 127.78 127.12 120.96 119.49
98.83
77.48 CDCl3
77.16 CDCl3
76.84 CDCl3
62.68
46.40
Cl
OH
N
O
f1 (ppm)

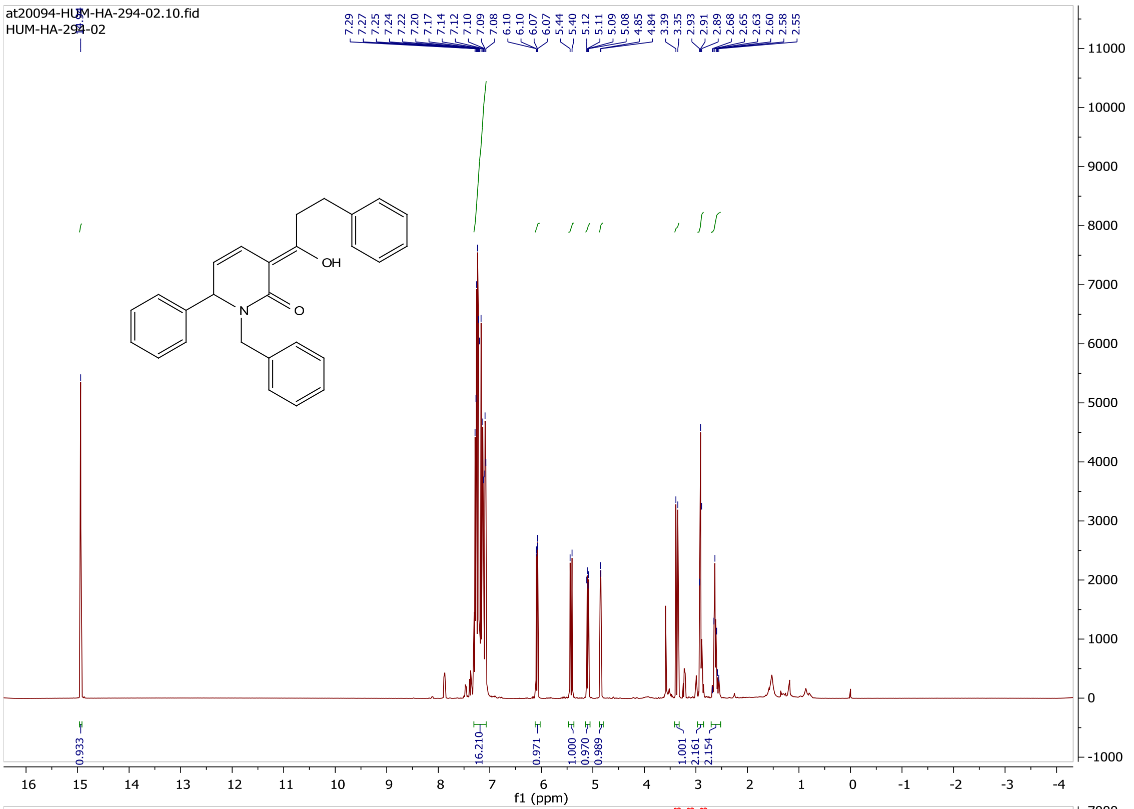

at20094-HUM-HA-294-02.10.fid
HUM-HA-294-02
7.29 7.27 7.25 7.24 7.22 7.20 7.17 7.14 7.12 7.10 7.09 7.08 6.10 6.10 6.07 6.07 5.44 5.40 5.12 5.11 5.09 5.08 4.85 4.84 3.39 3.35 2.93 2.91 2.89 2.68 2.65 2.63 2.60 2.58 2.55
OH
N
O
0.933
16.210
0.971
1.000
0.970
0.989
1.001
2.161
2.154
f1 (ppm)

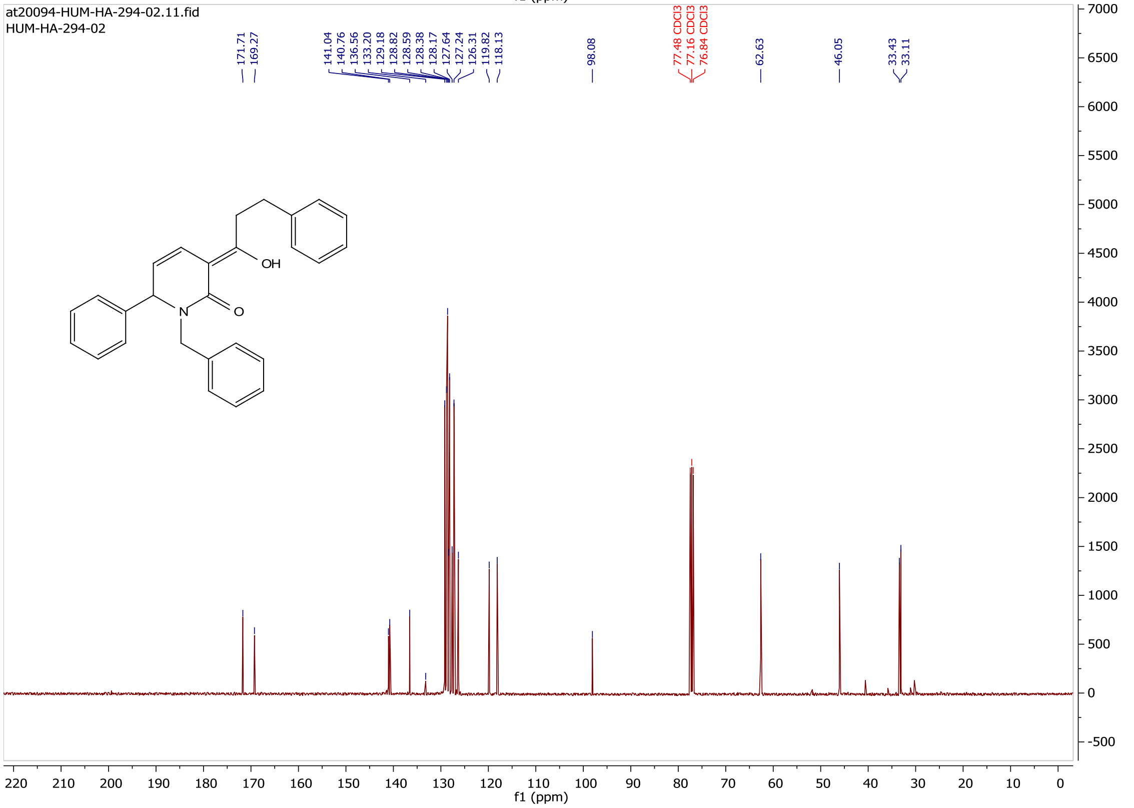

at20094-HUM-HA-294-02.11.fid
HUM-HA-294-02
171.71 169.27
141.04 140.76 136.56 133.20 129.18 128.82 128.59 128.38 128.17 127.64 127.24 126.31 119.82 118.13
98.08
77.48 CDCl3 77.16 CDCl3 76.84 CDCl3
62.63
46.05
33.43 33.11
OH
N
O
f1 (ppm)

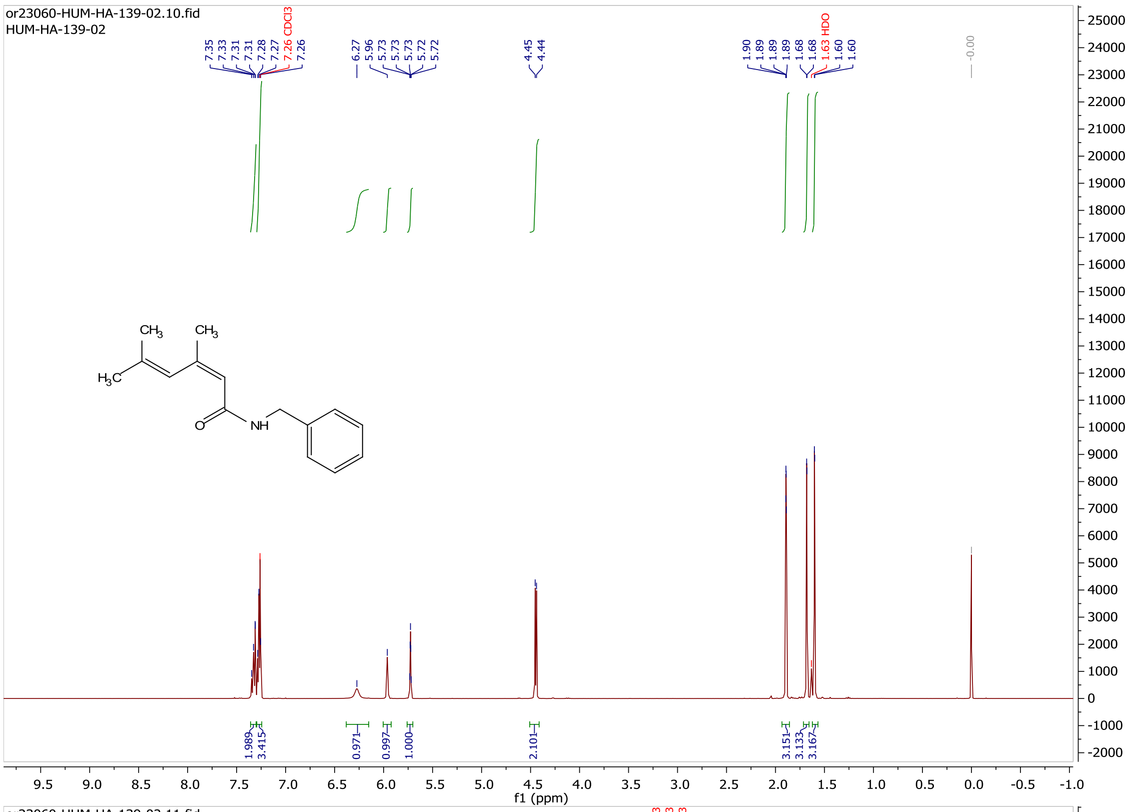
or23060-HUM-HA-139-02.10.fid
HUM-HA-139-02
7.35
7.33
7.31
7.31
7.28
7.27
7.26 CDCl3
7.26
6.27
5.96
5.73
5.73
5.73
5.72
5.72
4.45
4.44
1.90
1.89
1.89
1.89
1.68
1.68
1.63 HDO
1.60
1.60
-0.00
1.989
3.415
0.971
0.997
1.000
2.101
3.151
3.133
3.167
f1 (ppm)

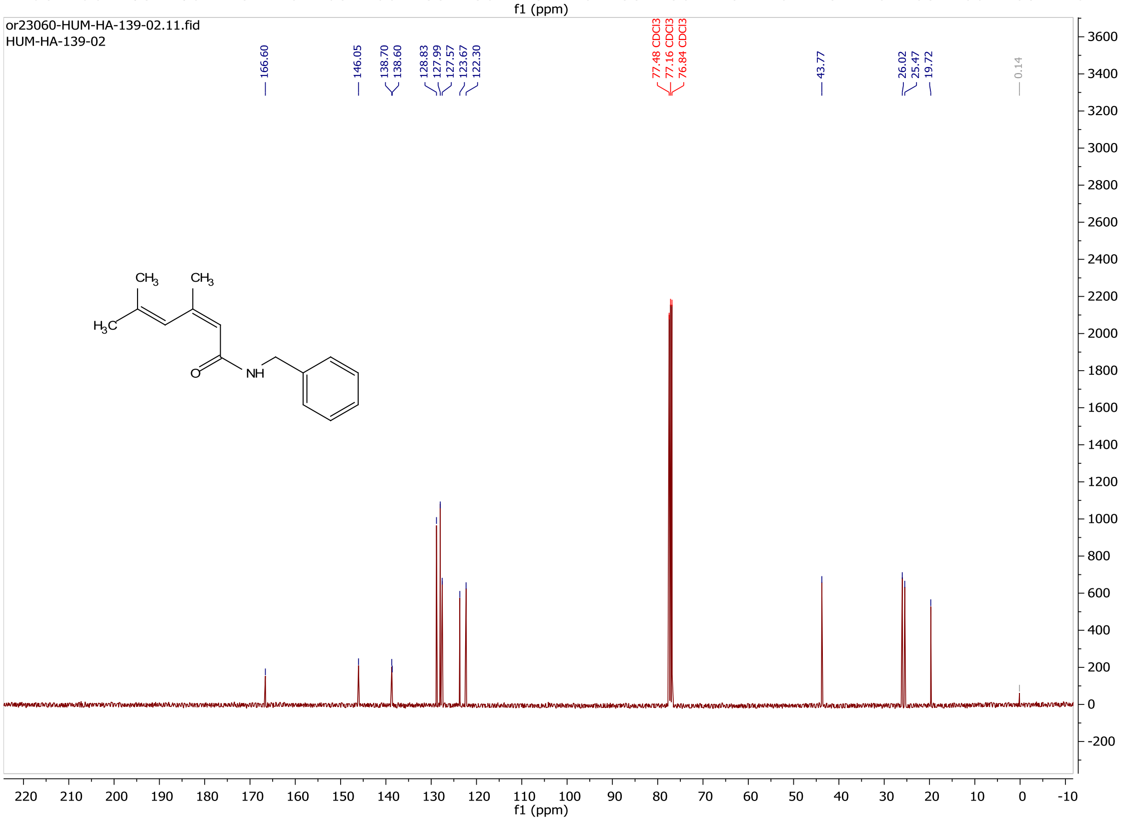
or23060-HUM-HA-139-02.11.fid
HUM-HA-139-02
166.60
146.05
138.70
138.60
128.83
127.99
127.57
123.67
122.30
77.48 CDCl3
77.16 CDCl3
76.84 CDCl3
43.77
26.02
25.47
19.72
0.14
f1 (ppm)

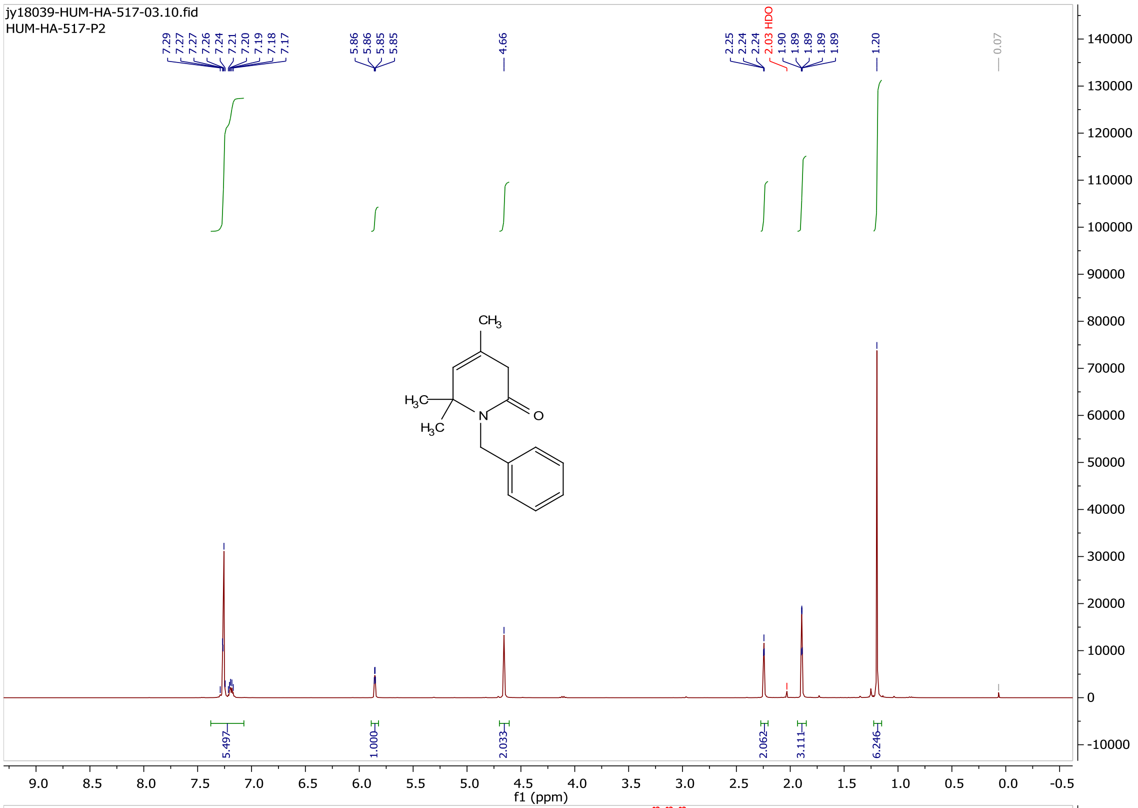
jy18039-HUM-HA-517-03.10.fid
HUM-HA-517-P2
7.29
7.27
7.27
7.26
7.24
7.21
7.20
7.19
7.18
7.17
5.86
5.86
5.85
5.85
4.66
2.25
2.24
2.24
2.03 HDO
1.90
1.89
1.89
1.89
1.89
1.20
0.07
5.497
1.000
2.033
2.062
3.111
6.246
f1 (ppm)

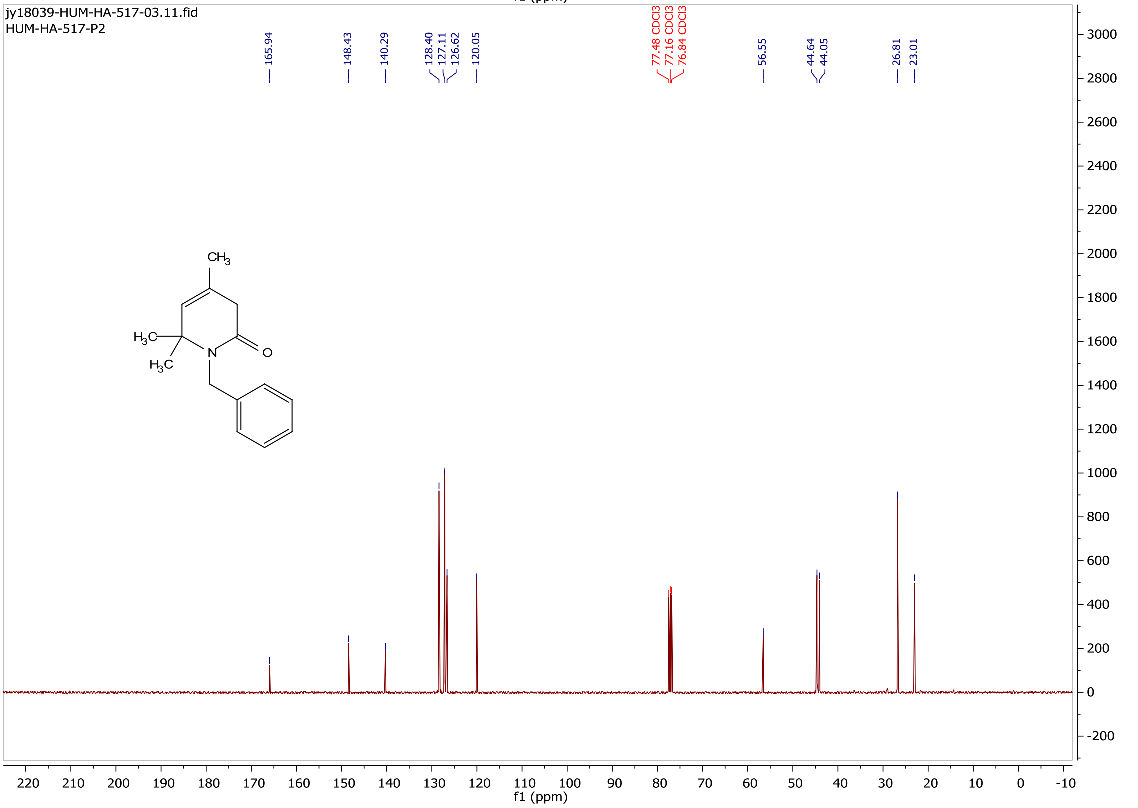
jy18039-HUM-HA-517-03.11.fid
HUM-HA-517-P2
165.94
148.43
140.29
128.40
127.11
126.62
120.05
77.48 CDCl3
77.16 CDCl3
76.84 CDCl3
56.55
44.64
44.05
26.81
23.01
f1 (ppm)

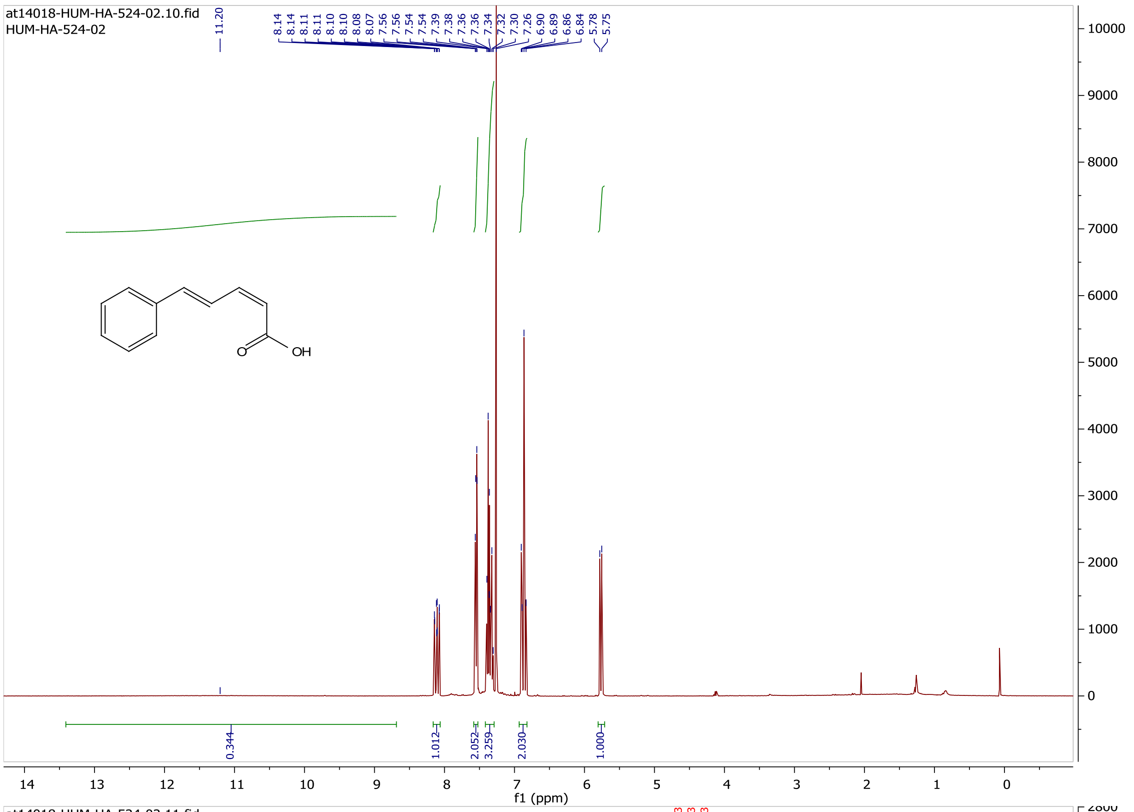
at14018-HUM-HA-524-02.10.fid
HUM-HA-524-02
11.20
8.14 8.14 8.11 8.11 8.10 8.10 8.08 8.07 7.56 7.56 7.54 7.54 7.39 7.38 7.36 7.36 7.34 7.32 7.30 7.26 6.90 6.89 6.86 6.84 5.78 5.75
OH
O
0.344
1.012
2.052
3.259
2.030
1.000
f1 (ppm)

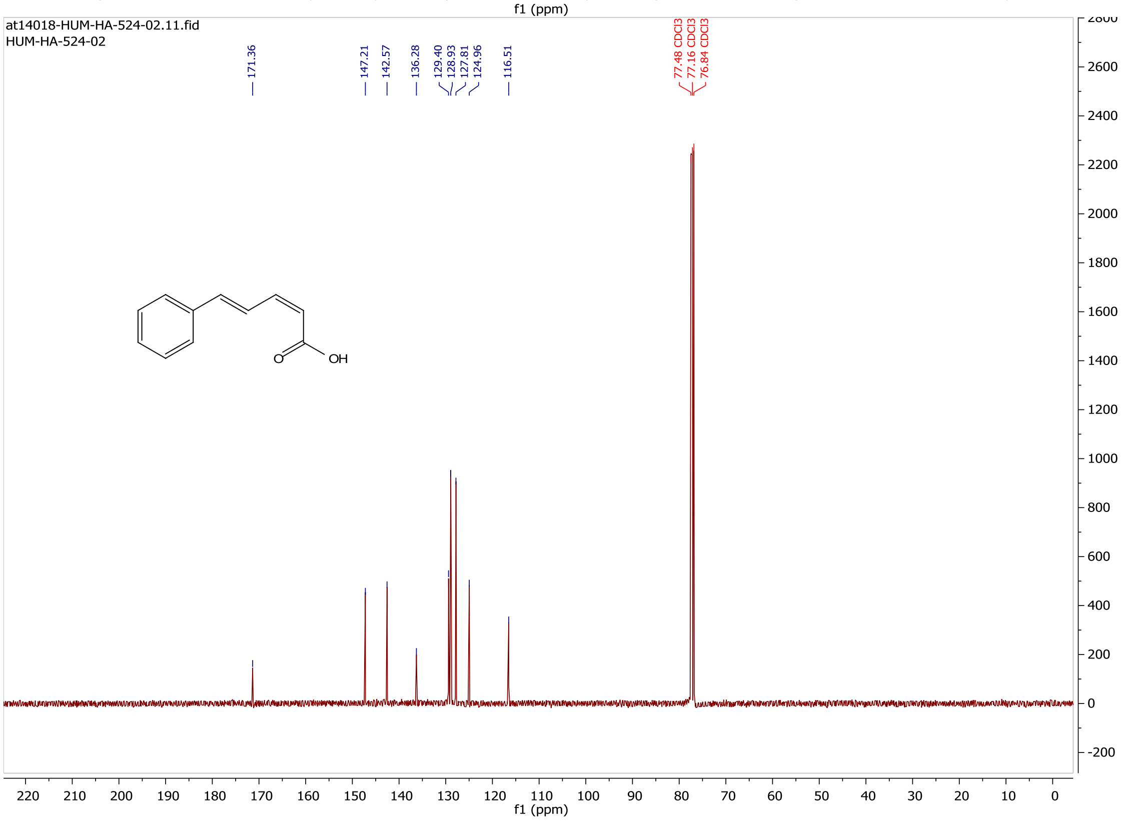
at14018-HUM-HA-524-02.11.fid
HUM-HA-524-02
171.36
147.21
142.57
136.28
129.40 128.93 127.81 124.96
116.51
77.48 CDCl3
77.16 CDCl3
76.84 CDCl3
OH
O
f1 (ppm)

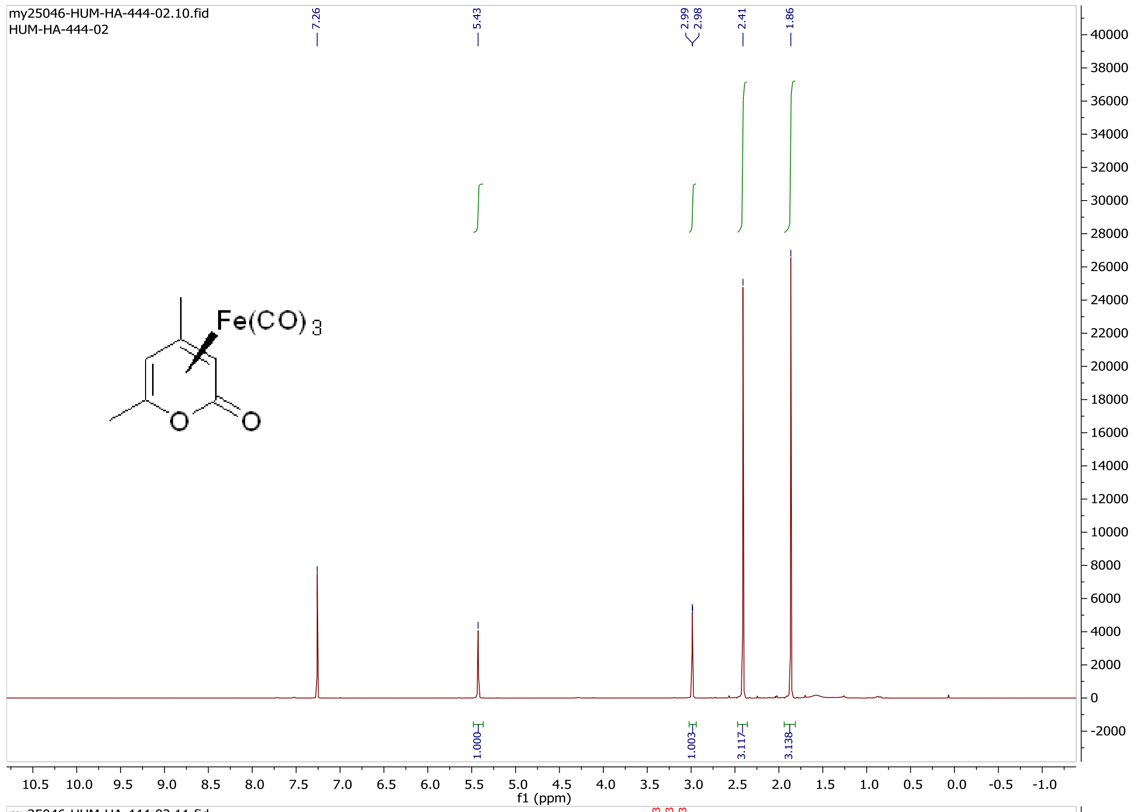
my25046-HUM-HA-444-02.10.fid
HUM-HA-444-02
7.26
5.43
2.99
2.98
2.41
1.86
1.000
1.003
3.117
3.138
Fe(CO)3
f1 (ppm)

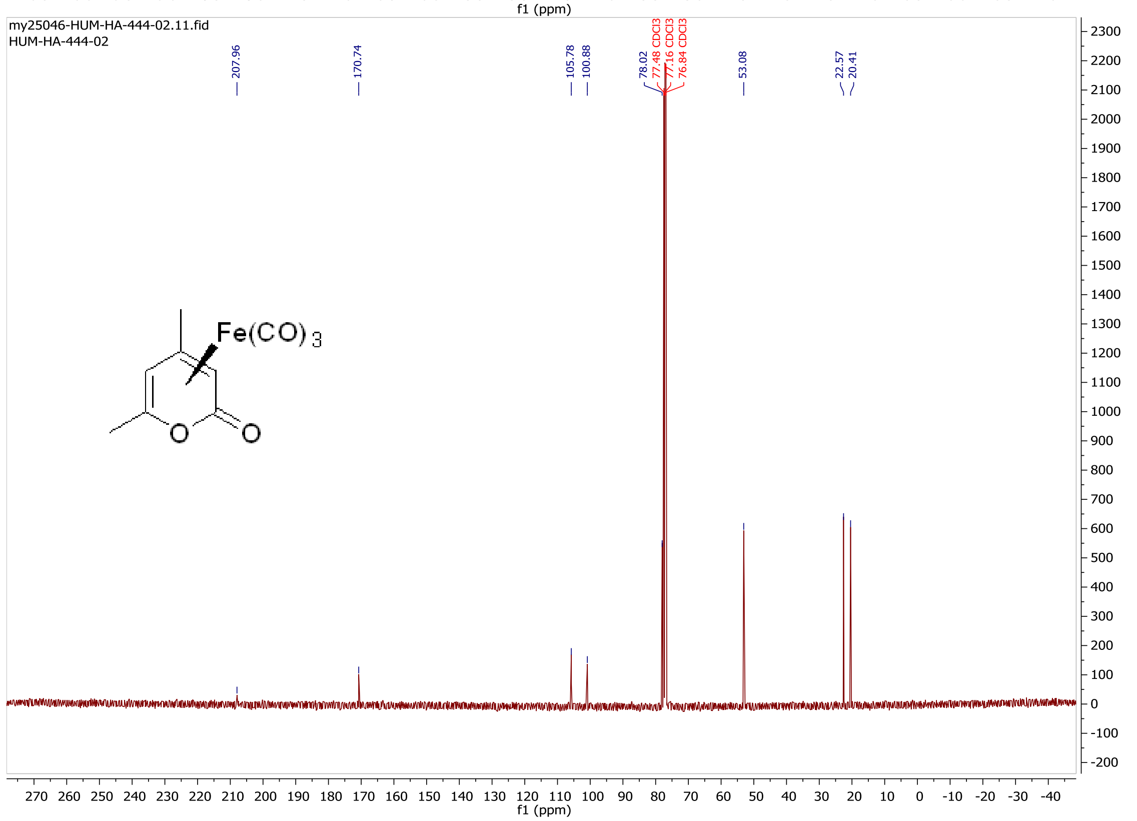
my25046-HUM-HA-444-02.11.fid
HUM-HA-444-02
207.96
170.74
105.78
100.88
78.02
77.48 CDCl3
77.16 CDCl3
76.84 CDCl3
53.08
22.57
20.41
Fe(CO)3
f1 (ppm)

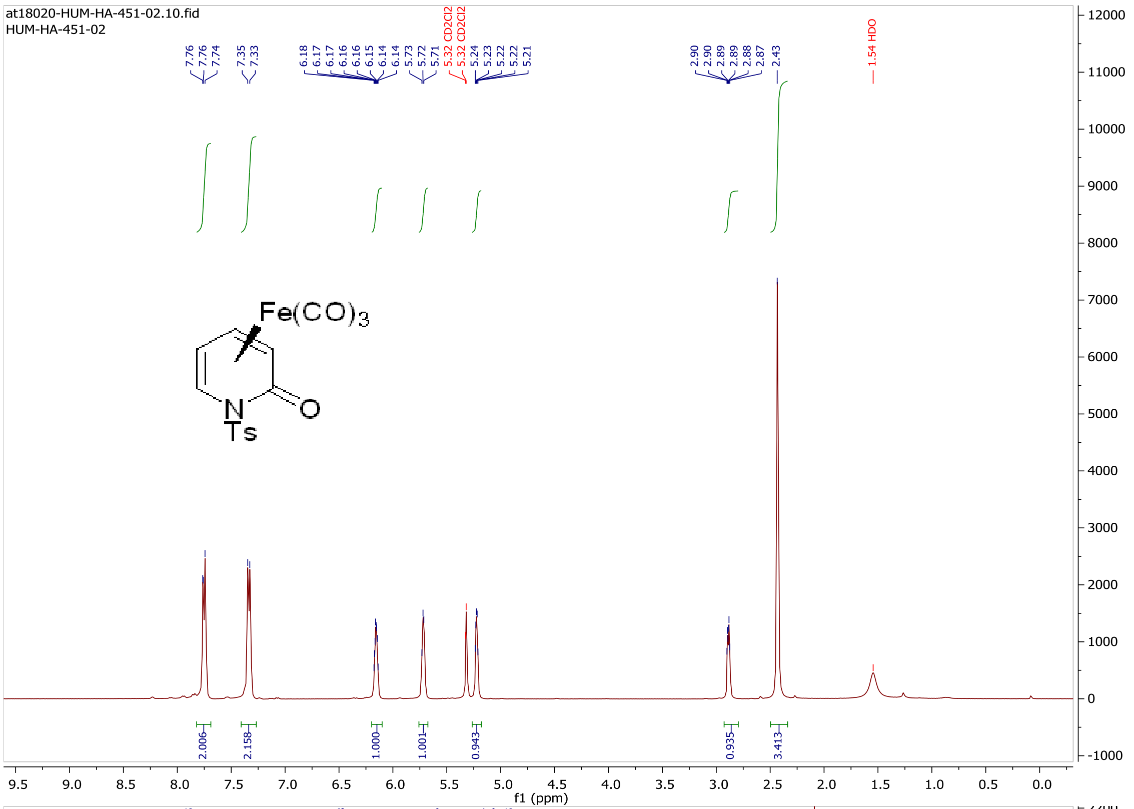
at18020-HUM-HA-451-02.10.fid
HUM-HA-451-02
7.76 7.76 7.74 7.35 7.33 6.18 6.17 6.17 6.16 6.16 6.15 6.14 6.14 5.73 5.72 5.71 5.32 CD2Cl2 5.32 CD2Cl2 5.24 5.23 5.22 5.22 5.21 2.90 2.90 2.89 2.89 2.88 2.87 2.43 1.54 HDO
Fe(CO)3
N
Ts
O
2.006 2.158 1.000 1.001 0.943 0.935 3.413
f1 (ppm)

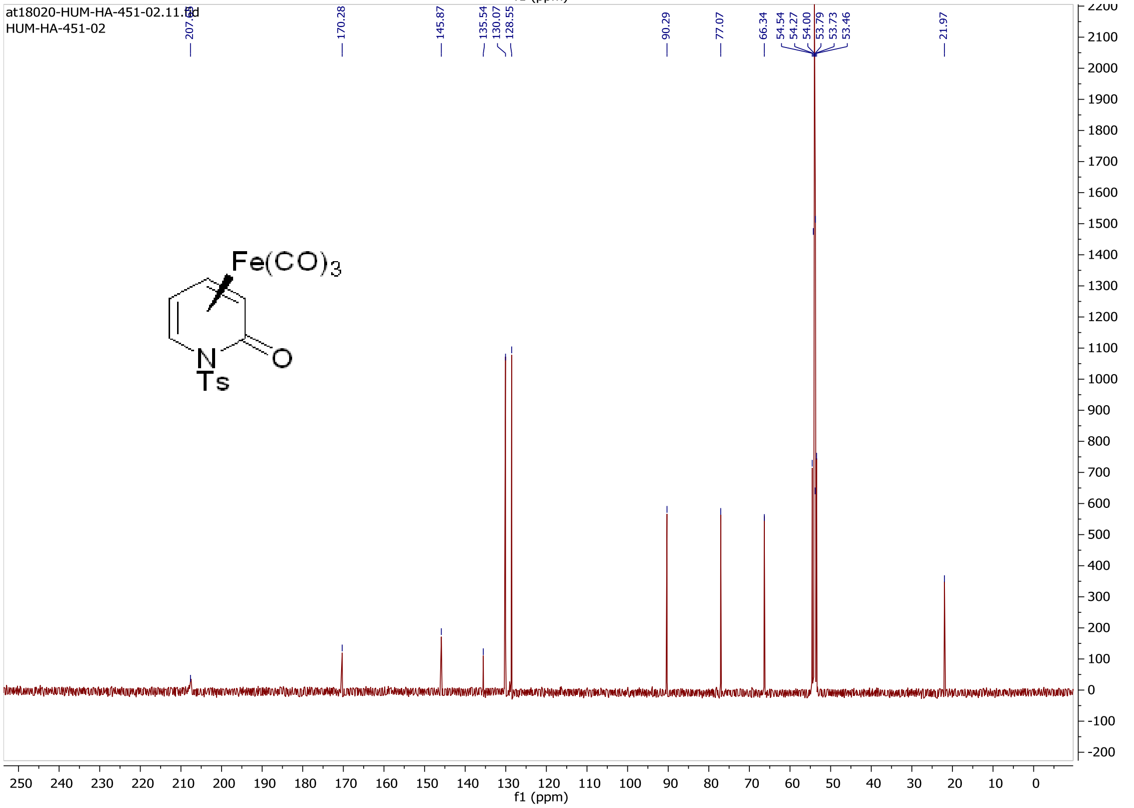
at18020-HUM-HA-451-02.11.fid
HUM-HA-451-02
207.60 170.28 145.87 135.54 130.07 128.55 90.29 77.07 66.34 54.54 54.27 54.00 53.79 53.73 53.46 21.97
Fe(CO)3
N
Ts
O
f1 (ppm)
